# Early dopamine disruption in the entorhinal cortex of a knock-in model of Alzheimer’s disease

**DOI:** 10.1038/s41593-026-02260-w

**Published:** 2026-04-23

**Authors:** Tatsuki Nakagawa, Jiayun L. Xie, Kiwon Park, Kai Cao, Marjan Savadkohighodjanaki, Yutian J. Zhang, Heechul Jun, Ayana Ichii, Jason Y. Lee, Shogo Soma, Yasmeen K. Medhat, Takaomi C. Saido, Kei M. Igarashi

**Affiliations:** 1 Department of Anatomy and Neurobiology, School of Medicine, University of California, Irvine, CA USA; 2 Cognitive Physiology Lab, Graduate School of Medicine, Tohoku University, Sendai, Japan; 3 Department of Biomedical Engineering, Samueli School of Engineering, University of California, Irvine, CA USA; 4 Lab for Proteolytic Neuroscience, RIKEN Center for Brain Science, Wako, Japan; 5 Center for Neural Circuit Mapping, School of Medicine, University of California, Irvine, CA USA; 6 Center for the Neurobiology of Learning and Memory, University of California, Irvine, CA USA; 7 Institute for Memory Impairments and Neurological Disorders, University of California, Irvine, CA USA; 8 Present Address: Department of Molecular Cell Physiology, Kyoto Prefectural University of Medicine, Kyoto, Japan

**Keywords:** Alzheimer's disease, Cortex

## Abstract

The entorhinal cortex is a critical brain area for memory formation, while also the region exhibiting the earliest histological and functional alterations in Alzheimer’s disease (AD). The entorhinal cortex therefore has been long hypothesized as one of the originating brain areas of AD pathophysiology, although circuit mechanisms causing its selective vulnerability remain poorly understood. Here we show that dopamine neurons projecting their axons to the lateral entorhinal cortex (LEC), critical for memory formation in healthy brains, become dysfunctional from the early pathological stage and cause associative memory impairments in amyloid precursor protein knock-in mice. Dopamine dysfunction led to the disruption of associative memory encoding of LEC layer 2/3. Optogenetic reactivation of LEC dopamine fibers rescued associative learning behavior. L-DOPA treatment restored memory encoding of LEC neurons and associative memory of amyloid precursor protein knock-in mice. These results suggest early dysfunction of LEC-projecting dopamine neurons underlie memory impairment in AD from early stages, pointing to a need for clinical investigation of LEC dopamine in patients with AD.

## Abstract (web summary)

The authors reveal entorhinal cortex dopaminergic and behavioral dysfunction in Alzheimer’s model mice, suggesting the possibility that entorhinal dopamine loss may contribute to aspects of Alzheimer’s disease.

## Main

The memory circuit consisting of the entorhinal cortex and hippocampus is critically involved in memory formation and retrieval, and damage to this circuit leads to the impairment of memory formation^1,2^. The entorhinal cortex has been hypothesized as one of the most plausible originating brain regions of Alzheimer’s disease (AD) pathophysiology^3–5^. Past histological studies detailing post mortems of brains with AD have established that the entorhinal cortex has atrophies earlier than the hippocampus, with entorhinal cortex layer 2 neurons being the most vulnerable from the early disease stage^3,6,7^. A subsequent functional magnetic resonance imaging study of from patients with early-stage AD showed that, among the entorhinal–hippocampal circuit, the lateral entorhinal cortex (LEC) suffers the earliest loss of overall activity, earlier than the medial entorhinal cortex^8^. However, it remains unclear why pathophysiological abnormalities of AD appear earliest in the LEC, and what type of brain function is lost from the LEC. In healthy brains, the LEC receives sensory information from olfactory and somatosensory areas^9–11^, exhibiting firing to surrounding objects and items that have olfactory, somatosensory and visual features^12–15^. Using an olfactory item-outcome associative memory task, we recently identified in healthy mice that LEC layer 2a (LEC_L2a_) fan cells represent novel odor cues associated with reward outcome, and this representation is controlled by dopamine inputs to the LEC from the ventral tegmental area (VTA) and substantia nigra pars compacta (SNc)^16^. Since dopamine neurons are known to be intrinsically vulnerable to aging due to their high metabolic stress and elevated mitochondrial oxidant^17–19^, we asked if LEC-projecting dopamine neurons underlie memory deficit symptoms using an AD mouse model.

## Results

### Impaired associative memory formation in amyloid precursor protein knock-in mice

**Fig. 1:**
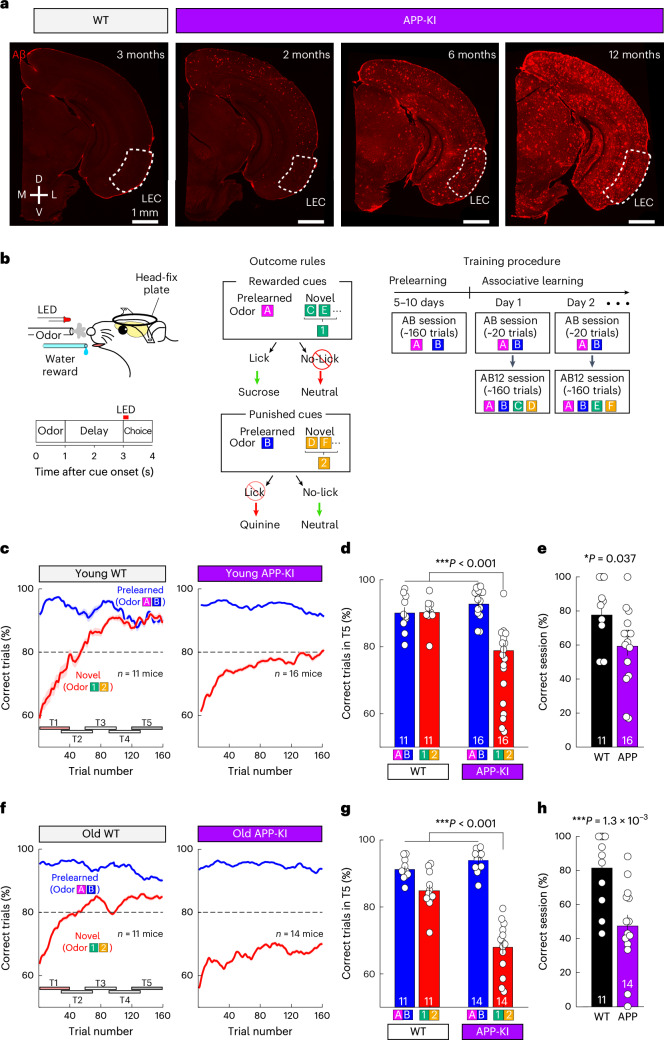
Early impairment of associative memory formation in APP-KI mice. **a**, Coronal section with anti-Aβ immunostaining for 3-month-old WT and 2-, 6- and 12-month-old APP-KI mice. **b**, Head-fixed mice learned associations between odor cues and licking for sucrose water reward. During associative learning sessions, animals were tested with AB-only and AB12 sessions with novel odors (that is, C/D, E/F or G/H). Novel odors are collectively referred to as Odor-1 and Odor-2 in subsequent Figures. **c**, Correct trial rate for prelearned odors (A/B, blue) and novel odors (1/2, red) in young WT mice (3–6 months, left) and young APP-KI mice (3–6 months, right). Timepoints T1–T5 (rectangles) are shown. **d**, Percentage of correct trials in T5 in young WT mice (*n* = 11) versus young APP-KI mice (*n* = 16) (*F*_1,50_ = 20.2, *P* = 4.1 × 10^−5^, odor x strain interaction, ANOVA; *P*_odorA/B-WTvsodor1/2-APP_ = 1.8 × 10^−5^, *P*_odor1/2-WTvsodor1/2-APP_ = 3.1 × 10^−8^, *P*_odorA/B-APPvsodor12-APP_ = 3.1 × 10^−5^, two-sided Tukey post hoc test). **e**, Percentage of sessions where mice correctly learned new association (*t*(25) = 2.19,*P* = 0.037, two-sided unpaired *t*-test). **f**, As in Fig. 1c but for old WT mice (7–11 months, left) and old APP-KI mice (7–11 months, right). **g**, As in Fig. 1d but for old WT mice (*n* = 11) and old APP-KI mice (*n* = 14) (*F*_1,46_ = 41.42, *P* = 6.4 × 10^−8^, odor x strain interaction, ANOVA; *P*_odorA/B-WTvsodor12-APP_ = 1.7 × 10^−3^, *P*_odor1/2-WTvsodor1/2-APP_ = 2.7 × 10^−9^, *P*_odorA/B-APPvsodor1/2-APP_ = 4.3 × 10^−16^, two-sided Tukey post hoc test). **h**, As in Fig. 1e but for old WT mice and old APP-KI mice (*t*(23) = 3.65, *P* = 1.3 × 10^−3^, two-sided unpaired *t*-test). All data are presented as mean ± s.e.m. Statistics details are available in Supplementary Table 1. Statistical significance is indicated as ^*^P < 0.05, ^**^P < 0.01, ^***^P < 0.001. D, dorsal; V, ventral; M, medial; L, lateral.

**Extended Data Fig. 1:**
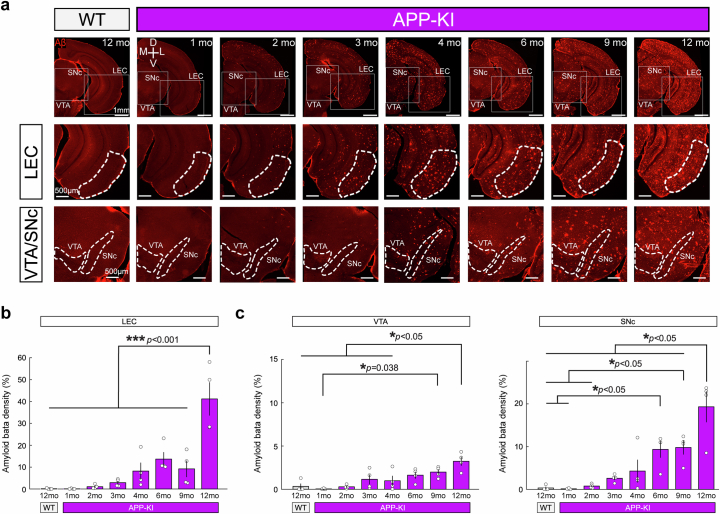
Aβ deposition in APP-KI mice. **a**. Coronal section with anti-Ab immunostaining for 12-month-old WT and 1, 2, 3, 4, 6, 9 and 12-month-old APP-KI mice. D, dorsal, V, ventral, M, medial, L, lateral. **b**. Density of Ab in the LEC of WT and APP-KI mice across ages. (*F*_7,24_ = 3.72, *p* = 1.8e-7, group, ANOVA; *p*_12mo-WTvs12mo-APP_ = 4.1e-7, *p*_1mo-APPvs12mo-APP_ = 4.1e-7, *p*_2mo-APPvs12mo-APP_ = 6.2e-7, *p*_3mo-APPvs12mo-APP_ = 1.4e-6, *p*_4mo-APPvs12mo-APP_ = 1.6e-5, *p*_6mo-APPvs12mo-APP_ = 2.3e-4, *p*_9mo-APPvs12mo-APP_ = 2.6e-5, two-sided Tukey post hoc test; *n* = 4 mice from each age group). **c**. Same as Extended Data Fig. 1b but for density of Ab in the VTA (left) and SNc (right) (VTA: *F*_7,24_ = 13.18, *p* = 7.4e-7, group, ANOVA; *p*_12mo-WTvs12mo-APP_ = 6.6e-4, *p*_1mo-APPvs9mo-APP_ = 0.038, *p*_1mo-APPvs12mo-APP_ = 1.9e-4, *p*_2mo-APPvs12mo-APP_ = 5.3e-4, *p*_3mo-APPvs12mo-APP_ = 0.022, *p*_4mo-APPvs12mo-APP_ = 0.011, two-sided Tukey post hoc test. SNc: *F*_7,24_ = 13.18, *p* = 7.4e-7, group, ANOVA; *p*_12mo-WTvs6mo-APP_ = 0.035, *p*_12mo-WTvs9mo-APP_ = 0.024, *p*_12mo-WTvs12mo-APP_ = 3.7e-6, *p*_1mo-APPvs6mo-APP_ = 0.029, *p*_1mo-APPvs9mo-APP_ = 0.02, *p*_1mo-APPvs12mo-APP_ = 3.1e-6, *p*_2mo-APPvs9mo-APP_ = 0.035, *p*_2mo-APPvs12mo-APP_ = 5.5e-6, *p*_3mo-APPvs12mo-APP_ = 2.8e-5, *p*_4mo-APPvs12mo-APP_ = 1.3e-4, *p*_6mo-APPvs12mo-APP_ = 0.015, *p*_9mo-APPvs12mo-APP_ = 0.026, two-sided Tukey post hoc test; *n* = 4 mice from each age group). Statistics details are also available in Supplementary Table 1. All data are presented as mean ± s.e.m.

To investigate the pathophysiology of LEC, we used amyloid precursor protein knock-in (APP-KI) mice^20,21^. The APP-KI mouse recapitulates the gradual process of AD pathological progression more accurately than aggressive transgenic AD mouse models. In these mice, age-dependent accumulation of amyloid-ß (Aβ) starts widely in cortical regions, including the LEC, at 2 months of age (Fig. 1a and Extended Data Fig. 1). We tested mice’s memory formation using an LEC-dependent olfactory cue-outcome associative memory task^16,22^ (Fig. 1b). In this task, mice use prelearned knowledge to rapidly form associative memories between novel odor cues and their outcomes. Mice were initially trained with prelearning sessions, where they learned to lick after Odor-A for sucrose water reward, and to withhold licking after Odor-B to avoid quinine water punishment. After mice learned to discriminate at >80% correct performance, daily associative learning sessions were tested, where Odors-A, Odors-B and a pair of novel odors (Odor-1 → sucrose, Odor-2 → quinine) were randomly presented. Associative learning was tested daily in individual mice using novel rewarded odors (that is, Odors-C, -E and -G; collectively termed Odor-1) and novel punished odors (that is, Odors-D, -F and -H; termed Odor-2). This associative learning was previously shown to be dependent on the activity of LEC_L2a_ neurons as well as the dopamine inputs to the LEC^16^.

Since the APP-KI mouse (APP^NL-G-F/NL-G-F^) is on the C57BL/6 background, we used APP^WT/WT^ C57BL/6 mice as a control (referred to as WT mice). Consistent with our previous result, control WT mice at 3–6 months (defined as young WT mice) rapidly learned new associations by trial and error within a 160-trial session (red trace, Fig. 1c, left), while continuously retrieving prelearned Odors-A/B associations with >80% correct rate throughout a session (blue trace, Figs. 1c (left) and 1d). By contrast, 3–6 month APP-KI mice (young APP-KI mice) showed decreased and slower learning performance for new associations, barely reaching the 80% criteria (Figs. 1c (right), 1d and Supplementary Fig. 1). We divided the session into five timepoints^16^ (T1–T5, Fig. 1c) and compared the new performance associations between WT and APP-KI mice in T5 (Fig. 1d). Young WT mice performed at 90.6 ± 0.9% in T5, whereas young APP-KI performed at 78.6 ± 2.1% (*F*_1,50_ = 20.2, *P* = 4.1 × 10^−5^, odor × strain interaction, ANOVA; *P* = 3.1 × 10^−8^, post hoc Tukey test; *n* = 11 young WT and *n* = 16 young APP-KI mice). We defined sessions with >= 80% performance for new associations as correct sessions, and sessions with <80% performance as error sessions^16^. With this criterion, we previously found in healthy mice that the cue-reward associative representation of LEC_L2a_ neurons and dopamine activity in the LEC were present in correct sessions, while they were lost in error sessions^16^. Young WT mice had correct sessions at 77.5 ± 6.1% among all tested sessions, whereas young APP-KI mice had correct sessions at 59.1 ± 5.5% (*P* = 0.037, unpaired *t*-test; Fig. 1e). At the older age point of 7–11 months, APP-KI mice (defined as old APP-KI mice, *n* = 14 mice; mean age 9.6 ± 0.4 months) showed further impairment in associative memory formation compared to age-matched old WT mice (*n* = 11 mice; mean age 8.8 ± 0.4 months) (84.9 ± 1.8% correct trials for old WT mice versus 67.9 ± 2.0% for old APP-KI mice; *F*_1,46_ = 41.4,*P* = 6.4 × 10^−8^, odor × strain interaction, ANOVA; *P* = 2.7 × 10^−9^, post hoc Tukey test; Fig. 1g). Notably, old WT mice showed a slight decline of associative memory compared to young WT mice (Supplementary Fig. 1c, *F*_1,40_ = 30.1, *P* = 0.031, odor × age interaction, ANOVA; *P* = 0.038, post hoc Tukey test; *n* = 11 young WT and *n* = 11 old WT mice). Old WT mice correctly learned 81.3 ± 6.4% of all sessions, whereas APP-KI correctly learned 47.3 ± 6.5% (*P* = 1.3 × 10^−3^, unpaired *t*-test; Fig. 1h). In contrast, both young and old APP-KI mice showed intact performance for retrieving prelearned associative memories (Odors-A/B, blue traces in Figs. 1c and 1f; Figs. 1d and 1g, young WT versus APP-KI mice: *P* = 0.92, post hoc Tukey test; old WT versus APP-KI mice *P* = 0.61, post hoc Tukey test), suggesting that olfactory perception and retrieval of prelearned memory are both spared in APP-KI mice. APP-KI and WT mice showed comparable duration for the training with prelearning sessions using Odor-A and Odor-B (*F*_1,48_ = 2.2 × 10^−3^, *P* = 0.96, strain × age interaction, ANOVA; post hoc Tukey test *P* = 0.93 for young APP-KI versus young WT mice, *P* = 0.99 for old APP-KI versus old WT mice**;** Supplementary Fig. 1f). Together, these results demonstrate that APP-KI mice show progressive impairment in the formation, but not the retrieval, of associative memory starting at 4 months. Interestingly, the impairment specific to the formation, but not the retrieval, of associative memory is similar to our previous result observed when LEC_L2a_ neurons or LEC_L5/6_ neurons were optogenetically inhibited^16,22^.

### Disrupted associative memory encoding in lateral entorhinal cortex layer 2/3 neurons

**Fig. 2:**
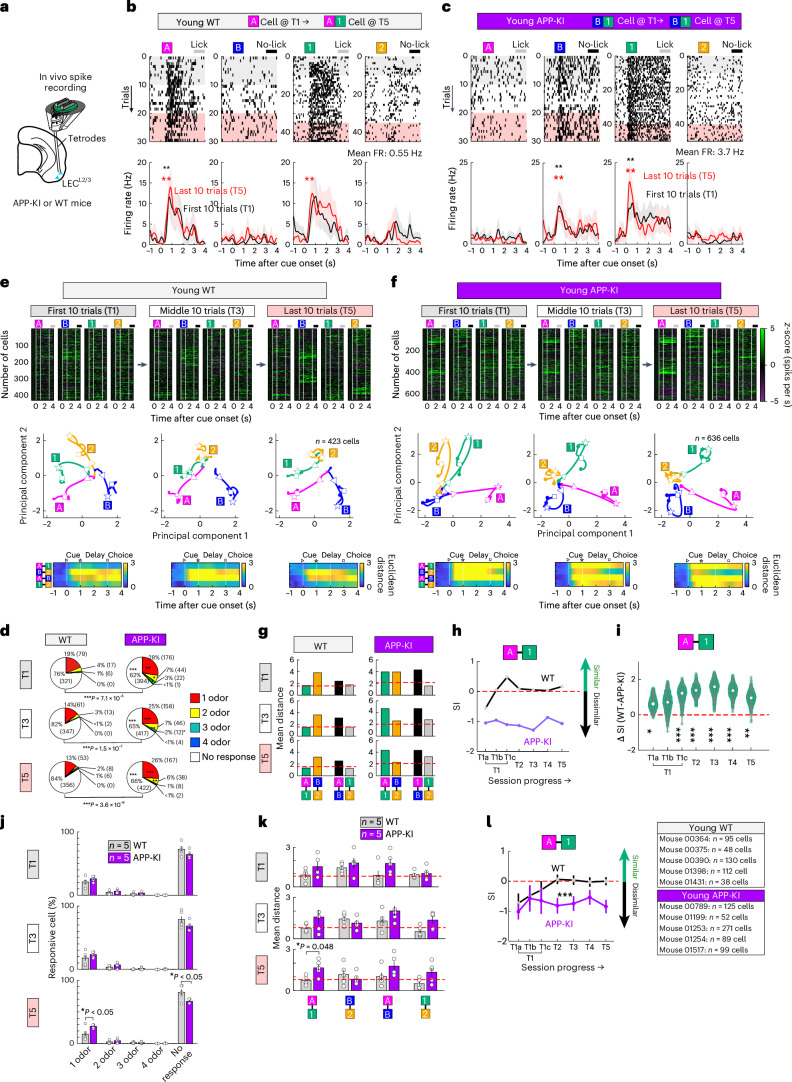
Disrupted cue-reward association of LEC_L2/3_ neurons in young APP-KI mice. **a**, A schematic diagram of the in vivo spike recording experiment. **b**, An example LEC_L2/3_ cell in a young WT mouse from a correct session, showing firing for Odor-A and developing firing for Odor-1 (mean ± s.e.m.; ^**^P < 0.01 during cue-delay period compared to precue period, *P*_odorA-first_ = 2.0 × 10^−3^, *P*_odorA-last_ = 3.9 × 10^−3^, *P*_odor1-last_ = 2.0 × 10^−3^, two-sided Wilcoxon signed-rank test). Black trace, first 10 trials; red trace, last 10 trials. **c**, An example LEC_L2/3_ cell in a young APP-KI mouse from an error session exhibited constant firing for Odor-A with no change in its firing pattern (mean ± s.e.m.; ^**^P < 0.01 during cue-delay period compared to precue period, *P*_odorB-first_ = 2.0 × 10^−3^, *P*_odorB-last_ = 3.7 × 10^−3^, *P*_odor1-first_ = 2.0 × 10^−3^, *P*_odor1-last_ = 2.1 × 10^−3^, two-sided Wilcoxon signed-rank test). **d**, Percentage of LEC_L2/3_ cells classified by odor cue response in the first 10 trials (T1, top), middle 10 trials (T3, middle) and last 10 trials (T5, bottom) (*P*_T1_ = 7.1 × 10^−5^, *P*_T3_ = 1.5 × 10^−7^, *P*_T5_ = 3.6 × 10^−9^, Chi-squared test for distributions between WT and APP-KI mice; *P*_1odor-T1_ = 1.1 × 10^−3^, *P*_2odor-T1_ = 0.029, *P*_3odor-T1_ = 0.029, *P*_noresponsive-T1_ = 4.4 × 10^−6^, *P*_1odor-T3_ = 5.4 × 10^−5^, *P*_2odor-T3_ = 3.2 × 10^−3^, *P*_3odor-T3_ = 0.03, *P*_noresponsive-T3_ = 9.7 × 10^−9^, *P*_1odor-T5_ = 9.2 × 10^−8^, *P*_2odor-T5_ = 1.1 × 10^−3^, *P*_noresponsive-T5_ = 2.5 × 10^−10^, Benjamini–Hochberg post hoc test for each responsive class between WT and APP-KI mice; *n* = 423 cells from young WT and *n* = 636 cells from young APP-KI mice). Percentages of neurons in each responsive class is shown, and numbers in parentheses denote number of neurons in each responsive class. **e**, Top: spike firing rate of *n* = 423 LEC_L2/3_ cells from young WT mice, shown in *z*-score during first 10 (T1), middle 10 (T3) and last 10 (T5) trials of the session. Middle: time-resolved PCA trajectory of LEC_L2/3_ cell activity for each odor type (⊳, cue onset; ✩, cue offset/delay onset; ☐, delay offset). Bottom: Euclidean distance between odor types. **f**, As in Fig. 3e but for young APP-KI mice (*n* = 636 cells). Trajectories for Odor-A and Odor-1 kept far apart through T1 to T5. **g**, Mean Euclidean distance between PCA trajectories during 0.5–1.5 s after cue onset in young WT (left) and young APP-KI mice (right). The 95th percentile distance obtained from shuffled data denotes significant distance (red line). **h**, SI between Odor-A and Odor-1 in young WT and young APP-KI mice across learning. Positive SI denotes similar representations between Odor-A and Odor-1, whereas negative SI denotes dissimilar representations. T1 is subdivided into T1a (trials 1–3), T1b (trials 4–6) and T1c (trials 7–9). **i**, SI of young WT and young APP-KI mice was compared using bootstrapping method (Methods). SI was calculated for 1,000 bootstraps, then SIs for WT mice were subtracted by SIs for APP-KI mice. The subtraction confirms higher similarity in the representations between Odor-A and Odor-1 in WT mice compared to that in APP-KI mice (*P*_T1a_ = 0.023, *P*_T1c_ = 8.7 × 10^−4^, *P*_T2_ = 6.6 × 10^−7^, *P*_T3_ = 1.7 × 10^−5^, *P*_T4_ = 8.7 × 10^−6^, *P*_T5_ = 1.1 × 10^−3^, *n* = 1,000 two-sided bootstrapping test). **j**, Percentage of LEC_L2/3_ cells per mouse, classified by odor cue response in the first 10 trials (T1, top; *F*_4,40_ = 2.7, *P* = 0.043, responsive type × strain, ANOVA), middle 10 trials (T3, middle; *F*_4,40_ = 2.5, *P* = 0.057, responsive type × strain, ANOVA) and last 10 trials (T5, bottom; *F*_4,40_ = 8.0, *P* = 7.7 × 10^−5^, responsive type × strain, ANOVA; *P*_*1odor*_ = 0.021, *P*_noresponsecell_ = 4.3 × 10^−3^, two-sided Tukey post hoc test) (*n* = 5 mice per strain: young WT and young APP-KI mice). Data are presented as mean ± s.e.m. **k**, Mean Euclidean distance between PCA trajectories during 0.5–1.5 s after cue onset, averaged across young WT and young APP-KI mice (*n* = 5 each). The 95th percentile distance obtained from shuffled data denotes significant distance (red line) (A-1 mean distance, *P*_*T5*_ = 0.048, two-sided unpaired *t*-test followed by false discovery rate (FDR) correction for multiple comparison; mean ± s.e.m.). **l**, Mean SI between Odor-A and Odor-1 in young WT and young APP-KI mice across learning (mean ± s.e.m.; *n* = 5 each, *F*_1,56_ = 14.8, *P* = 3.1 × 10^−4^, strain, ANOVA). Positive SI denotes similar representations between Odor-A and Odor-1, whereas negative SI denotes dissimilar representations. Statistics details are available in Supplementary Table 1. Statistical significance is indicated as ^*^P < 0.05, ^**^P < 0.01, ^***^P < 0.001.

**Extended Data Fig. 2:**
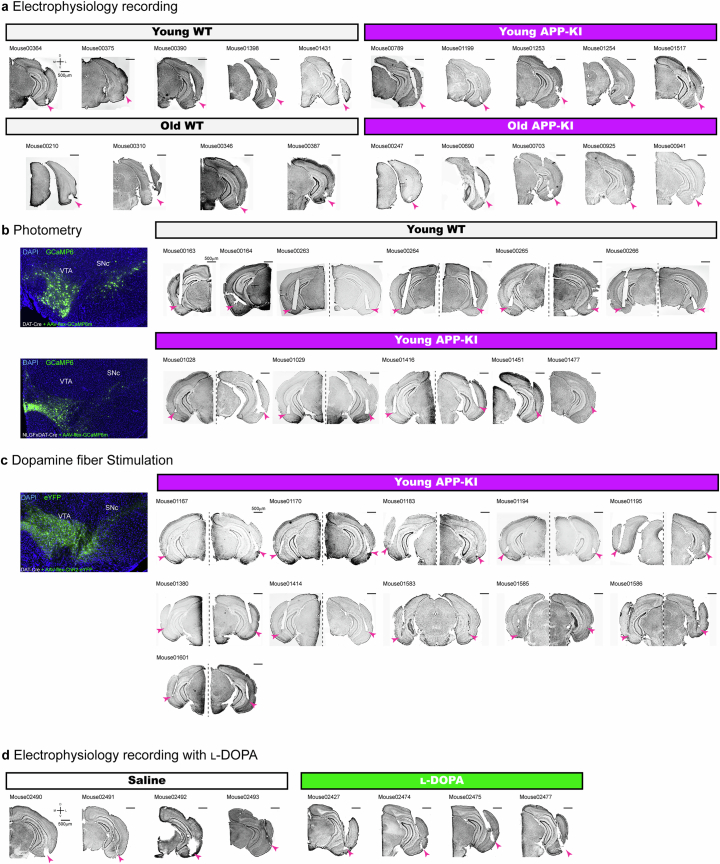
Histological validation of implanted sites. **a**. Recording positions in the LEC of WT and APP-KI mice for electrophysiology recording experiments (Fig. 2, Extended Data Fig. 3–9, and Supplementary Fig. 2–5). Tetrode tips obtained using electrolytic lesioning were marked with arrowheads. D, dorsal, V, ventral, M, medial, L, lateral. **b**. (Left) Representative sections from *n* = 6 recorded WT mice and *n* = 5 recorded APP-KI mice showing GCaMP-expressing dopaminergic neurons in the VTA and SNc. (Right) Optic fiber positions in the LEC of young DAT-Cre and young APP-KI x DAT-Cre mice injected with AAV-flex-GCaMP6m into the VTA and SNc for photometry experiments (Fig. 4). Arrowheads show estimated tips of optic fibers. Four mice received unilateral implantations and eight mice received bilateral implantations. **c**. (Left) Representative sections from *n* = 10 stimulated APP-KI mice showing ChR2-expressing dopaminergic neurons in the VTA and SNc. (Right) Optic fiber positions in the LEC of APP-KI x DAT-Cre mice injected with AAV-flex-ChR2-eYFP or AAV-flex-ChR2-mCherry into the VTA and SNc for stimulation of LEC-projecting VTA/SNc dopaminergic fibers in LEC (Fig. 5). **d**. Recording positions in the LEC of APP-KI mice for electrophysiology recording experiments with L-DOPA (Fig. 6). Tetrode tips obtained using electrolytic lesioning were marked with arrowheads. D, dorsal, V, ventral, M, medial, L, lateral.

**Extended Data Fig. 3:**
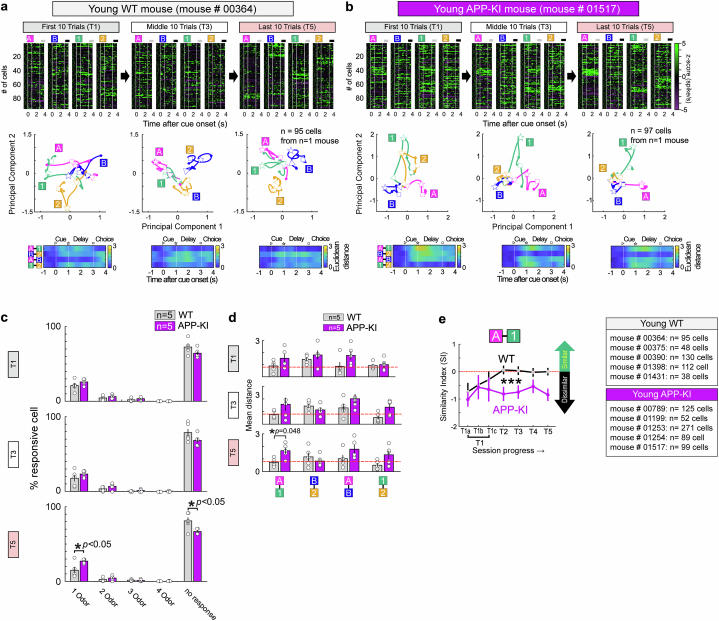
Principal component analyses of LEC_L2/3_ neurons of young WT and young APP-KI mice, presented on an animal-by-animal basis. **a**. (Top) Spike firing rate of *n* = 95 LEC_L2/3_ cells from an example young WT mouse #00364, shown in z-score during first 10 (T1), middle 10 (T3) and last 10 (T5) trials the session. (Middle) Time-resolved principal component analysis (PCA) trajectory of LEC_L2/3_ cell activity for each odor type (▷ cue-onset; ✩ cue-offset/delay-onset; □ delay-offset). (Bottom) Euclidean distance between odor types. **b**. Same as Extended Data Fig. 3a, but for example young APP-KI mouse #01517 (*n* = 97 cells). Trajectories for Odor-A and Odor-1 kept far apart through T1 to T5. **c**. Percentage of LEC_L2/3_ cells per mouse, classified by odor cue response in the first 10 trials (T1, top; *F*_4,40_ = 2.7, *p* = 0.043, responsive type x strain, ANOVA), middle 10 trials (T3, middle; *F*_4,40_ = 2.5, *p* = 0.057, responsive type x strain, ANOVA), and last 10 trials (T5, bottom; *F*_4,40_ = 8.0, *p* = 7.7e-5, responsive type x strain, ANOVA; *p* = 0.021 in 1 Odor response cell, *p* = 4.3e-3 in no response cell, two-sided Tukey post hoc test) (*n* = 5 mice per strain: young WT and young APP-KI mice). Data are presented as mean ± s.e.m. **d**. Mean Euclidean distance between PCA trajectories during 0.5 – 1.5 s after cue onset, averaged across young WT and young APP-KI mice (*n* = 5 each). Ninety-fifth percentile distance obtained from shuffled data denotes significant distance (red line).(A-1 mean distance, *p* = 0.048 in T5, two-sided unpaired t-test followed by false discovery rate (FDR) correction for multiple comparison; mean ± s.e.m.). **e**. Mean similarity index (SI) between Odor-A and -1 in young WT and young APP-KI mice across learning (mean ± s.e.m.; *n* = 5 each, *F*_1,56_ = 14.8, *p* = 3.1e-4, strain, ANOVA). Positive SI denotes similar representations between Odor-A and Odor-1, whereas negative SI denotes dissimilar representations.

**Extended Data Fig. 4:**
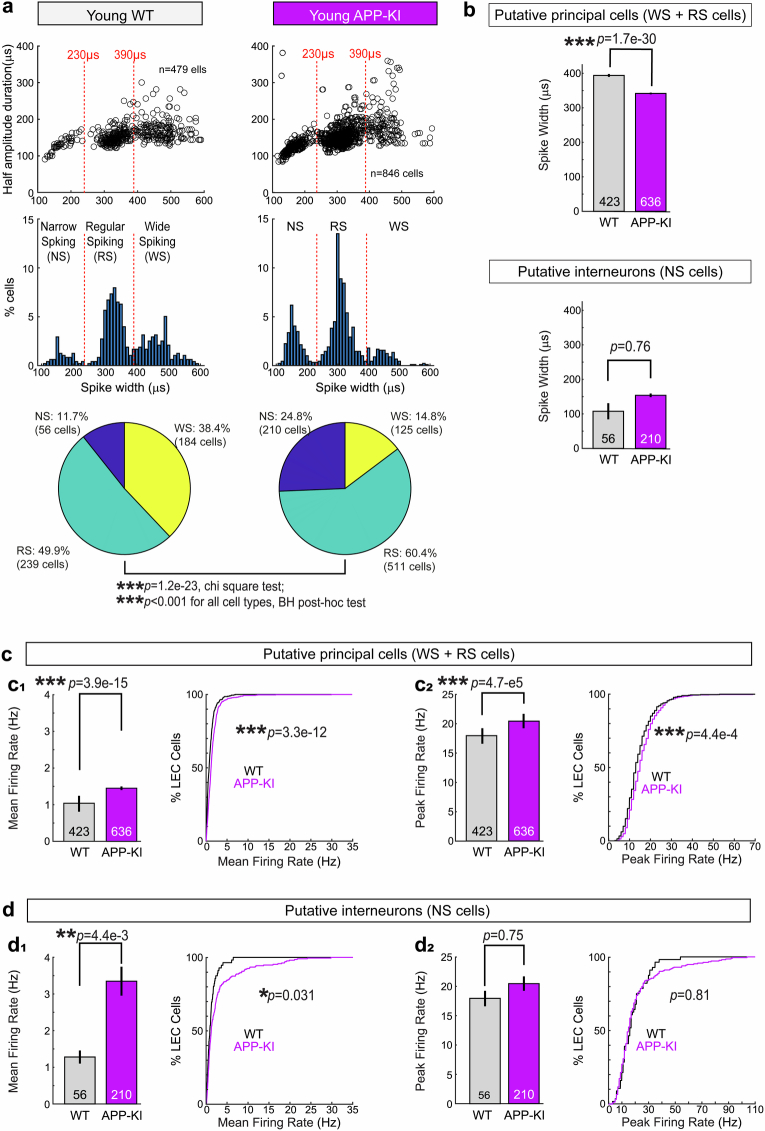
Spike properties of LEC_L2/3_ neurons in young WT and young APP-KI mice. **a**. Spike properties of LEC_L2/3_ neurons. Top, plot of individual neurons for half amplitude duration as a function of spike width in young WT (left) and young APP-KI (right) mice (*n* = 479 in young WT mice and *n* = 846 in young APP-KI mice). Dashed lines (230 and 390 µs) represent cut offs for classifying three neuron groups: narrow spiking (NS) cells, regular spiking (RS) cells and wide spiking (WS) cells. Middle, distributions of neurons as a function of spike width. Trimodal distribution of spike widths reveals three groups (NS cells, RS cells and WS cells). Bottom, percentage of NS, RS, and WS cells in young WT and young APP-KI mice. (*p* = 1.2e-23, Chi-squared test for comparing distributions; *p*_NS_ = 7.3e-9, *p*_RS_ = 1.0e-4, *p*_WS_ < 1.0e-5, Benjamini-Hochberg post-hoc test for comparing each cell type). **b**. Mean spike widths of putative principal cells (NS cells and WS cells) and putative interneurons (NS cells) between young WT and young APP-KI mice (*Z* = 11.47, *p* = 3.9e-15 for principal cells; *Z* = -0.29 *p* = 0.76 for interneurons; two-sided rank sum test). **c**. Mean firing rates of LEC_L2/3_ putative principal cells throughout the associative memory learning session. (**c1**) Left, mean firing rate (*Z* = -7.85, *p* = 3.9e-15, two-sided rank sum test). Right, a cumulative distribution plot (*p* = 3.3e-12, Kolmogorov-Smirnov test). (**c2**) Left, peak firing rate (*Z* = -4.06, *p* = 4.7e-5, two-sided rank sum test). Right, a cumulative distribution plot (*p* = 4.4e-4, Kolmogorov-Smirnov test). **d**. Same as Extended Data Fig. 4c, but for LEC_L2/3_ putative interneurons. Mean firing rates, *Z* = -2.84, *p* = 0.0044, two-sided rank sum test; *p* = 0.031, Kolmogorov-Smirnov test. No significant difference was observed for peak firing rates (*Z* = 0.31, *p* = 0.75, two-sided rank sum test; *p* = 0.81, Kolmogorov-Smirnov test). All data are presented as mean ± s.e.m.

**Extended Data Fig. 5:**
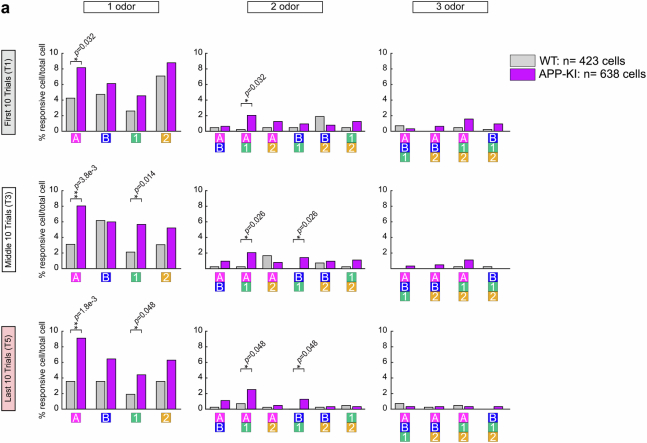
Cue response type of LEC_L2/3_ neurons in young WT and young APP-KI mice during learning. (**a**) LEC_L2/3_ principal neurons were classified for their cue response type during 0.5-1.5 s after cue onset and shown in percentage among all recorded neurons. Neurons were collected from both correct and error sessions. APP-KI mice had more Odor-A cells and Odor-A/1 cells compared to WT mice through T1-T5. Top, first 10 trials (T1); middle, 10 trials (T3); bottom, last 10 trials (T5) (*p*_T1_ = 5.5e-4, *p*_T3_ = 4.5e-7, *p*_T5_ = 6.4e-6, Chi-squared test for overall distributions; *p < 0.05, **p < 0.01, Benjamini-Hochberg post-hoc test for each responsive type; *n* = 423 cells from young WT vs. *n* = 636 cells from young APP-KI mice).

**Extended Data Fig. 6:**
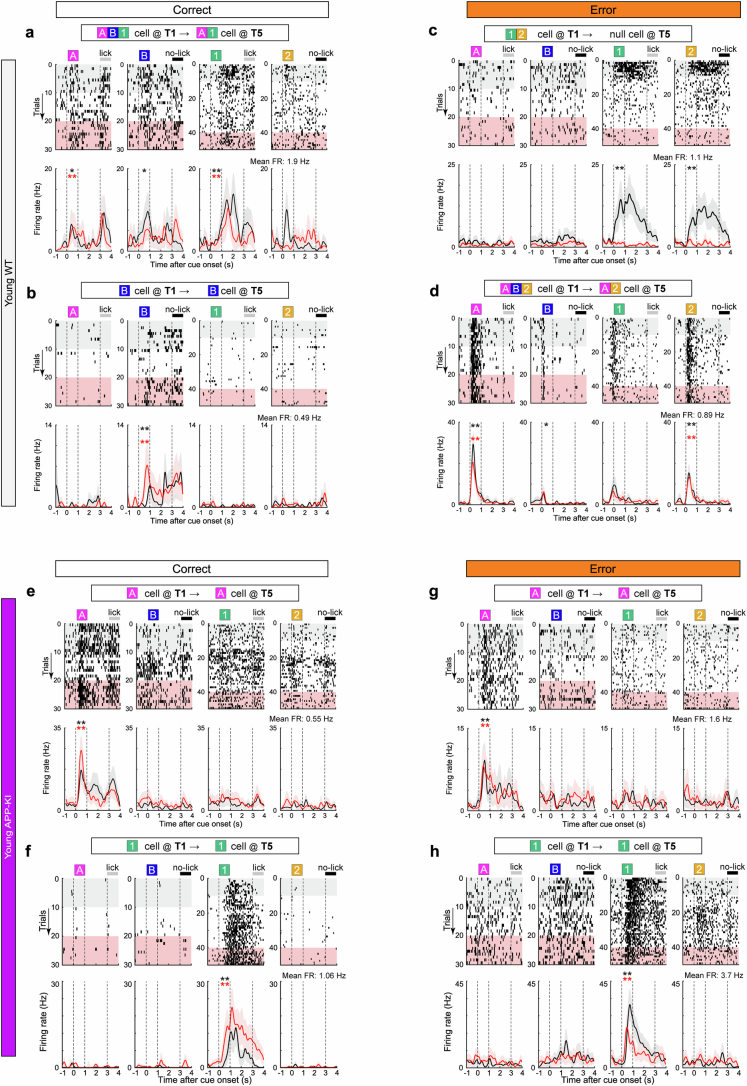
Selectivity profile of LEC_L2/3_ single neurons in young WT and young APP-KI mice during learning. Example LEC_L2/3_ neurons from young WT(**a-d**) and young APP-KI mice (**e-h**). Two neurons were shown each for correct and error sessions. APP-KI mice tended to have more Odor-A cells and Odor-1 cells responding to a single odor cue. *p < 0.05, **p < 0.01, ***p < 0.001 during cue-delay period; a, *p*_odorA-first_ = 0.015, *p*_odorA-last_ = 7.0e-3, *p*_odorB-first_ = 0.013, *p*_odor1-first_ = 2.0e-3, *p*_odor1-last_ = 1.3e-3; b, *p*_odorB-first_ = 9.8e-3, *p*_odorB-last_ = 2.0e-3; c, *p*_odor1-first_ = 3.9e-3, *p*_odor2-first_ = 2.0e-3; d, *p*_odorA-first_ = 5.9e-3, *p*_odorA-last_ = 2.0e-3, *p*_odorB-first_ = 0.037, *p*_odor2-first_ = 2.0e-3, *p*_odor2-last_ = 2.0e-3; e, *p*_odorA-first_ = 2.0e-3, *p*_odorA-last_ = 2.0e-3; f, *p*_odor1-first_ = 2.0e-3, *p*_odor1-last_ = 2.0e-3; g, *p*_odorA-first_ = 2.0e-3, *p*_odorA-last_ = 2.0e-3; h, *p*_odor1-first_ = 2.0e-3, *p*_odor1-last_ = 2.0e-3, two-sided Wilcoxon signed-rank test. Black trace, first 10 trials. Red trace, last 10 trials. Data are presented as mean ± s.e.m.

**Extended Data Fig. 7:**
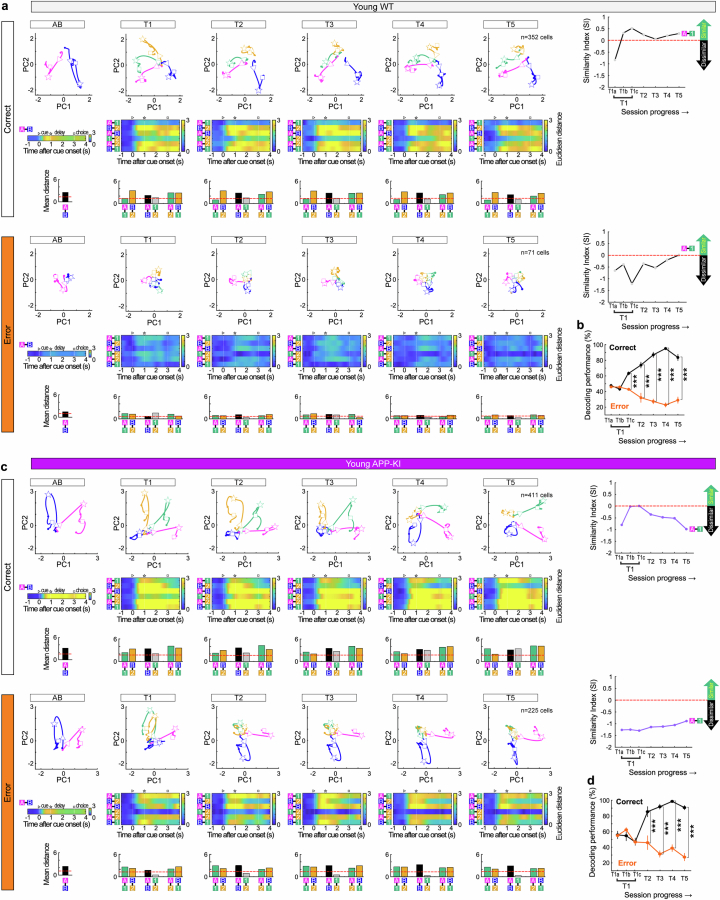
Principal component analyses and decoding analyses of LEC_L2/3_ neurons separated for correct and error sessions. **a**. PCA trajectories (left), Similarity Index (right top) from spike activity of LEC_L2/3_ population in young WT mice. T1 is subdivided into T1a (trials 1-3), T1b (trials 4-6) and T1c (trials 7-9). **b**. Decoding analysis where a support vector machine (SVM) was trained using activities from Odor-A vs Odor-B and tested for performance to discriminate between Odor-1 vs. Odor-2. Bootstrapping method (*n* = 10) was used to obtain results from resampled data. Decoding performance from correct sessions and error sessions were compared using ANOVA (mean ± s.e.m.; *F*_6,126_ = 37.83, *p* = 5.7e-26, session x time interaction, ANOVA; *p*_T1c_ = 3.5e-2, *p*_T2_ = 4.1e-17, *p*_T3_ < 1.0e-5, *p*_T4_ < 1.0e-5, *p*_T5_ < 1.0e-5 between correct session vs. error session, two-sided Tukey post hoc test). The decoding analysis showed that neural activity in correct sessions discriminated between Odor-1 vs. Odor-2, whereas activity in error session could not discriminate them in WT mice. **c-d**. Same as Extended Data Fig. 7a, but for spike activity in young APP-KI mice. Bootstrapping method (*n* = 10) was used to obtain results from resampled data. The decoding analysis from APP-KI mice showed that neural activity in correct sessions discriminated between Odor-1 vs. Odor-2, while activity in error session could not discriminate them also in APP-KI mice (mean ± s.e.m.; *F*_6,126_ = 21.59, *p* = 2.4e-17, session x time interaction, ANOVA; *p*_T2_ = 7.7e-7, *p*_T3_ = 1.7e-17, *p*_T4_ = 4.9e-17, *p*_T5_ = 1.9e-19 between correct session vs. error session, two-sided Tukey post hoc test; *n* = 10).

**Extended Data Fig. 8:**
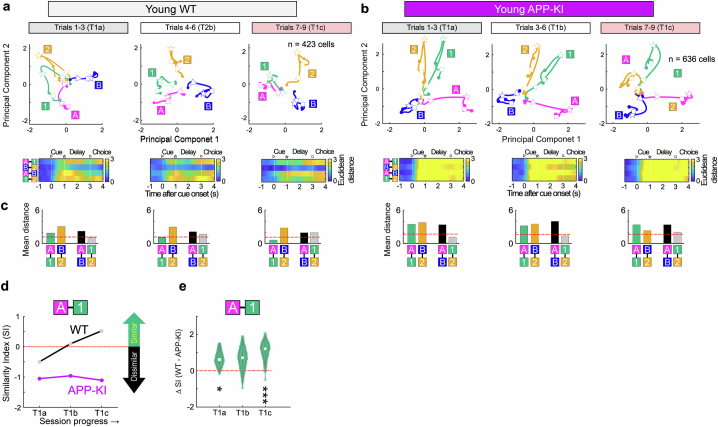
Detailed principal component analyses of LEC_L2/3_ neurons of young WT and young APP-KI mice within first 10 trials. To test the dynamics of spike representations in the early phase of the learning, PCA analysis were performed for Trial 1-3 (T1a), Trial 4-6 (T1b) and Trial 7-9 (T1c) within T1. **a**. (Top) Spike firing rate of *n* = 423 LEC_L2/3_ cells from young WT mice, shown in z-score during T1a, T1b and T1c. (Middle) Time-resolved principal component analysis (PCA) trajectory of LEC_L2/3_ cell activity for each odor type (⊳ cue-onset; ✩ cue-offset/delay-onset; ☐ delay-offset). (Bottom) Euclidean distance between odor types. **b**. Same as (a), but for young APP-KI mice (*n* = 636 cells). Trajectories for Odor-A and Odor-1 kept far apart through T1a to T1c. **c**. Mean Euclidean distance between PCA trajectories during 0.5 – 1.5 s after cue onset in young WT (left) and young APP-KI mice (right). Ninety-fifth percentile distance obtained from shuffled data denotes significant distance (red line). **d**. Similarity index (SI) between Odor-A and -1 in young WT and young APP-KI mice across learning. Positive SI denotes similar representations between Odor-A and Odor-1, whereas negative SI denotes dissimilar representations. **e**. Similarity Index (SI) of young WT and young APP-KI mice was compared using bootstrapping method (see Method). SI was calculated for 1000 bootstraps, then SIs for WT were subtracted by SIs for APP-KI. The subtraction confirms higher similarity in the representations between Odor-A and Odor-1 in WT mice compared to that in APP-KI mice (*p < 0.05, ***p < 0.001, *p*_T1a_ = 0.023, *p*_T1c_ = 8.7e-4; *n* = 1000 two-sided bootstrapping test).

**Extended Data Fig. 9:**
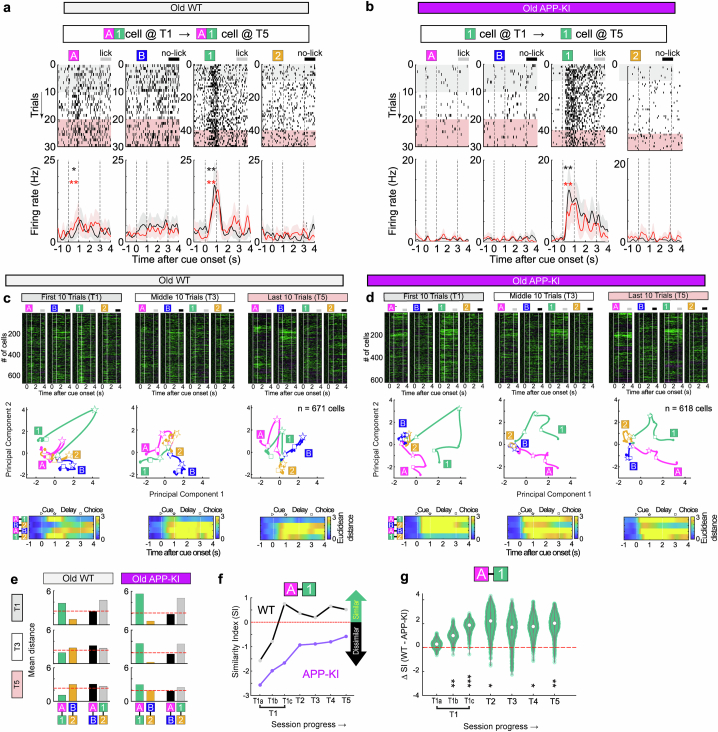
Disrupted cue-reward association of LEC_L2/3_ neurons in old APP-KI mice. **a**. An example LEC_L2/3_ cell from an old WT mouse showing constant firing for Odor-A and Odor-1. (mean ± s.e.m.; *p < 0.05, **p < 0.01 during cue-delay period, *p*_odorA-first_ = 0.027, *p*_odorA-last_ = 2.0e-3, *p*_odor1-first_ = 9.8e-3, *p*_odor1-last_ = 9.8e-3, two-sided Wilcoxon signed-rank test). Black trace, first 10 trials. Red trace, last 10 trials. mean ± s.e.m.; **p < 0.01 during cue-delay period compared to pre-cue period, *p*_odorA-first_ = 0.027, *p*_odorA-last_ = 2.0e-3, *p*_odor1-first_ = 9.8e-3, *p*_odor1-last_ = 9.8e-3, two-sided Wilcoxon signed-rank test). **b**. An example LEC_L2/3_ cell from an old APP-KI mouse exhibited constant firing only for Odor-1. (mean ± s.e.m.; *p < 0.05, **p < 0.01 during cue-delay period, *p*_odor1-first_ = 2.0e-3, *p*_odor1-last_ = 3.9e-3, two-sided Wilcoxon signed-rank test). **c**. (Top) Spike firing rate of n = 671 LEC_L2/3_ cells from old WT mice, shown in z-score during first 10 (T1), middle 10 (T3) and last 10 (T5) trials of the session. (Middle) Time-resolved PCA trajectories of LEC_L2/3_ cell activity for each odor type (⊳ cue-onset; ✩ cue-offset/delay-onset; ☐ delay-offset). (Bottom) Euclidean distance between odor types. **d**. Same as Extended Data Fig. 9c, but for old APP-KI mice (*n* = 618 cells). Trajectories for Odor-A and Odor-1 kept far apart through T1 to T5. **e**. Mean Euclidean distance between each odor type during 0.5 – 1.5 s after cue onset in old WT (left) and old APP-KI mice (right). Ninety-fifth percentile distance obtained from shuffled data denotes significant distance (red line). **f**. Similarity index (SI) between Odor-A and -1 in old WT and old APP-KI mice across learning. Positive SI denotes similarity of representations between Odor-A and Odor-1, whereas negative SI denotes dissimilarity. T1 is subdivided into T1a (trials 1-3), T1b (trials 4-6) and T1c (trials 7-9). **g**. Similarity Index (SI) of old WT and old APP-KI mice was compared using bootstrapping method. SI was calculated for 1000 bootstraps, then SIs for old WT were subtracted by SIs for old APP-KI. The subtraction confirms higher similarity in the representations between Odor-A and Odor-1 in old WT mice compared to that in old APP-KI mice (*p*_T1b_ = 5.9e-3, *p*_T1c_ = 7.1e-6, *p*_T1c_ = 8.7e-4, *p*_T2_ = 0.015, *p*_T4_ = 0.017, *p*_T5_ = 6.9e-3, *n* = 1000 two-sided bootstrapping test). Statistical significance is indicated as *p < 0.05, **p < 0.01, ***p < 0.001.

We next investigated memory-encoding spike activity of LEC neurons in brains with AD. We implanted recording drives with 64 electrodes targeting LEC_L2/3_ and recorded 479 and 846 neurons from *n* = 5 young WT and *n* = 5 young APP-KI mice, respectively (Fig. 2 and Extended Data Figs. 2 and 3). LEC_L2/3_ neurons in APP-KI mice showed decreasing spike widths compared to those of WT mice, suggesting alterations in spike generation mechanisms (Extended Data Fig. 4 and Supplementary Fig. 2). Consistent with Aβ-induced neuronal hyperactivity observed previously in the hippocampus^23,24^, the mean firing rates of putative LEC_L2/3_ principal neurons and interneurons were both higher in APP-KI mice than those of WT mice (Extended Data Fig. 4). An example WT neuron from a correct session (Fig. 2b) responded to prelearned rewarded Odor-A from the initial 10 trials (T1, black ^*^ in Fig. 2b; *P* < 0.01, rank sum test compared to precue period). This cell quickly acquired a response also to novel rewarded Odor-1, and these responses continued through the last 10 trials (T5, *P* < 0.01, red ^*^ in Fig. 2b). Thus, this neuron underwent a plastic change from an ‘Odor-A cell’ at T1 to an ‘Odor-A/1 cell’ at T5 during learning. We previously showed that these A/1 cells are the key neurons in the LEC for establishing the generalization of new rewarded cues (Odor-1) with prelearned rewarded cue (Odor-A)^16^. In contrast, an example neuron from young APP-KI mice (Fig. 2c) showed spike firing to both Odor-B and Odor-1 (Odor-B/1 cell), which erroneously generalizes prelearned punished Odor-B with novel rewarded Odor-1. We analyzed response properties of the LEC_L2/3_ population. While 16% of LEC_L2/3_ neurons fired to at least one odor cue at T5 in WT mice, an excessive 34% of neurons exhibited cue responses in APP-KI mice (Fig. 2d, ***χ***^*2*^ = 2.29, *P* = 3.6 × 10^−9^, chi-squared test; *P* < 0.01 for 1-odor and 2-odor responsive cells, Benjamini–Hochberg post hoc test). Among these cue-responsive neurons in APP-KI mice, the percentage of Odor-A responsive cells was higher in APP-KI mice compared to those in WT mice (Extended Data Fig. 5 and Supplementary Fig. 3; see Extended Data Fig. 6 for cell responses). Population representations were then assessed using principal component analysis^16^ (PCA) (Fig. 2e,f and Supplementary Fig. 4). For this analysis, we used all sessions including both correct and error sessions to assess overall alteration (see Extended Data Fig. 7 for separate PCA for correct and error sessions). In WT mice, trajectories of Odor-A and Odor-1 were distant in principal component space in T1, but became close after T3 (Fig. 2e). These similar representations between Odor-A and Odor-1 in the late phase of the learning are the characteristic property for generalizing rewarded cues found previously in the LEC^16^. In contrast, in APP-KI mice, trajectories for the individual four cues remained far apart throughout the session, separating representations of Odor-A from Odor-1 (Fig. 2f). We measured Euclidean distances between trajectories to assess similarity of spike representations between cue types (Fig. 2g). The 95th percentile distance obtained from shuffled data was used as a threshold for statistical significance^16^ (red line, Fig. 2g). In WT mice, A-1 distance was slightly larger than the shuffle at T1, but became smaller than the shuffle through T2–T5, indicating generalized representations of rewarded cues during learning. We further subdivided T1 into trials 1–3 (T1a), 4–6 (T1b) and 7–9 (T1c) and observed clear decrease of A-1 distance from T1a to T1c (Extended Data Fig. 8). However, A-1 distance in APP-KI mice remained above shuffle, indicating that Odors-A and -1 representations were not generalized together in APP-KI mice. This dissimilarity is apparent using a Similarity Index (SI)^16^ (Methods and Fig. 2h). A bootstrapping analysis confirmed the decreased A-1 similarity in APP-KI mice compared to WT mice throughout the session (*P* = 0.023 at T1a, *P* = 0.072 at T1b, *P* = 8.7 × 10^−4^ at T1c, *P* = 6.6 × 10^−7^ at T2, *P* = 1.7 × 10^−5^ at T3, *P* = 8.7 × 10^−6^ at T4, *P* = 1.1 × 10^−3^ at T5, maximum likelihood estimation, Fig. 2i). Similar results were obtained when data were separated and analyzed per animal (Fig. 2j–l, Extended Data Fig. 3 and Supplementary Figs. 2 and 3). In old age, generalization of Odors-A and -1 was observed through T2–T5 in WT mice, whereas representations for these two cues were again kept separated throughout the session in APP-KI mice (Extended Data Fig. 9 and Supplementary Fig. 5). Together, these data demonstrate that the spike representation for generalizing Odor-A and Odor-1, observed in healthy WT mice, was disrupted in APP-KI mice from a young age.

### Dysfunction of lateral entorhinal cortex dopamine activity

**Fig. 3:**
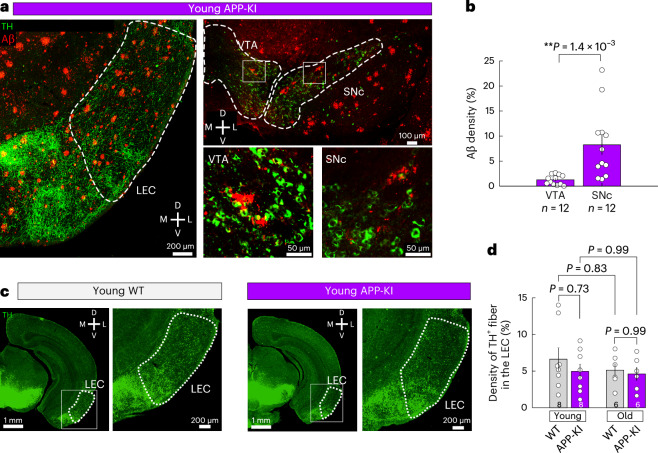
Spared dopamine axons in the LEC of APP-KI mice. **a**, Coronal section showing anti-TH and anti-Aβ immunostaining of young APP-KI mice. Left: LEC; right: VTA and SNc. **b**, Aβ density is higher in the SNc than VTA (*z* = −3.2, ^**^P = 1.4 × 10^−3^, two-sided rank sum test; *n* = 12 mice). **c**, Coronal section showing anti-TH immunostaining. Left: young WT mice; right: young APP-Ki mice. **d**, Quantification of TH^+^ fiber density in the LEC of WT and APP-KI mice. *P* values for post hoc Tukey test are shown (*n* = 8 young WT mice, *n* = 8 young APP-KI mice, *n* = 6 old WT mice, *n* = 6 old APP-KI mice). All post hoc *P* values, including those not shown, were above 0.05 (*F*_1,24_ = 0.22, *P* = 0.22, strain × age interaction, ANOVA; *P* > 0.05, two-sided Tukey post hoc test). All data are presented as mean ± s.e.m.

**Extended Data Fig. 10:**
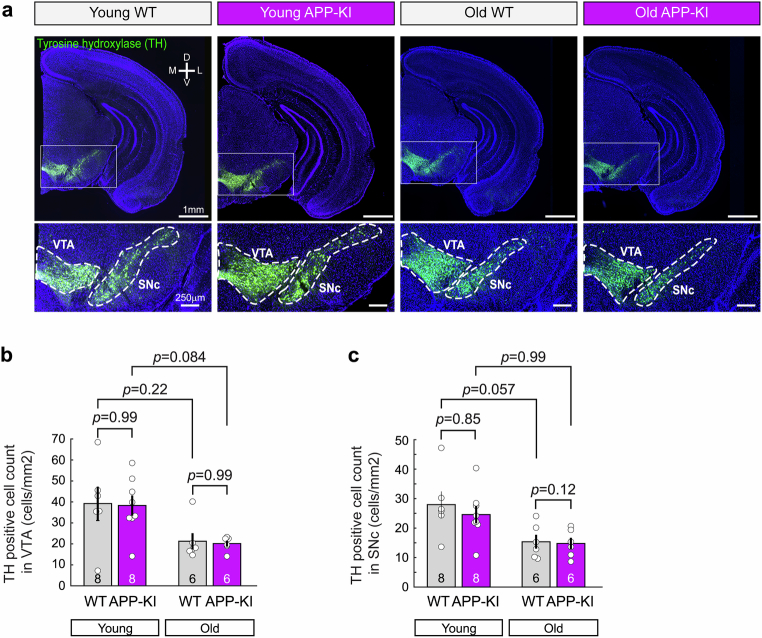
Density of dopaminergic cell bodies in the VTA and SNc did not differ between WT and APP-KI mice. **a**. (From left to right) Coronal sections of the VTA and SNc with anti-Tyrosine hydroxylase immunostaining for young WT, young APP-KI, old WT, and old APP-KI mice. D, dorsal, V, ventral, M, medial, L, lateral. **b**. Number of TH positive cells in the VTA of WT and APP-KI mice (*F*_1,22_ = 0.08, *p* = 0.78, strain x age interaction, ANOVA). P-values for post-hoc Tukey test are shown in the figure (*n* = 4 each from WT mice and APP-KI mice). All two-sided Tukey post hoc test p-values, including those not shown in the figure, were above 0.05. **c**. Same as Extended Data Fig. 10b but for number of TH positive cells in the SNc. (*F*_1,22_ = 0.20, *p* = 0.65, strain x age interaction, ANOVA). All two-sided Tukey post hoc test p-values, including those not shown in the figure, were above 0.05. All data are presented as mean ± s.e.m.

In healthy brains, the spike encoding of LEC neurons for generalizing rewarded cues is controlled by dopamine inputs from the VTA and SNc to the LEC^16^. Our previous optogenetic inhibition of LEC dopamine in healthy mice led to impaired associative memory encoding in LEC_L2a_ neurons as well as impaired associative learning of mice^16^, similar to the results obtained in APP-KI mice. Therefore, we investigated further LEC dopamine activity in APP-KI mice. Since APP-KI mice already exhibit disruption of LEC spike activity from the young age of 3–6 months, we focused our analyses on this early stage. Aβ accumulation emerged from 4 months in midbrain regions including the VTA and SNc (Fig. 1 and Extended Data Fig. 1). Co-staining with tyrosine hydroxylase (TH) showed Aβ plaques partially overlapping with cell bodies of dopamine neurons in the VTA and SNc (Fig. 3a). The density of Aβ plaques was higher in the SNc than that in the VTA (*Z* = −3.2, *P* = 1.4 × 10^−3^, rank sum test; Fig. 3b). In the LEC, dopamine fibers also partially overlap with Aβ plaques (Fig. 3a). These data suggest possible damage of dopamine neurons caused by Aβ plaques. To assess whether dopamine axons degenerate in the LEC, we measured the density of TH^+^ axons in the LEC (Fig. 3c,d). Although there was a trend of decrease in the density of TH^+^ axons of APP-KI mice, the effect was not significant across strain nor age (Fig. 3d, *F*_1,24_ = 0.79, *P* = 0.38 for strain; *F*_1,24_ = 0.57, *P* = 0.46 for age, *F*_1,24_ = 0.22, *P* = 0.22 for strain × age interaction; two-way ANOVA). We also measured the density of dopaminergic neurons in the VTA and SNc, finding no significant alteration between APP-KI and WT mice (Extended Data Fig. 10).

**Fig. 4:**
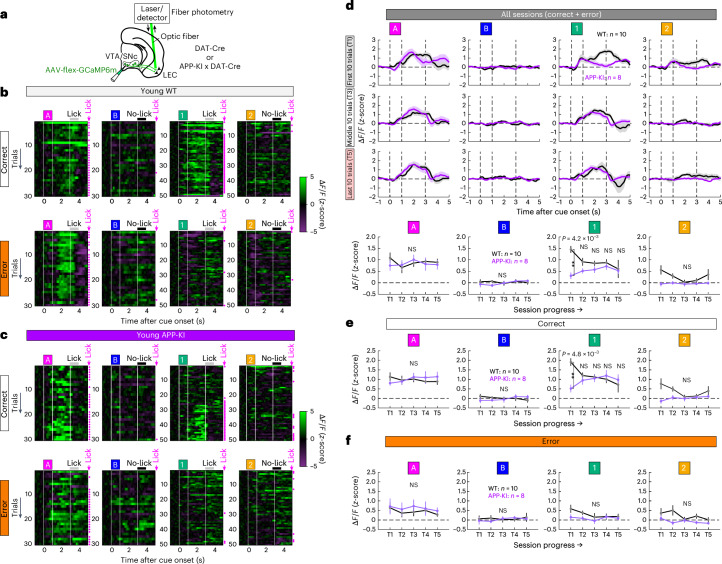
Dysfunction of LEC dopamine in young APP-KI mice. **a**, Fiber photometry recording of calcium signals from dopamine fibers in the LEC. **b**, Example GCaMP signals from LEC dopamine fibers shown in *z*-scored Δ*F*/*F*, from a young WT mouse. Top: correct session; bottom: error session. **c**, As in Fig. 4b but for young APP-KI mice. Top: correct session; bottom: error session. **d**, Top: GCaMP signals during first 10 trials (T1), middle 10 trials (T3) and last 10 trials (T5). Photometry signals from both correct and error sessions were averaged for WT and APP-KI mice (*n* = 10 and *n* = 8 hemispheres from WT and APP-KI mice, respectively). Bottom: mean GCaMP signals during 1–4 s after cue onset in APP-KI mice showed decreasing signals on Odor-1 trials compared to WT mice (*F*_4,80_ = 2.5, *P* = 0.047, strain × time interaction, ANOVA; *P*_T1_ = 4.2 × 10^−3^, two-sided Tukey post hoc test; *n* = 10 and *n* = 8 hemispheres from WT and APP-KI mice, respectively). **e**, Mean GCaMP signals from correct sessions (Odor-1; *F*_4,80_ = 3.72, *P* = 7.8 × 10^−3^, strain × time interaction, ANOVA; *P*_T1_ = 4.8 × 10^−3^, two-sided Tukey post hoc test; *n* = 10 and *n* = 8 hemispheres from WT and APP-KI mice, respectively). **f**, As in Fig. 4e but for error sessions. There is no significant difference between young WT and young APP-KI (NS *P* > 0.05, ANOVA; *n* = 10 and *n* = 8 hemispheres from WT and APP-KI mice, respectively). All data are presented as mean ± s.e.m. Statistics details are available in Supplementary Table 1.

Since LEC dopamine fibers did not show significant morphological degeneration in APP-KI mice, we examined functional activity of LEC dopamine axons using fiber photometry^16^. Adeno-associated virus (AAV) expressing calcium activity indicator GCaMP in a Cre-dependent manner (AAV-flex-GCaMP6m) was injected in the VTA or SNc of young APP-KI mice crossed with DAT-Cre (APP-KI × DAT-Cre), and photometry fibers were implanted in the LEC (Fig. 4a). When control DAT-Cre mice successfully learned the association (correct sessions), LEC dopamine supplied reward expectation signals during rewarded trials, as observed previously^16^ (Fig. 4b, top). At T1, dopamine was released primarily during delay periods of prelearned rewarded Odor-A (mean *z*-scored GCaMP signal, 1.1 ± 0.24; *Z* = −2.7, *P* = 6.9 × 10^−3^, rank sum test compared to precue period). The signal also emerged for novel Odor-1 (1.8 ± 0.2; *Z* = −2.8, *P* = 5.1 × 10^−3^) and Odor-2 cues (0.76 ± 0.24; *Z* = −2.27, *P* = 0.028). As learning progressed, dopamine signals disappeared for Odor-2, while signals remained for rewarded Odor-A and Odor-1 through T5. These dopamine signals were absent in error sessions, where WT mice did not learn associations of novel odors (Fig. 4b, bottom). These results suggest a correlation between dopamine activity during Odor-1 and successful learning, as shown previously^16^. In APP-KI mice, in contrast, signals for Odor-1 emerged in a delayed manner from T3 (Fig. 4c, top), while dopamine signals for Odor-A remained intact when mice learned the association (correct session). The delayed emergence of Odor-1 dopamine signals correlated well with slow learning of licking to Odor-1 in APP-KI mice (*r*(19) = 0.7810, *P* = 2.5 × 10^−5^, Pearson correlation, Supplementary Fig. 6), implying that slow emergence of LEC dopamine signals causes delayed learning in APP-KI mice. During the remaining 39% of sessions where APP-KI mice did not reach the 80% criterion (error sessions), dopamine signals for Odor-1 were completely absent, while the signals for Odor-A again remained present (Fig. 4c, bottom). When the overall dopamine levels across all sessions were compared between WT and APP-KI mice, dopamine activity was significantly decreased in APP-KI mice during Odor-1 trials at T1 (*F*_4,80_ = 2.5, *P* = 0.047, strain × time interaction, ANOVA; *P* = 4.2 × 10^−3^ in T1, post hoc Tukey test; Fig. 4d). Dopamine was also decreased in APP-KI mice during Odor-1 at T1 in correct sessions (Fig. 4e, Odor-1, *F*_4,80_ = 3.7, *P* = 7.8 × 10^−3^, ANOVA; *P* = 4.8 × 10^−3^, post hoc Tukey test; Odor-2, *F*_4,80_ = 2.1, *P* = 0.093 in Odor-2, strain × time interaction, ANOVA). No difference was observed in error sessions (Fig. 4f). To directly compare the degree of dopamine decrease across odor types, we compared the difference of dopamine activity between APP-KI and WT mice (Supplementary Fig. 7). The difference for Odor-1 was significantly larger than that for Odor-A or Odor-B, suggesting specific decrease of dopamine for Odor-1 (*P* < 0.05, ANOVA followed by Tukey post hoc test). Odor-2 did not show significant difference compared to other odor types. Taken together, these data demonstrate that, while the LEC dopamine fiber structure was relatively intact, LEC dopamine activity for novel cues, especially for novel rewarded Odor-1, became dysfunctional from a young age in APP-KI mice.

### Rescued memory by dopamine reactivation

**Fig. 5:**
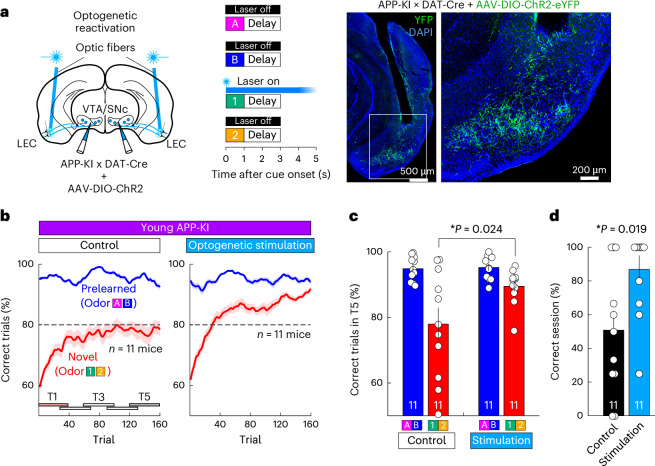
Optogenetic reactivation of LEC dopamine rescued associative memory of APP-KI mice. **a**, Left: optogenetic stimulation of dopamine fibers in the LEC during Odor-1 trials. Right: representative section from *n* = 11 stimulated mice showing an optic fiber track and ChR2-expressing dopamine fibers in the LEC. **b**, Correct trial rate for prelearned odors (A/B, blue) and novel odors (1/2, red) in control no-stimulation sessions (left) and optogenetic stimulation sessions (right), obtained from *n* = 11 young APP-KI mice. **c**, Percentage of correct trials in T5 of control sessions and optogenetic stimulation sessions (*F*_1,40_ = 4.17, *P* = 0.047, odor × session interaction, ANOVA; *P*_odor1/2-Ctrvsodor1/2-Stim_ = 0.024, two-sided Tukey post hoc test; *n* = 11 young APP-KI mice). **d**, Percentage of sessions where mice correctly learned new association (*t*(20) = −2.55, *P* = 0.019, two-sided unpaired *t*-test). All data are presented as mean ± s.e.m.

Having established that LEC dopamine is diminished in young APP-KI mice, we finally asked whether the reactivation of LEC dopamine could rescue associative memory in APP-KI mice. First, to directly reactivate LEC dopamine, we optogenetically stimulated LEC dopamine fibers while young APP-KI mice performed the associative memory task (Fig. 5a). Channelrhodopsin-2 (ChR2) was expressed in dopamine fibers of APP-KI × DAT-Cre mice using AAV-DIO-ChR2 injected in the VTA or SNc, and optic fibers for laser stimulation were bilaterally implanted above the LEC. Since LEC dopamine was diminished largely during novel rewarded Odor-1 trials (Fig. 4 and Supplementary Fig. 7), we stimulated LEC dopamine fibers only on Odor-1 trials (Fig. 5a). When LEC dopamine axons were stimulated, APP-KI mice showed significantly enhanced performance for the acquisition of novel odor associations compared to no-stimulation control sessions (stimulation: 95.2 ± 1.6% versus control: 77.9 ± 4.9% at T5; *F*_1,40_ = 4.17, *P* = 0.045, odor × stimulation interaction, ANOVA; *P* = 0.024, post hoc Tukey test; *n* = 11 young APP-KI mice; Fig. 5b,c and Supplementary Fig. 8a). The percentage of total correct sessions significantly increased with stimulation (stimulation 86.9 ± 8.2% versus control 50.1 ± 11.4%; *P* = 0.019, unpaired *t*-test; Fig. 5d). No rescuing effect was observed when dopamine axons were stimulated during all four odor types, supporting that the deficit of dopamine during Odor-1 is critically involved in learning impairment (Supplementary Fig. 9).

**Fig. 6:**
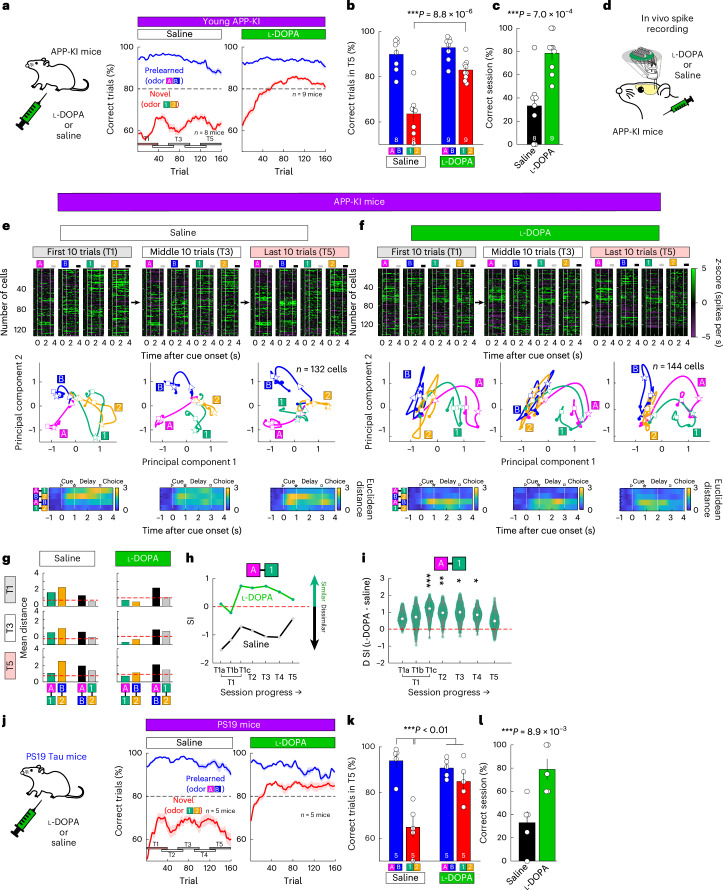
L-DOPA treatment restored both associative memory of APP-KI mice and associative memory encoding of LEC_L2/3_ neurons. **a**, Correct trial rate for prelearned odors (A/B, blue) and novel odors (1/2, red) in saline-injected young APP-KI mice (*n* = 8, left) and L-DOPA-injected young APP-KI mice (*n* = 9, right). Data are presented as mean ± s.e.m. **b**, Percentage of correct trials in T5 in saline- and L-DOPA-injected young APP-KI mice (mean ± s.e.m.; *F*_1,30_ = 3.6, *P* = 0.029, odor × session interaction, ANOVA; *p*_odor1/2-Salvsodor1/2-L-DOPA_ = 1.1 x 10^−3^, two-sided Tukey post hoc test; *n* = 8 saline mice and *n* = 9 L-DOPA mice). **c**, Percentage of sessions where mice correctly learned new association (mean ± s.e.m.; *t*(15) = -4.25, *P* = 7.0 × 10^−4^, two-sided unpaired *t*-test). **d**, Schematic diagram of the in vivo spike recording experiment during L-DOPA treatment. **e**, Top: spike firing rate of *n* = 132 LEC_L2/3_ cells from saline-injected young APP-KI mice, shown in *z*-score during first 10 (T1), middle 10 (T3) and last 10 (T5) trials the session. Middle: time-resolved PCA trajectories of LEC_L2/3_ cell activity for each odor type (▷, cue onset; ✩, cue offset/delay onset; □, delay offset). Bottom: Euclidean distance between odor types. **f**, As in Fig. 6e, but for L-DOPA-injected young APP-KI mice (*n* = 144 cells). Trajectories for Odor-A and Odor-1 remained separated through T1 to T5. **g**, Mean Euclidean distance between PCA trajectories during 0.5–1.5 s after cue onset in saline- (left) and L-DOPA-injected young APP-KI mice (right). The 95th percentile distance obtained from shuffled data denotes significant distance (red line). **h**, SI between Odor-A and Odor-1 in saline- and L-DOPA-injected young APP-KI mice across learning. Positive SI denotes similar representations between Odor-A and Odor-1, whereas negative SI denotes dissimilar representations. T1 is subdivided into T1a (trials 1–3), T1b (trials 4–6) and T1c (trials 7–9). **i**, SI of saline and L-DOPA was compared using bootstrapping method (Methods). SI was calculated for 1,000 bootstraps, then SIs from L-dopa-injected mice were subtracted by SIs from saline-injected mice. The subtraction confirms higher similarity in the representations between Odor-A and Odor-1 in L-dopa treatment compared to that in saline control (*P*_T1c_ = 9.1 × 10^−5^, *P*_T2_ = 4.8 × 10^−3^, *P*_T3_ = 0.011, *P*_T4_ = 0.041, *n* = 1,000 two-sided bootstrapping test). **j**, Schema of L-DOPA treatment in young PS19 mice. Correct trial rate for prelearned odors (A/B, blue) and novel odors (1/2, red) in saline-injected (left) and L-DOPA-injected (right) mice, obtained from *n* = 5 saline-injected mice and *n* = 5 L-DOPA injected mice. **k**, Percentage of correct trials in T5 of saline- and L-DOPA-injected mice (*F*_1,16_ = 9.88, *P* = 9.1 × 10^−6^, odor × drug interaction, ANOVA; *P*_odorA/B-Salvsodor1/2-Sal_ = 1.2 × 10^−4^, *P*_odor1/2-SalvsodorA/B-ldopa_ = 1.2 × 10^−4^, *P*_odor1/2-Salvsodor1/2-ldopa_ = 4.7 × 10^−3^, two-sided Tukey post hoc test). **l**, Percentage of sessions where mice correctly learned new association (*t*(8) = −3.18, ^***^P = 8.9 × 10^−^^3^, two-sided unpaired *t-*test). Statistics details are available in Supplementary Table 1. Statistical significance is indicated as ^*^P < 0.05, ^**^P < 0.01, ^***^P < 0.001.

We then tested L-DOPA, a precursor of dopamine used for the treatment of patients with Parkinson’s disease^25^. Young APP-KI mice injected intraperitoneally with L-DOPA recovered their associative memory up to the level of WT mice, with enhanced performance compared to saline-injected groups (L-DOPA groups 82.6 ± 1.7% versus saline group 67.1 ± 4.2% at T5; *F*_1,30_ = 5.2, *P* = 0.029, odor × drug interaction, ANOVA; *P* = 1.1 × 10^−3^, post hoc Tukey test; *n* = 9 L-DOPA mice, *n* = 8 saline mice; Fig. 6a–c and Supplementary Fig. 8b). The percentage of total correct sessions significantly increased with L-DOPA treatment (L-DOPA 78.5 ± 5.6%, saline 33.1 ± 9.4%; *P* = 7.0 × 10^−4^, unpaired *t*-test; Fig. 6c).

To test if L-DOPA treatment modulates LEC neuronal representations, we recorded *n* = 144 and 132 LEC_L2/3_ neurons from APP-KI mice, injected with either L-DOPA or control saline, respectively (*n* = 4 L-DOPA mice and *n* = 4 saline mice). Saline-injected APP-KI mice showed separated trajectories for prelearned rewarded Odor-A and novel rewarded Odor-1 (Fig. 6e,g), resulting in negative SI values (Fig. 6h). By contrast, in L-DOPA treated APP-KI mice, trajectories of Odor-A and Odor-1 become significantly close compared to shuffle (Fig. 6f,g). As a result, SI values were above 0 from trials 7–9 (T1c) to T5. These data indicate that L-DOPA treatment rescued associative memory, by involving the restoration of associative memory encoding of LEC_L2/3_ neurons.

L-DOPA treatment also had a rescuing effect on associative memory in PS19 mice, a widely used transgenic tau model of AD (L-DOPA groups 84.8 ± 3.8% versus saline group 64.8 ± 4.6% at T5; *F*_2,16_ = 10.9, *P* = 4.4 × 10^−3^, ANOVA; *P* = 4.7 × 10^−3^, post hoc Tukey test; *n* = 5 L-DOPA mice versus *n* = 5 saline mice; Fig. 6j–l and Supplementary Fig. 10). No effect was observed in L-DOPA-treated control WT mice (*F*_1,4_ = 0.011, *P* = 0.91, odor × drug interaction, ANOVA; *n* = 4 L-DOPA mice and *n* = 4 saline mice, Supplementary Fig. 11).

Together, these results indicate that LEC dopamine dysfunction underlies the associative memory impairment in young APP-KI mice. Our results further suggest that reactivation of LEC dopamine has the potential to rescue associative memory formation in brains impacted with AD.

## Discussion

Although the role of the entorhinal cortex, especially the LEC, has been long hypothesized in AD pathogenesis, it has remained unclear why this region suffers selective early dysfunction. Here we obtained solid circuit-level evidence that dopamine inputs from the VTA or SNc to the LEC become dysfunctional in the early stage of pathophysiology, causing associative memory impairments. While the pathogenic role of dopamine neurons in Parkinson’s disease has been well documented, its role in AD has remained poorly understood due to obscurity of dopamine projections to the entorhinal–hippocampal memory circuit. Past research has reported decreased dopamine and dopamine receptor expressions in the hippocampus of patients with AD^26,27^. However, the hippocampus receives limited VTA and SNc axons, rather receiving axons from the locus coeruleus that co-release dopamine and norepinephrine^28,29^. Our recent finding paved a way to investigate the role of dopamine in the entorhinal cortex^16^. We propose that the selective functional vulnerability of LEC in AD is attributed, at least partially, to the dopamine projection pattern specific to the LEC within the entorhinal–hippocampal circuit. Dopamine fibers in the entorhinal cortex have been identified not only in rodents^30,31^ but also in humans^32–36^, while, so far, no research to assess dopamine in the entorhinal cortex has been performed in patients with AD, to our knowledge. Our results highlight the need to investigate dopamine functions in the entorhinal cortex of human brains with AD.

In young APP-KI mice at 4 months, the density of LEC dopamine fibers was comparable to that of age-matched WT mice, suggesting limited axonal degeneration at the early AD stage (Fig. 3). However, dopamine signals for novel rewarded Odor-1 were significantly impaired in APP-KI mice, while signals for familiar rewarded Odor-A were relatively intact. These results suggest that, in young APP-KI mice, dopamine reserves are available at synaptic terminals in the LEC, yet fail to be released for novel rewarded Odor-1 cues. Our result of optogenetically-rescued associative memory also supports spared dopamine reserves in APP-KI mice. The decreased dopamine release for novel rewarded Odor-1 impaired memory encoding of LEC neurons, presumably due to the lack of dopamine-dependent neuronal plasticity^37,38^. Future work is required to decipher the cellular mechanism of how Aβ accumulation leads to LEC dopamine dysfunction specific for novel cues.

Aβ accumulation may also have impaired intrinsic spike mechanisms in the LEC circuit. The hyperactivity of both LEC_L2/3_ principal neurons and interneurons, observed irrespective of task engagements, might affect the generalizing function of LEC_L2/3_ neurons between Odor-A and Odor-1. Even with such alterations of LEC_L2/3_ neurons, the reactivation of LEC dopamine fibers could improve associative memory formation of APP-KI mice (Fig. 5). Stimulation of dopamine release using L-DOPA rescued the generalization of reward cues in LEC_L2/3_ neurons and associative memory (Fig. 6). Although optogenetic stimulation is not extendable to the treatment of patients with AD, L-DOPA treatment is widely used to increase global dopamine levels in patients with Parkinson’s disease. Our results raise the possibility that L-DOPA could also relieve memory impairments in early-stage AD symptoms. The mechanistic understanding of LEC dopamine dysfunction is expected to open a pathway to a future treatment for ameliorating memory functions in patients with AD.

## Methods

### Subjects

All procedures were conducted in accordance with the guidelines of the National Institutes of Health and approved by the Institutional Animal Care and Use Committee at the University of California. Mice were maintained in standard housing conditions on a reversed 12 h dark/light cycle, at temperatures of 20–24 °C, humidity of 30–40% and with food and water provided ad libitum. All experiments were conducted during the dark phase. Mouse lines and their received procedure are summarized in Supplementary Tables 2 and 4.

We used homozygous APP-KI mice (APP^NL-G-F^/APP^NL-G-F^, RIKEN BioResouce Research Center, RBRC06344) and a C57BL/6 J mouse (Jackson, 000664). Animals aged 3–6 months were used as young groups, and those aged 7–11 months were used for old groups. Mice at 12–13 months were additionally used for histological analysis. Similar number of females and males were included and used in each group. The number of animal sexes used is shown in Supplementary Table 2. All studies were performed in blind manners so that experimenters performed experiments without the knowledge of animal strains and ages. All animals had either C57BL/6 background or were backcrossed to C57BL/6 for at least seven generations. They were housed individually with an exercise wheel following their first procedure. Medications and appropriate treatments were applied across 1 to 2 weeks for recovery. If animals died before the postexperiment histological analysis, their recording positions could not be validated and therefore were removed from the data analysis.

### Electrode, drive and optic fiber preparation

#### Tetrode drive

Custom-built 64-channel drives containing bundled tetrodes were used for recording as previously described^16^.

#### Optic fibers

For optogenetic inhibition experiments, optic fibers of 400 µm diameter and 10–13 mm length (Thorlabs) were used.

### Surgery

All surgical procedures were performed as previously described^16^. Mice received postoperative injections of flunixin and enrofloxacin daily as needed.

#### Injections

For injections targeting the VTA and SNc, a 1-mm craniotomy was centered at AP 3.15 mm, ML 0.6/1.2 mm from bregma. The micropipette was lowered to a depth of 4,100 or 4,000 µm from the brain surface. Viruses were manually injected at approximately 0.1 µl min^−1^ using a hydraulic manipulator (MO-10, Narishige). Adeno-associated viruses (AAVs) used in the study are summarized in Supplementary Table 4.

#### Tetrode drive and optic fiber implantations

After allowing at least one week recovery from injection surgeries, mice were implanted with tetrode drives or optic fibers as previously described^16^. Briefly, a custom titanium head-plate was affixed to the skull, then craniotomies were drilled at the LEC coordinates (AP 3.5 mm, ML 3.5 mm from bregma, depth 3,300–3,500 μm from the brain surface, 10° lateral). For drive implants, reference wire was implanted on the cerebellar surface. Drives or optic fibers were secured to the skull with dental acrylic and protected with foil-lined conical shields.

### Training procedures

Behavioral training was performed as previously described^16^. Briefly, mice were water-deprived to 85% of baseline following postoperative recovery. Mice were habituated to head fixation inside a custom sound-proof chamber for 1–4 days. Licks were detected with an infrared emitter and sensor positioned at the sucrose or quinine lick spout. Tasks were automated with custom LabView scripts on a laptop connected to a DAQ port (National Instruments). Mice were trained to lick actively for sucrose in response to an LED cue following a nonodorized air puff (1–5 days). They were then trained to withhold licking during the 2 s delay period between air and LED cue (1–5 days). For prelearning of the go and no-go task, mice learned to lick for sucrose in response to Go Odor-A (isoamyl acetate) and withhold licking to No-Go Odor-B (α-pinene) to avoid quinine. Prelearning typically took 5–10 days.

#### Associative learning

A ~20-trial session with random trials of only Odor-A and Odor-B was tested first (AB-only session). Immediately afterwards, two novel odors in addition to Odor-A and Odor-B were presented in random order every 18–22 s (AB12 session). One of the novel odors was associated with lick, and the other with no-lick. Mice learned this new association by trial and error. Novel odor pairs were never repeated for an individual mouse. The lick-associated odors (that is, Odor-C, -E and -G) were collectively termed as Odor-1, and the no-lick-associated odors (that is, Odor-D, -F and -H) were termed as Odor-2. Odor-A and Odor-B were randomly delivered with 20% emergence rate each, whereas Odor-1 and Odor-2 were delivered with 30% emergence rate each. Licking during the delay period automatically aborted the trial. Odors used in the study are summarized in Supplementary Table 3.

### Data collection

#### Spike recording

Spiked data was acquired as previously described^16^. Tetrodes were advanced 40–80 µm to record a different group of cells in each session of a same type (either control or inhibition session) at the same recording position, assuring that identical cells were not double sampled. We typically advanced tetrodes after the recording of one session.

#### Optogenetic stimulation

Laser (473 nm, 20–25 mW) was only held on from the Odor-1 cue onset for 5 s, followed by a linear taper of 10 s on each trial. For control experiments, we used no-laser sessions in the same mice used for stimulation, where disconnected laser patch fiber tips were placed inside the implanted conical shield and laser was applied inside the shield with the same condition as stimulation sessions. Our previous experiments validated that this inter-animal comparison allows us to reduce variability between individuals, and has a similar to greater statistical power compared to the usage of non-opsin GFP control groups in different animals^16^. To control for the unequal number of sessions from individual mice, we averaged behavioral performance across sessions for each mouse and used these mean values (‘Data Analysis’).

#### L-DOPA treatment

L-DOPA (5 mg ml^−1^ in saline, 10 ml per kg body weight) was injected intraperitoneally into APP-KI mice 30 min before the behavioral test (L-DOPA group). For mice in the control group, saline (10 ml per kg body weight) was injected instead. Injections were carried out for 5 consecutive days, and behavioral data during these 5 days were used for the analysis.

#### Photometry recording

The photometry recording was performed using a custom-made photometry setup as described previously^16^. In brief, the excitation laser (473 nm, 0.4–0.5 mW) was delivered through a dichroic and a patch cord (Doric Lenses). Activity-dependent fluorescence emitted by GCaMP-expressing axons was spectrally separated from the excitation light using a dichroic, passed through a single-band filter, and focused onto a photodetector connected to a current preamplifier (SR570, Stanford Research Systems). The output voltage signals from the preamplifiers were collected by an AD board (National Instruments).

### Data analysis

Unless indicated otherwise, analyses were performed using MATLAB codes written by the authors.

#### Behavioral analysis

Behavioral performance was calculated as previously described^16^. Briefly, instantaneous behavioral performance for Odors-A/B and Odors-1/2 was calculated within a sliding window of 20 trials. Percentage correct trials in T5 was calculated from trials 121 to 160. To evaluate the behavioral performance of each mouse, these two values were averaged for all sessions for each mouse. Correct sessions were considered as those with (percent correct trials in T5) >80%.

#### Spike sorting

Spike sorting was performed as previously described^16,39^. Putative principal neurons with spike peak-trough width of more than 230 µs and cells with more than 100 spikes in a session were included for further analyses. For Extended Data Fig. 4, all neurons were analyzed. Double-counted cells were removed by comparing clusters before and after tetrode turns.

#### Spike response

Mean firing rates in 50-ms bins were obtained using a Gaussian filter with a sigma of 100 ms and transformed as a *z*-score using the mean firing rate during the baseline pre-odor period (−1–0 s from odor onset). Significant response was assessed from total spike numbers during odor (0.5–1.5 s after odor onset), delay (2–3 s after odor onset) and choice (3–4 s after odor onset) periods, compared to those in 1-s pre-odor period (Wilcoxon signed-rank test). We used 0.5–1.5 s after odor onset for the odor response period because of the delay from odor delivery to the onset of LEC activity observed in this and previous studies^15,16^.

#### Principal component analysis

PCA was performed as previously described^16^. Principal components 1 and 2 were used for the subsequent analyses. Euclidean distances were calculated between each odor trial type. The Euclidean distance during the odor period of 0.5–1.5 s after odor onset was assessed as mean distance between odor trial types.

#### Shuffle analysis

Shuffle analysis was used to statistically evaluate the mean distances between odor types^15,16^. Shuffle data were obtained by randomly shuffling the assignment of odor type for each trial while keeping the total number of each trial type same as the original real data. With this shuffling procedure, the distinct response to specific odor types observed in the original data disappeared, producing randomly distributed spike responses across each odor trial type. Mean distance during the 1.5 s period of 0.5–1.5 s after odor onset was obtained for each shuffled data. This procedure was repeated 1,000 times, producing 1,000 distances for each odor pair. The upper 95th percentile of 1,000 distances from each odor pair was averaged and used as a threshold for the statistical assessment.

#### Similarity index

A mean shuffle distance was obtained by averaging the shuffle distance across T1–T5. SI was calculated as the difference between real and mean shuffled distance, normalized by the mean shuffled distance^16^:

$${\rm{SI}}={\textstyle \mathrm{--}}({{\rm{mean}}\,{\rm{distance}}}_{{\rm{real}}{\rm{\_}}{\rm{data}}}{\textstyle \mathrm{--}}{{\rm{mean}}\,{\rm{distance}}}_{{\rm{shuffle}}})/{{\rm{mean}}\,{\rm{distance}}}_{{\rm{shuffle}}}$$

The numerator of SI was negatively flipped so that SI becomes positive if the distance obtained from a given odor pair was smaller than shuffle distance (similar representations of two odors), and negative if the distance was larger than shuffle distance (dissimilar representations of two odors).

#### Bootstrapping analysis

The change of SI during associative learning was compared using the bootstrapping method as previously described^16^. Briefly, PCA was performed from a resampled neuronal population, and this procedure was repeated 1,000 times to make 1,000 bootstraps. SI was calculated for each bootstrap, then SIs in T2–T5 were subtracted by that in T1, to test for a significant distribution above or below 0. Distribution of difference with *P* < 0.05 above or below 0 was considered significant. The bootstraps were also compared between WT and APP-KI mice.

#### Decoding analysis

Mean firing rates in 50-ms bins during 1–4 s after odor onset for each odor type (A, B, 1 and 2) were normalized and processed for decoding analysis using binary support vector machine learners. Mean firing rates for Odor-A and Odor-B were used to train the support vector machine. To test performance of prediction, mean firing rates for Odor-1 and Odor-2 were used. This procedure was repeated 10 times to make 10 bootstraps. For each bootstrap, the decoding performance rate predicting Odor-1 or Odor-2 during the 3-s period from 2–5 s after odor onset was calculated.

### Histology and reconstruction of recording positions

#### Electrode positions

Recording positions were confirmed by passing electrical current through tetrodes in anesthetized mice, followed by fixation in 4% paraformaldehyde and cryosectioning^16,39^. Only data from tetrodes in LEC were collected for analysis. Electrode position in each recording session against layer 2/3 of the LEC was estimated using following two criteria: (1) extrapolated position from the histological lesioning and tetrode turning history, and (2) numbers of cells obtained in each session. Typically, numbers of cells become maximum when tetrodes hit the LEC_L2/3_. Using this estimated position, we collected neurons ± 200 µm from putative LEC_L2_.

#### Immunostaining

Sections were rinsed three times for 10 min in 1× PBS (pH 7.6) at room temperature, then preincubated for 1 h in 10% normal goat serum in PBST (1× PBS with 0.5% Triton X-100). Between incubation steps, sections were rinsed in PBST. Sections were incubated with antibodies against TH, raised in rabbit (MB152, Millipore, 1:1,000), or amyloid beta, raised in mouse (McSA1, MEDIMABS, 1:500) for 24 h in antibody-blocking buffer at 4 °C. After three 15-min washes in PBST at room temperature, sections were incubated in a goat anti-rabbit antibody conjugated with Alexa Fluor 488 nm (ab150077, Abcam, 1:250), or goat anti-mouse antibody conjugated with Alexa Fluor 488 nm (A-11001, Thermo Fisher Scientific, 1:250) for 2 h at room temperature. After rinsing in PBS, sections were mounted onto glass slides with 4′,6′-diamidino-2-phenylindole-containing mounting solution (SouthernBiotech), and a coverslip was applied. Digital photomicrographs were acquired with an Olympus BX53 fluorescence microscope equipped with a digital camera.

#### Axon and cell density counting

For density analysis, images were taken spanning 3.4–3.8 mm AP from bregma. The LEC is delineated, and the areas of pixels with fluorescence values higher than three standard deviations of background were quantified for all blocks using ImageJ software, and then divided by the whole area of the image for percentage.

The density of labeled neurons from the TH^+^ population in VTA and SNc was evaluated by anti-TH immunostaining. Images were taken from each hemisphere spanning 3.4–3.6 mm AP from bregma. The number of cells with fluorescence was counted using ImageJ software, and then divided by the whole area of the image for percentage. All analyses were performed blind.

### Statistics and reproducibility

Data are shown with mean ± standard error. The animal numbers and sampled neuron numbers (biological replicates) were designed to achieve a power of greater than 0.8, using MATLAB and G*Power softwares^40^ using effect sizes obtained from similar experiments in our previous papers^16,41^. For statistical testing, data were first tested for normal distribution using the Kolmogorov–Smirnov test (*P* < 0.05 cut-off). All statistical methods used are summarized in Supplementary Table 1 and were two-sided.

### Reporting summary

Further information on research design is available in the Nature Portfolio Reporting Summary linked to this article.

## Online content

Any methods, additional references, Nature Portfolio reporting summaries, source data, extended data, supplementary information, acknowledgements, peer review information; details of author contributions and competing interests; and statements of data and code availability are available at 10.1038/s41593-026-02260-w.

## Supplementary information

Supplementary Information Supplementary Figs. 1–11 and Tables 1–4.

Reporting Summary

## Data Availability

Neurophysiological data are available at https://www.igarashilab.org/resource.

## Code availability

We used standard functions in MATLAB and no special functions were developed. Analytical codes are available at https://www.igarashilab.org/resource.
