# Supplementary material for: Early dopamine disruption in the entorhinal cortex of a knock-in model of Alzheimer’s disease
Source: Nat Neurosci. 2026 Apr 23;29(6):1386–96. doi: 10.1038/s41593-026-02260-w (PMC13246448; doi:10.1038/s41593-026-02260-w)
Supplement: Supplementary file 1 — Supplementary Figs. 1–11 and Tables 1–4. [file 41593_2026_2260_MOESM1_ESM.pdf]

nature neuroscience

Supplementary information https://doi.org/10.1038/s41593-026-02260-w

Early dopamine disruption in the entorhinal cortex of a knock-in model of Alzheimer's disease

In the format provided by the authors and unedited

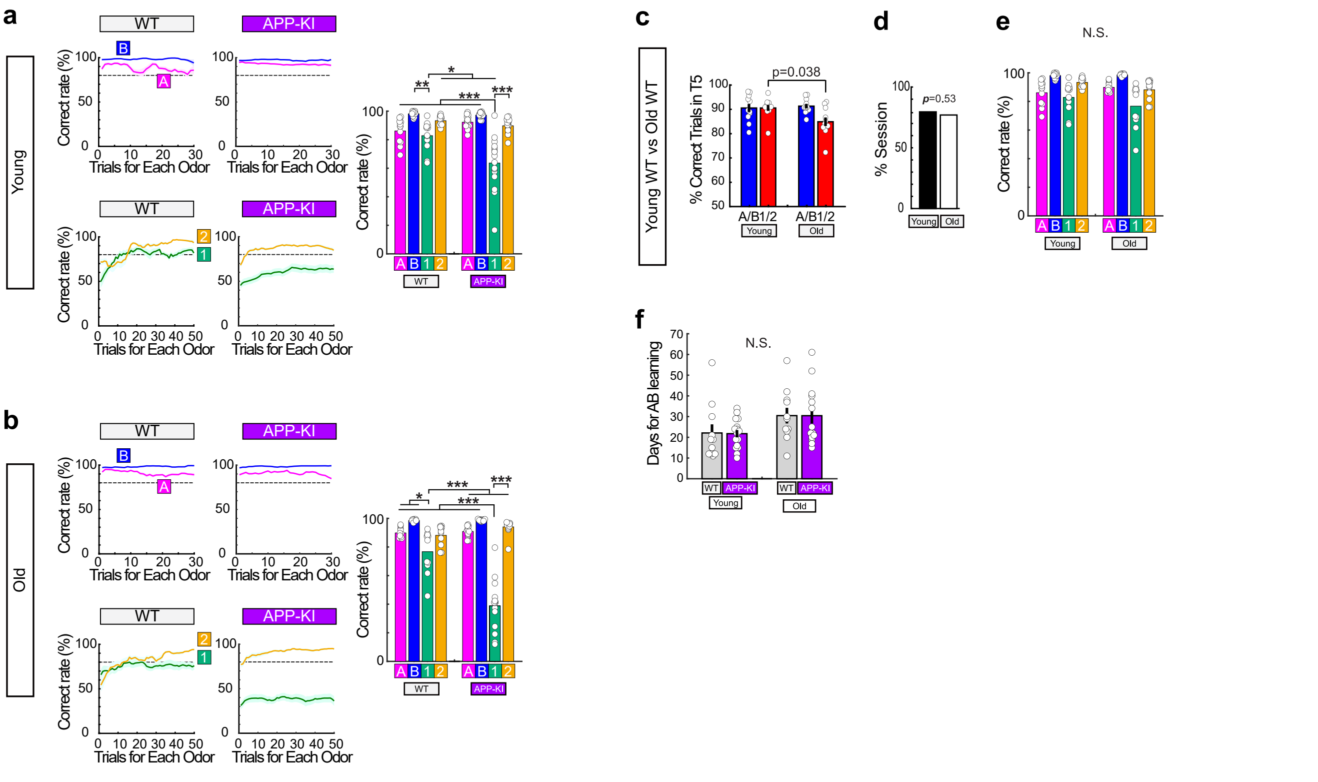

Supplementary Figure 1 | Detailed behavior analysis of WT and APP-KI mice

a. Detailed behavioral performance of young WT and young APP-KI mice in Fig. 1c-d, plotted for each odor trial type. Learning curves during young WT (Left) and young APP-KI (Middle). (Right) Performance of mice in T5 ($F_{3,100}$=9.88, p=9.1e-6, odor x strain interaction, ANOVA; $p_{\text{odorA-WTvsodorB-WT}}$ = 6.6e-4, $p_{\text{odorA-WTvsodorB-APP}}$ =0.019, $p_{\text{odorA-WTvsodor1-WT}}$ = 3.5e-7, $p_{\text{odorB-WTvsodor1-WT}}$ = 2.6e-3, $p_{\text{odorB-WTvsodor1-APP}}$ = 3.8e-15, $p_{\text{odor1-WT-vsodorB-APP}}$ = 1.9e-3, $p_{\text{odor1-WTvsodor1-APP}}$ = 8.1e-6, $p_{\text{odor2-WTvsodor1-APP}}$ =2.4e-13, $p_{\text{odorA-APPvsodor1-APP}}$ = 2.4e-13, $p_{\text{odorB-APPvsodor1-APP}}$ = 6.9e-17, $p_{\text{odor1-APPvsodor2-APP}}$ = 1.3e-11, two-sided Tukey post hoc test; n = 11 young WT mice, n= 16 young APP-KI mice). Other p-values not shown in the figure were non-significant.

b. Same as Supplementary Fig. 1a but for old WT and old APP-KI mice. (mean ± s.e.m.; $F_{3,92}$=30.11, p=1.2e-13, odor x strain interaction, ANOVA; $p_{\text{odorA-WTvsodor1-WT}}$ = 0.024, $p_{\text{odorA-WTvsodor1-APP}}$ =5.3e-28, $p_{\text{odorB-WT-vsodor1-WT}}$ = 6.1e-6, $p_{\text{odorB-WTvsodor1-APP}}$ = 7.3e-28, $p_{\text{odor1-WTvsodorA-APP}}$ = 5.5e-3, $p_{\text{odor1-WTvsodorB-APP}}$ = 1.2e-6, $p_{\text{odor1-WTvsodor1-APP}}$ = 1.5e-15, $p_{\text{odor1-WTvsodor2-APP}}$ =2.3e-4, $p_{\text{odor2-WTvsodor1-APP}}$ = 4.4e-22, $p_{\text{odorA-APPvsodor1-APP}}$ = 1.8e-25, $p_{\text{odorB-APPvsodor1-APP}}$ = 2.7e-30, $p_{\text{odor1-APPvsodor2-APP}}$ = 2.2e-27, two-sided Tukey post hoc test; n = 11 old WT mice, n= 14 old APP-KI mice).

c. Percentage of correct trials in T5 of young WT vs old WT mice (mean ± s.e.m.; $F_{1,40}$=5.04, p=0.031, odor x strain interaction, ANOVA; $p_{\text{odorA/B-youngvsodor1/2-young}}$ = 0.038, $p_{\text{odor1/2-youngvsodor1/2-old}}$ = 0.038, $p_{\text{odorA/B-old-vsodor1/2-old}}$ = 0.014, two-sided Tukey post hoc test; n = 11 young WT mice, n = 11 old WT mice).

d. Percentage of sessions where mice correctly learned new association (p=0.53, binomial test).

e. Performance of young and old WT mice in T5, plotted for each odor trial type. (mean ± s.e.m.; $F_{3,80}$=2.25, p=0.71, odor x strain interaction, ANOVA; n = 11 young WT mice, n= 11 old WT mice).

f. Days required for obtaining performance over the criteria (80%) during pre-learning sessions in WT (n = 11 young WT mice, n= 11 old WT mice) and APP-KI (n= 16 young APP-KI mice, n= 14 old APP-KI mice) mice (mean ± s.e.m.; $F_{1,48}$=0.0022, p=0.96, strain x age interaction, ANOVA). Statistical significance is indicated as *p < 0.05, **p < 0.01, ***p < 0.001.

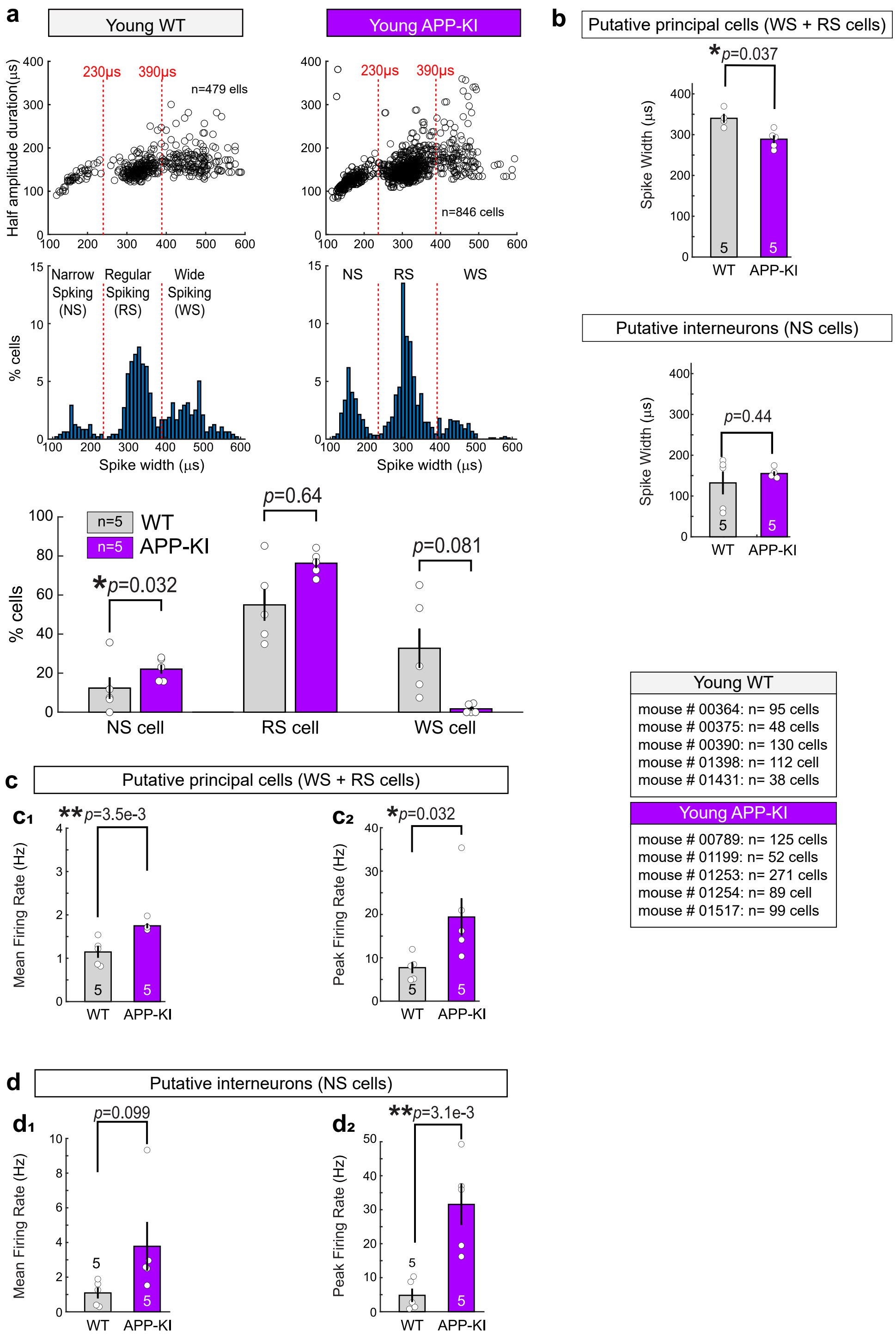

Supplementary Figure 2 | Spike properties of LECL2/3 neurons in young WT and young APP-KI mice, presented on an animal-by-animal basis

Same figure as Extended Data Fig. 4, but using animals as a unit of analysis (*n*=5 young WT and *n*=5 APP-KI mice).

a. Spike properties of LECL2/3 neurons. Top and middle panels are the same as Extended Data Fig. 4a. (Top and middle). Bottom, Percentage of NS, RS, and WS cells in young WT and young APP-KI mice ($F_{2,24}$ = 8.5, p= 1.5e-3, spike type x strain, ANOVA; p_{NS} = 0.032, p_{WS} = 0.031, two-sided Tukey post hoc test; n = 5 mice per strain: young WT and young APP-KI mice).

b. Mean spike widths of putative principal cells (RS cells and WS cells; $t(8)$ =2.49, p = 0.037, two-sided unpaired t-test) and putative interneurons (NS cells; $t(8)$ =-0.81, p=0.44, two-sided unpaired t-test) between young WT and young APP-KI mice (n = 5 mice per strain).

c. Mean and peak firing rates of LECL2/3 putative principal cells throughout the associative memory learning session same as Extended Fig. 4c but for bar plots presented on animal-by-animal basis. (c1) Mean firing rate ($t(8)$ =-4.1, p=3.5e-3, two-sided unpaired t-test). (c2) Peak firing rate ($t(8)$ =-2.6, p=0.032, two-sided unpaired t-test).

d. Same as Extended Data Fig. 4d, but for LECL2/3 putative interneurons (Mean firing rate, $t(8)$ =-1.8, p=0.099, two-sided unpaired t-test; Peak firing rate, $t(8)$ =-4.2, p=3.1e-3, two-sided unpaired t-test).

All data are presented as mean ± s.e.m.

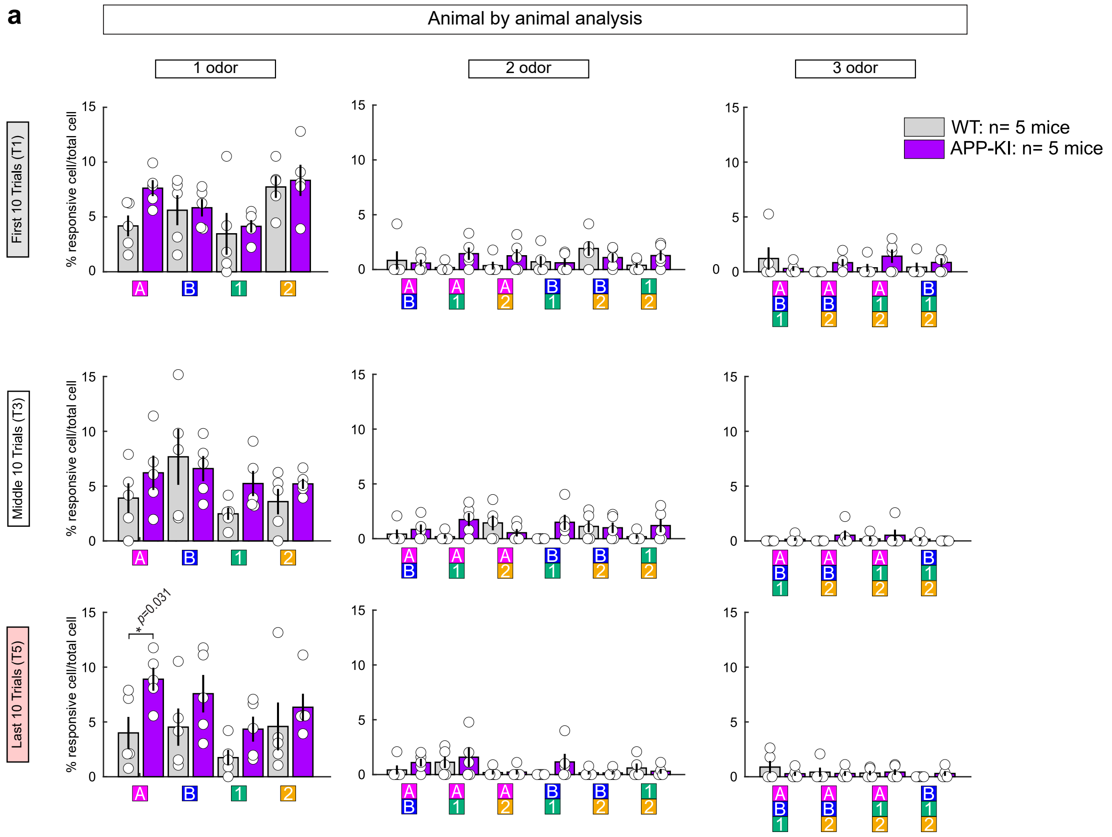

Supplementary Figure 3 | Cue response type of LECL2/3 neurons in young WT and young APP-KI mice, presented on an animal-by-animal basis

Same figure as Extended Data Fig. 5, but using animals as a unit of analysis (*n*=5 young WT and *n*=5 APP-KI mice). LECL2/3 principal neurons were classified for their cue response type during 0.5-1.5 s after cue onset and shown in percentage among all recorded neurons. Neurons were collected from both correct and error sessions. APP-KI mice had more Odor-A cells and Odor-A/1 cells compared to WT mice at T1 and T5. Top, first 10 trials (T1); middle, 10 trials (T3); bottom, last 10 trials (T5) (Odor A responsive cell, p=0.031 in T5, two-sided unpaired t-test followed by FDR correction for multiple comparison; n = 5 mice from young WT vs. n= 5 mice from young APP-KI mice). Data are presented as mean ± s.e.m.

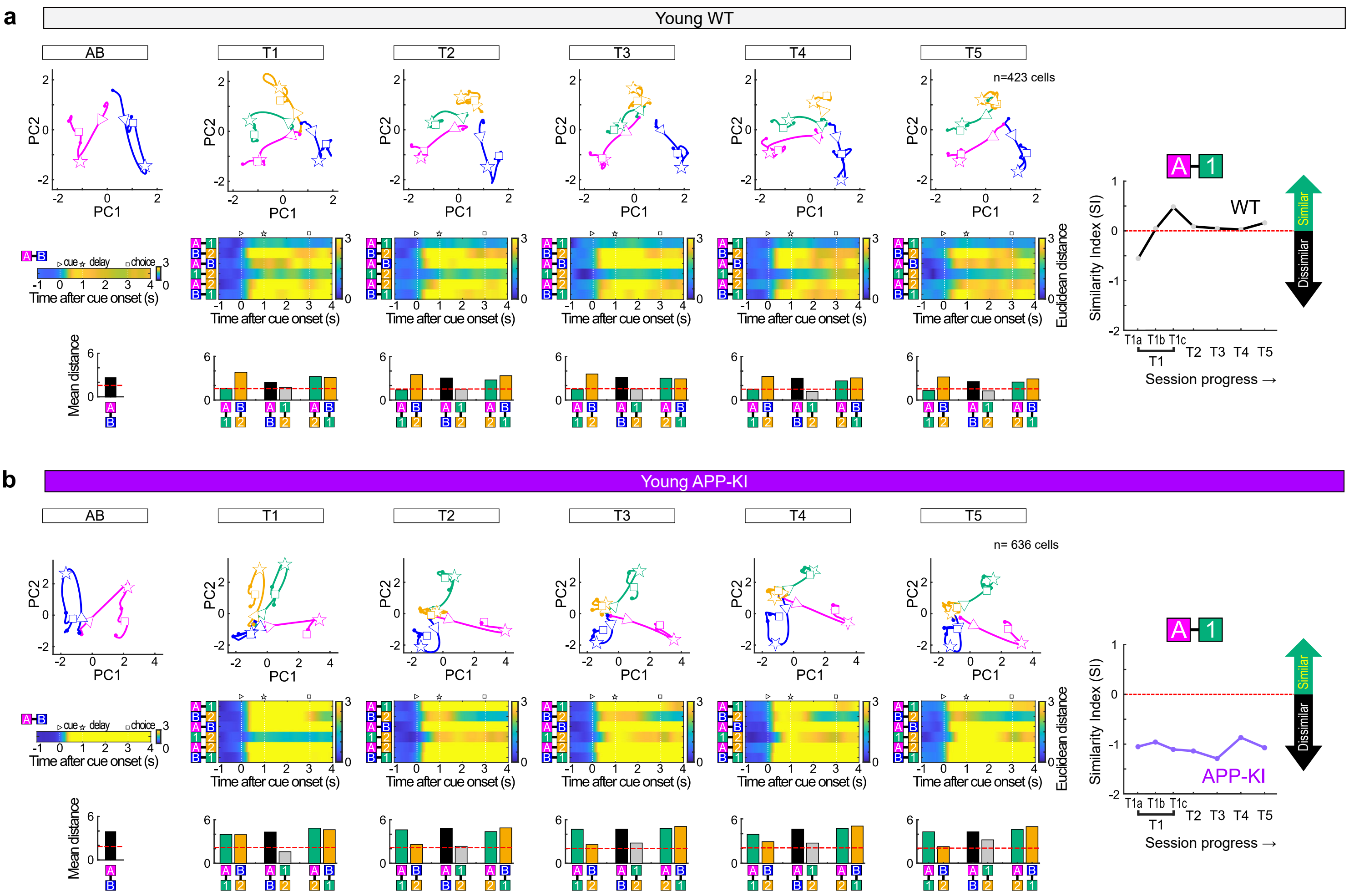

Supplementary Figure 4 | Principal component analyses of LECL2/3 neurons of young WT and young APP-KI mice through T1 – T5

a. PCA trajectories of neural firing of LECL2/3 cell population in young WT mice as in Fig. 2e, 2g, 2h but presented throughout timepoints T1 – T5. T1 is subdivided into T1a (trials 1-3), T1b (trials 4-6) and T1c (trials 7-9).

b. PCA trajectories of neural firing of LECL2/3 cell population in young APP-KI mice as in Fig. 2f, 2g, 2h but presented throughout timepoints T1 – T5.

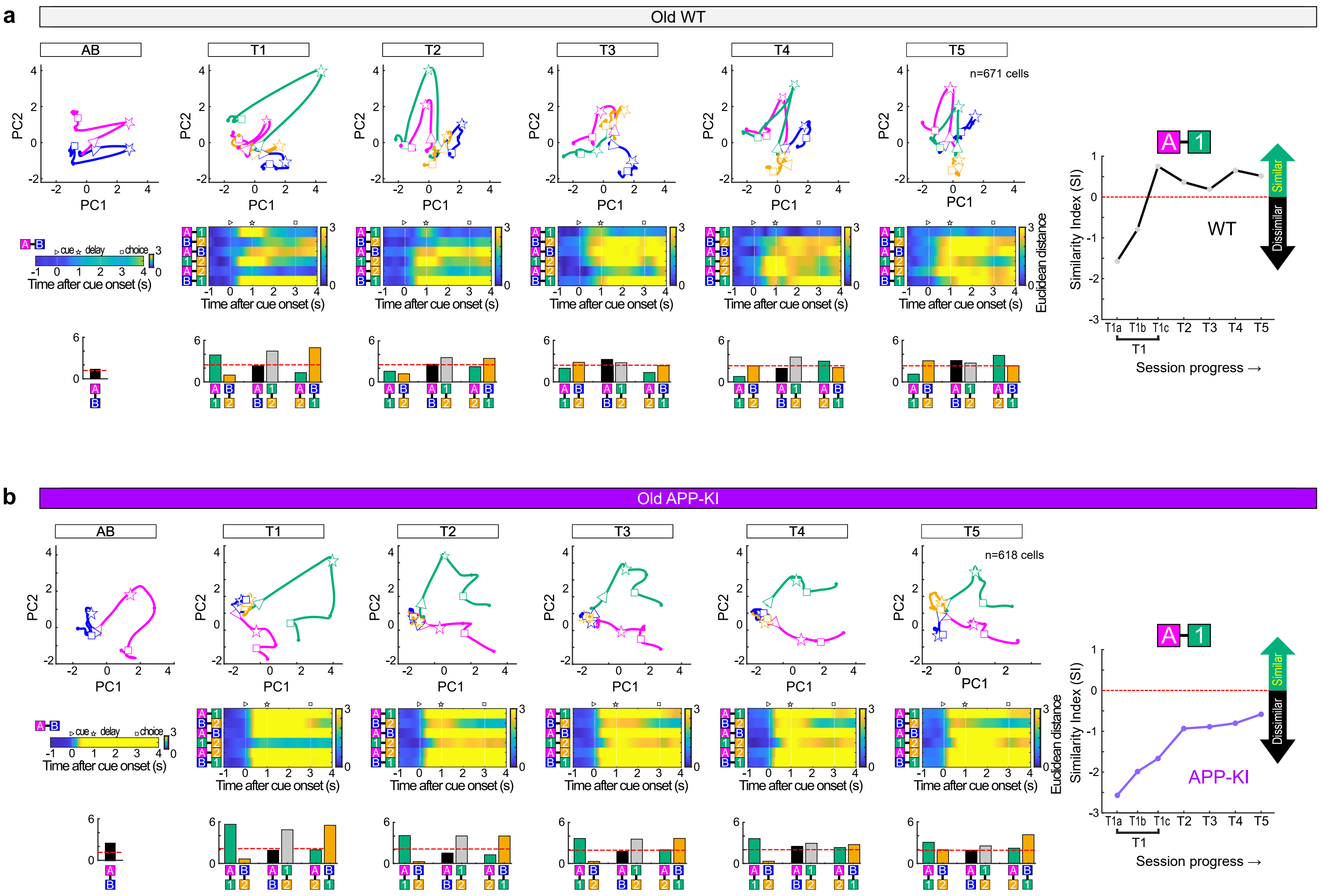

Supplementary Figure 5 | Principal component analyses of LECL2/3 neurons of old WT and old APP-KI mice through T1 – T5

a. PCA trajectories of neural firing of LECL2/3 cell population in old WT mice as in Extended Data Fig. 9c, 9e, 9f but presented throughout timepoints T1 – T5. T1 is subdivided into T1a (trials 1-3), T1b (trials 4-6) and T1c (trials 7-9).

b. PCA trajectories of neural firing of LECL2/3 cell population in old APP-KI mice as in Extended Data Fig. 9d, 9e, 9f but presented throughout timepoints T1 – T5.

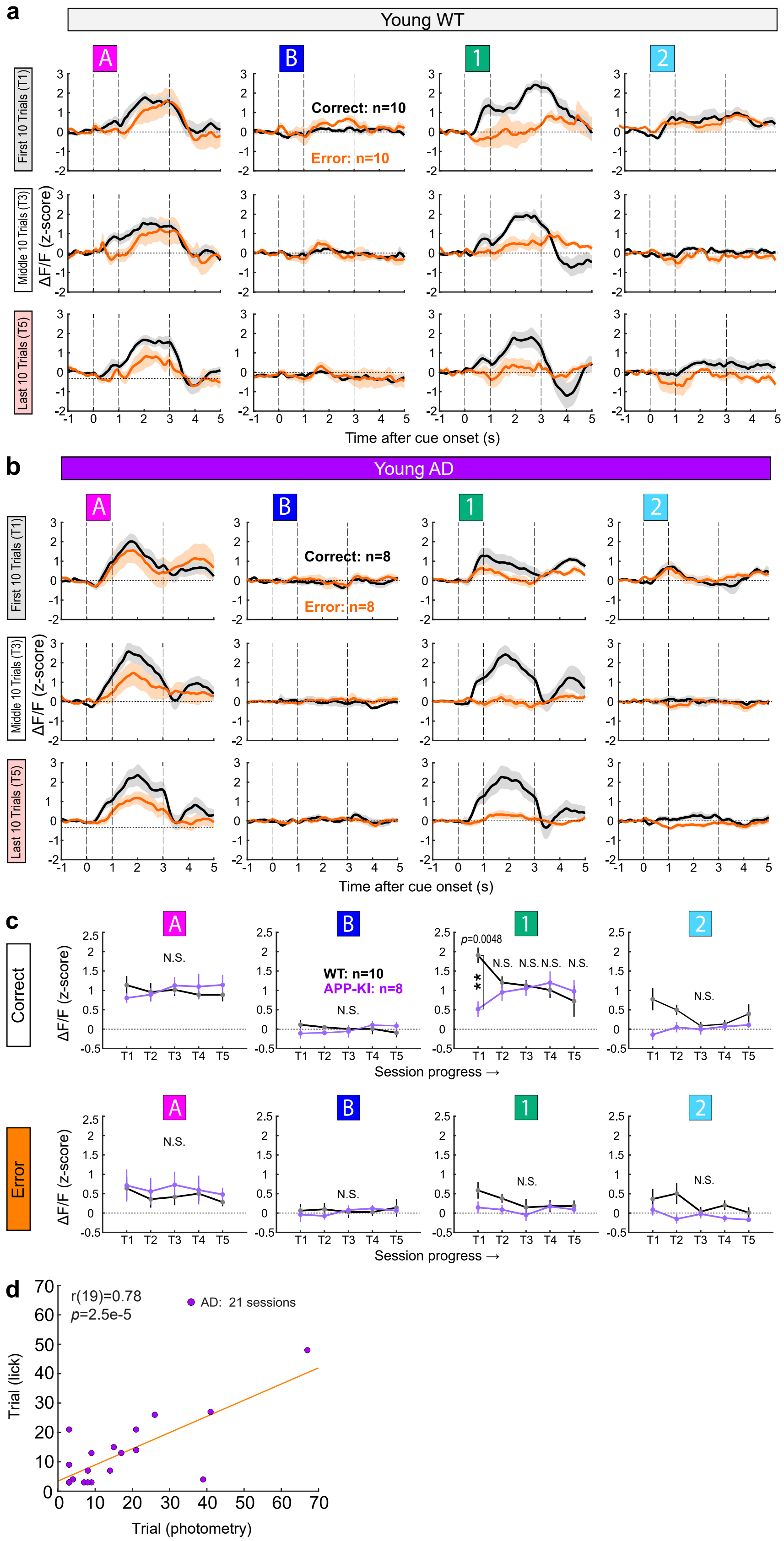
a
Young WT
A
B
1
2
First 10 Trials (T1)
Middle 10 Trials (T3)
Last 10 Trials (T5)
ΔF/F (z-score)
Correct: n=10
Error: n=10
Time after cue onset (s)
b
Young AD
Correct: n=8
Error: n=8
Time after cue onset (s)
c
Correct
Error
WT: n=10
APP-KI: n=8
N.S.
p=0.0048
**
T1 T2 T3 T4 T5
Session progress →
d
r(19)=0.78
p=2.5e-5
AD: 21 sessions
Trial (lick)
Trial (photometry)

Supplementary Figure 6 | LEC dopamine signals in correct and error sessions

a. Same as Fig. 4d, but for data from young WT mice separated for correct (black) and error (orange) sessions. Data are presented as mean ± s.e.m.

b. Same as Fig. 4d, but for data from young APP-KI mice separated for correct (black) and error (orange) sessions. Data are presented as mean ± s.e.m.

c. Mean GCaMP signals during 1-4 s after cue onset, plotted separately for correct sessions of young WT and APP-KI mice (top; Odor-1; $F_{4,80}$=3.72, p=7.8e-3, strain x time interaction, ANOVA; p_{T1} = 4.8e-3, two-sided Tukey post hoc test; n = 10 and n = 8 hemispheres from WT and APP-KI mice, respectively), and error sessions of young WT and APP-KI mice (bottom; N.S. p > 0.05, ANOVA; n = 10 and n = 8 hemispheres from WT and APP-KI mice, respectively). Data are presented as mean ± s.e.m.

d. Correlation between behavioral performance and photometry signals for Odor-1. Y-axis shows the trial number after which mice started five consecutive hit trials for Odor-1. X-axis denotes the trial number after which five consecutive trials exhibited GCaMP signals > 3 times standard deviation (SD). Note this 3SD criterion removed some sessions with low GCaMP signals. Each dot represents a single session from young APP-KI mice (p = 2.5e-5, two-sided Pearson's correlation; n = 21 sessions form n = 8 young APP-KI mice).

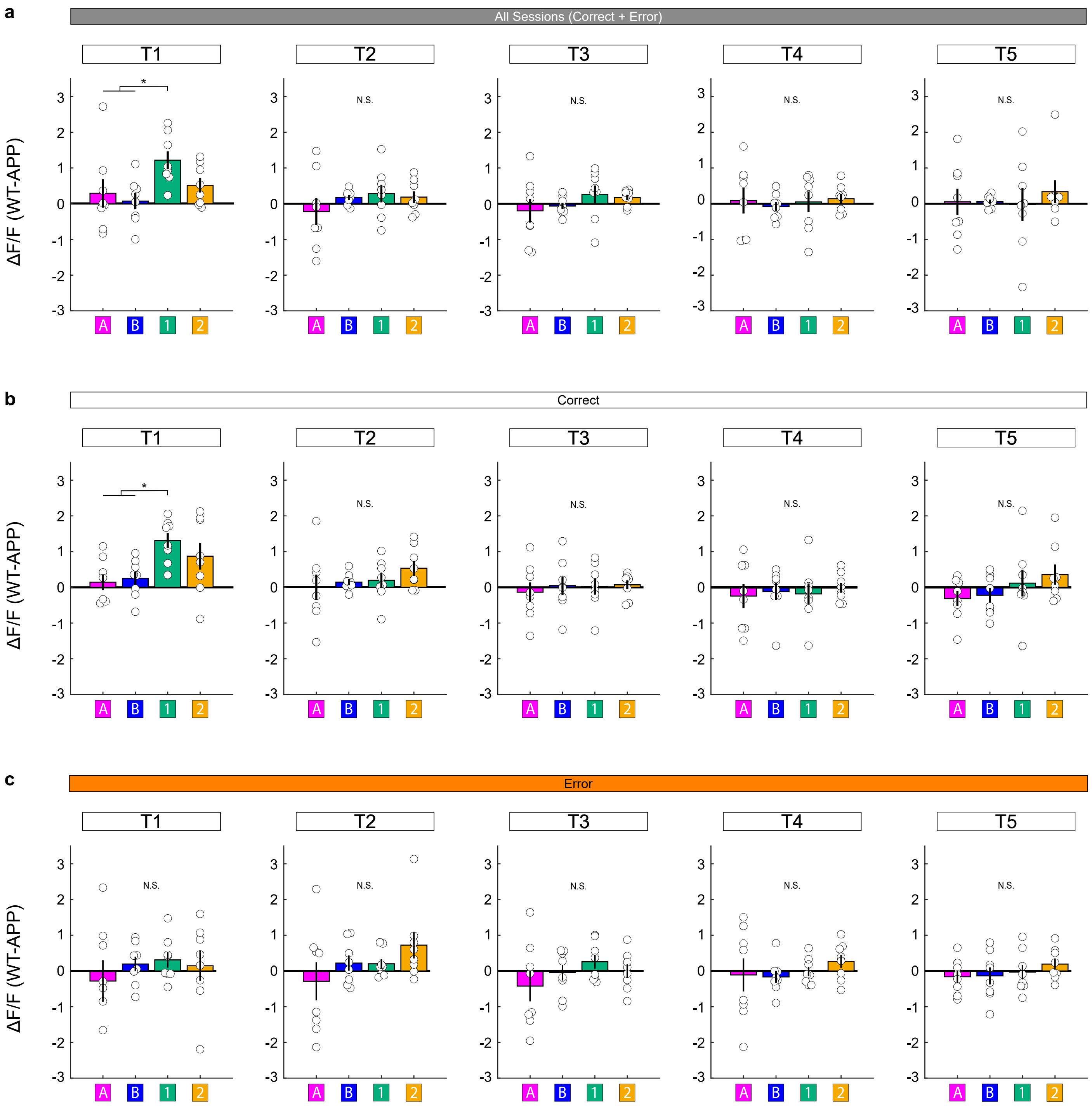

Supplementary Figure 7 | Difference of LEC dopamine signals between WT and APP-KI mice across odor cues

a. Difference of dopamine activity between WT and APP-KI mice (WT minus KI) were compared across odor cues. All data (during correct and error sessions) were used. Decrease of dopamine for Odor-1 was significantly larger than those for Odor-A or Odor-B ($F_{3,28}$=3.98, p=0.017, group, ANOVA; $p_{\text{odorAvsodor1}}$=0.042, $p_{\text{odorBvsodor1}}$=0.041 in T1, two-sided Tukey post hoc test).

b. Same as Supplementary Fig.7a, but for data during correct session. Decrease of dopamine for Odor-1 was again significantly larger than those for Odor-A or Odor-B ($F_{3,28}$=4.41, p=0.011, group, ANOVA; $p_{\text{odorAvsodor1}}$=0.018, $p_{\text{odorBvsodor1}}$=0.036 in T1, two-sided Tukey post hoc test).

c. Same as Supplementary Fig.7a, but for data during error session. No significant difference was observed.

Data are presented as mean ± s.e.m. unless otherwise indicated.

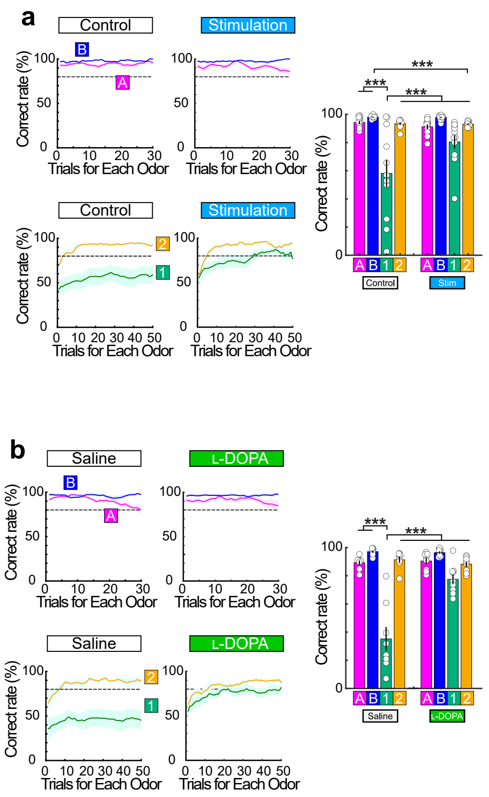

Supplementary Figure 8 | Detailed behavior analysis for optogenetic LEC dopamine stimulation and L-DOPA treatment

a. Detailed behavioral performance for dopamine fiber optogenetic stimulation in young APP-KI x DAT-Cre mice in Fig. 5b-d, but plotted for each odor trial type. Learning curves during control (left) and stimulation (middle) sessions. (Right) Performance of mice in the last 10 trials ($F_{3,80}$=4.48, p=5.8e-3, odor x stim interaction, ANOVA; $p_{odorA-Ctrvsodor B-Ctr}$ = 1.9e-7, $p_{odorB-Ctrvsodor1-Ctr}$ =9.8e-0, $p_{odorB-Ctrvsodor1-Stim}$ = 0.044, $p_{odor1-Ctrvsodor2-Ctr}$ = 3.8e-7, $p_{odor1-CtrvsodorA-Stim}$ = 1.9e-6, $p_{odor1-CtrvsodorB-Stim}$ = 1.2e-8, $p_{odor1-Ctrvsodor1-Stim}$ = 3.4e-3, $p_{odor1-Ctrvsodor2-Stim}$ = 4.9e-7, two-sided Tukey post hoc test; n = 11 mice). Other p-values not shown in the figure were non-significant.

b. Same as Supplementary Fig. 8a but for L-DOPA treatment in young APP-KI mice. ($F_{3,60}$=11.2, p=5.9e-6, odor x drug interaction, ANOVA; $p_{odorA-Salvsodor1-Sal}$ = 2.6e-10, $p_{odorB-Salvsodor1-Sal}$ = 1.7e-2, $p_{odorB-Salvsodor1-ldopa}$ = 1.7e-2, $p_{odor1-Salvsodor2-Sal}$ = 4.5e-11, $p_{odor1-SalvsodorA-ldopa}$ = 2.2e-11, $p_{odor1-SalvsodorB-ldopa}$ = 3.5e-13, $p_{odor1-Salvsodor1-ldopa}$ = 2.9e-7, $p_{odor1-Salvsodor2-ldopa}$ = 9.3e-11, $p_{odorB-ldopavsodor1-ldopa}$ = 1.4e-2, post-hoc Tukey test; n = 8 mice for saline, n =9 for L-DOPA). Other two-sided Tukey post hoc test p-values not shown in the figure were non-significant.

All data are presented as mean ± s.e.m.

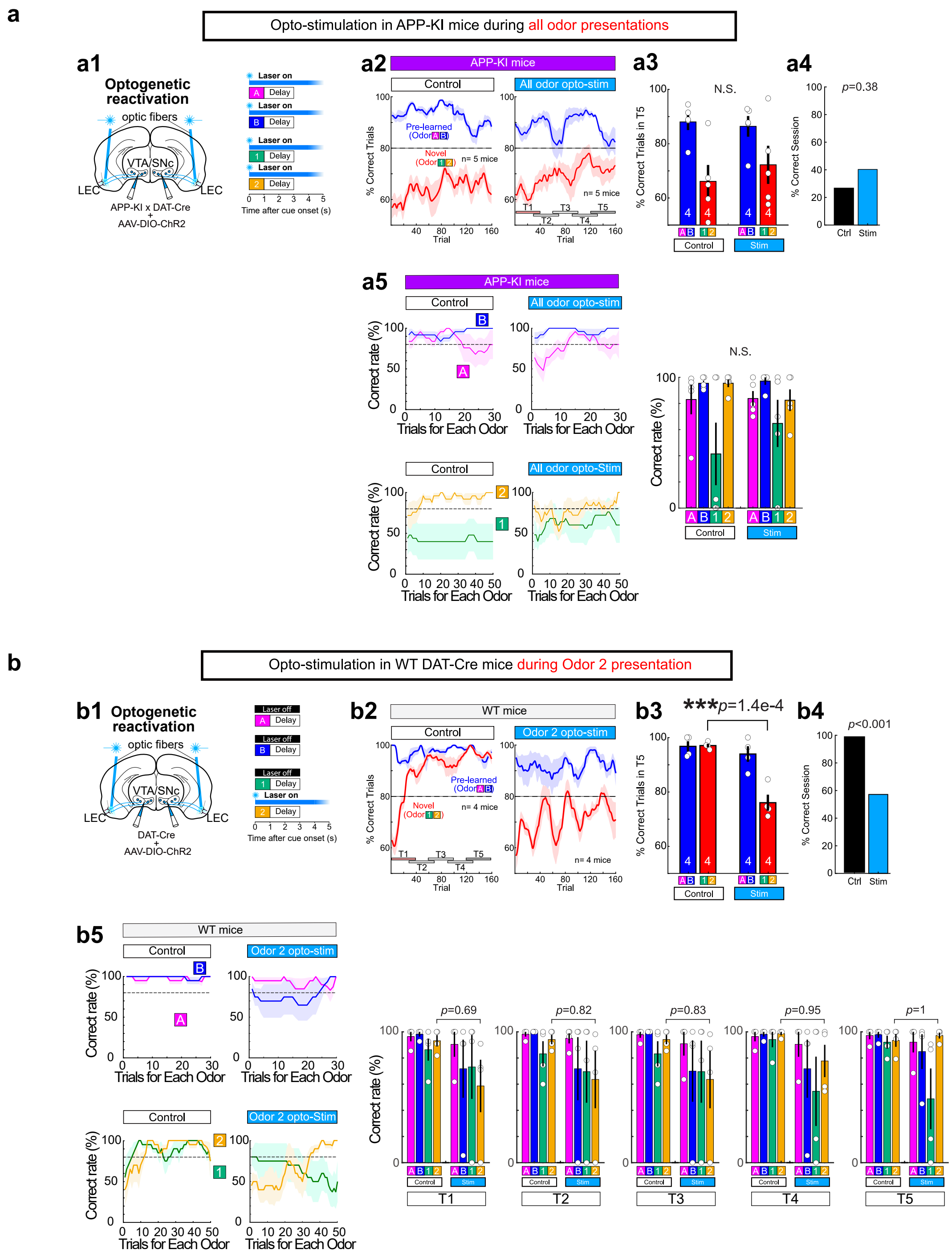

Supplementary Figure 9 | Control additional optogenetic stimulation experiments

a. Optogenetic stimulation of dopamine fibers in the LEC during all odor representations in young APP-KI mice (**a1**). This experiment did not have an effect to rescue associative memory in APP-KI mice.
a2: Correct trial rate for pre-learned odors (A/B, blue) and novel odors (1/2, red) in control no-stimulation sessions (Left) and optogenetic stimulation sessions (Right), obtained from n=5 young APP-KI mice. **a3**: Percentage of correct trials in T5 of control sessions and optogenetic stimulation sessions ($F_{1,16}$=0.55, p=0.47, odor x stim interaction, ANOVA; n = 5 young APP-KI mice). **a4**: Percentage of sessions where mice correctly learned new association (p=0.38, binomial test). **a5**: Detailed behavioral performance of control and optogenetic stimulation sessions, plotted for each odor trial type. Learning curves during control (Left) and young optogenetic stimulation (Middle). (Right) Performance of mice in T5 ($F_{3,32}$=0.78, p=0.51, odor x stim interaction, ANOVA).

b. Optogenetic stimulation of dopamine fibers in the LEC during Odor-2 representation in young WT mice (**b1**). The stimulation during Odor-2 impaired associative memory in WT mice.
b2: Correct trial rate for pre-learned odors (A/B, blue) and novel odors (1/2, red) in control no-stimulation sessions (Left) and optogenetic stimulation sessions (Right), obtained from n=4 young WT mice. **b3**: Percentage of correct trials in T5 of control sessions and optogenetic stimulation sessions ($F_{1,16}$=15.83, p=0.018, odor x stim interaction, ANOVA; $p_{odorA/B\text{-}Ctr vs odor1/2\text{-}Stim}$ = 1.7e-3, $p_{odor1/2\text{-}Ctr vs odor1/2\text{-}Stim}$ = 1.4e-4, $p_{odorA/B\text{-}Stim vs odor1/2\text{-}Stim}$ = 6.3e-3, , two-sided Tukey post hoc test). **b4**: Percentage of sessions where mice correctly learned new association (p<0.001, binomial test). **b5**: Detailed behavioral performance of control and optogenetic stimulation sessions, plotted for each odor trial type. Learning curves during young control (Left) and young optogenetic stimulation (Middle). (Right) Performance of mice throughout T1 to T5 (p>0.05, odor x stim interaction, ANOVA).
Data are presented as mean ± s.e.m. unless otherwise indicated.

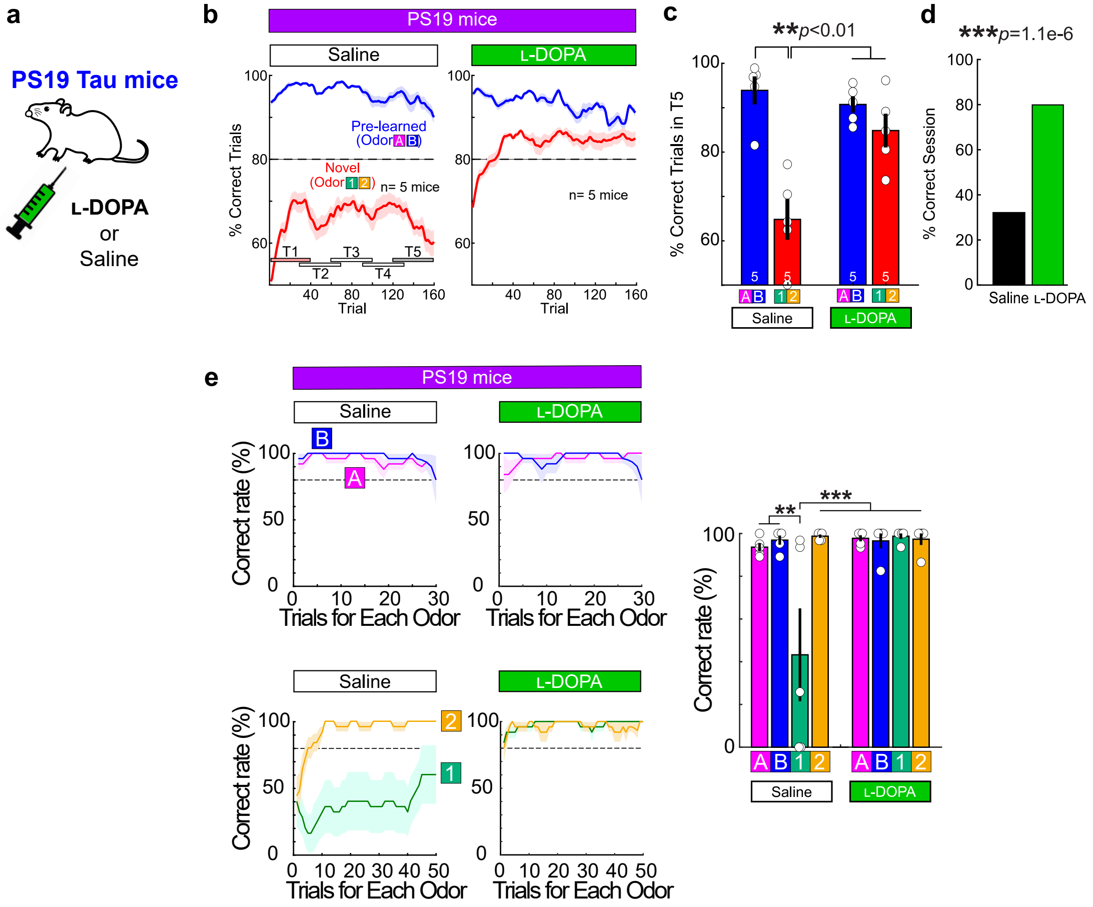

Supplementary Figure 10 | L-DOPA treatment in young PS19 mice

The L-DOPA experiment in Figure 6a-c was repeated using PS19 mice, a widely-used tau mouse model. L-DOPA treatment again restored associative memory in PS19 mice.

a. Schema of L-DOPA treatment in young PS19 mice.

b. Correct trial rate for pre-learned odors (A/B, blue) and novel odors (1/2, red) in saline-injected (Left) and L-DOPA-injected mice (Right), obtained from *n*= 5 saline-injected mice and *n*= 5 L-DOPA injected mice.

c. Percentage of correct trials in T5 of saline- and L-DOPA-injected mice ($F_{1,16}$=9.88, *p*=9.1e-6, odor x drug interaction, ANOVA; $p_{\text{odorA/B-Salvsodor1/2-Sal}}$ = 1.2e-4, $p_{\text{odor1/2-SalvsodorA/B-ldopa}}$ = 1.2e-4, $p_{\text{odor1/2-Salvsodor1/2-ldopa}}$ = 4.7e-3, two-sided Tukey post hoc test).

d. Percentage of sessions where mice correctly learned new association (****p*=1.1e-6, binomial test).

e. Detailed behavioral performance of saline- and L-DOPA-injected mice, plotted for each odor trial type. Learning curves during young control (Left) and young optogenetic stimulation (Middle). (Right) Performance of mice in T5 ($F_{3,32}$=5.98, *p*=0.023, odor x drug interaction, ANOVA; ** *p*<0.01, *** *p*<0.001, $p_{\text{odorA-Salvsodor1-Sal}}$ = 2.0e-3, $p_{\text{odorB-Salvsodor1-Sal}}$ = 8.9e-4, $p_{\text{odor1-SalvsodorA-ldopa}}$ = 7.2e-4, $p_{\text{odor1-SalvsodorB-ldopa}}$ = 9.8e-4, $p_{\text{odor1-Salvsodor1-ldopa}}$ = 5.6e-4, $p_{\text{odor1-Salvsodor2-ldopa}}$ = 8.0e-4, two-sided Tukey post hoc test; *n* = 5 saline-injected mice and *n* = 5 L-DOPA injected mice).

All data are presented as mean ± s.e.m.

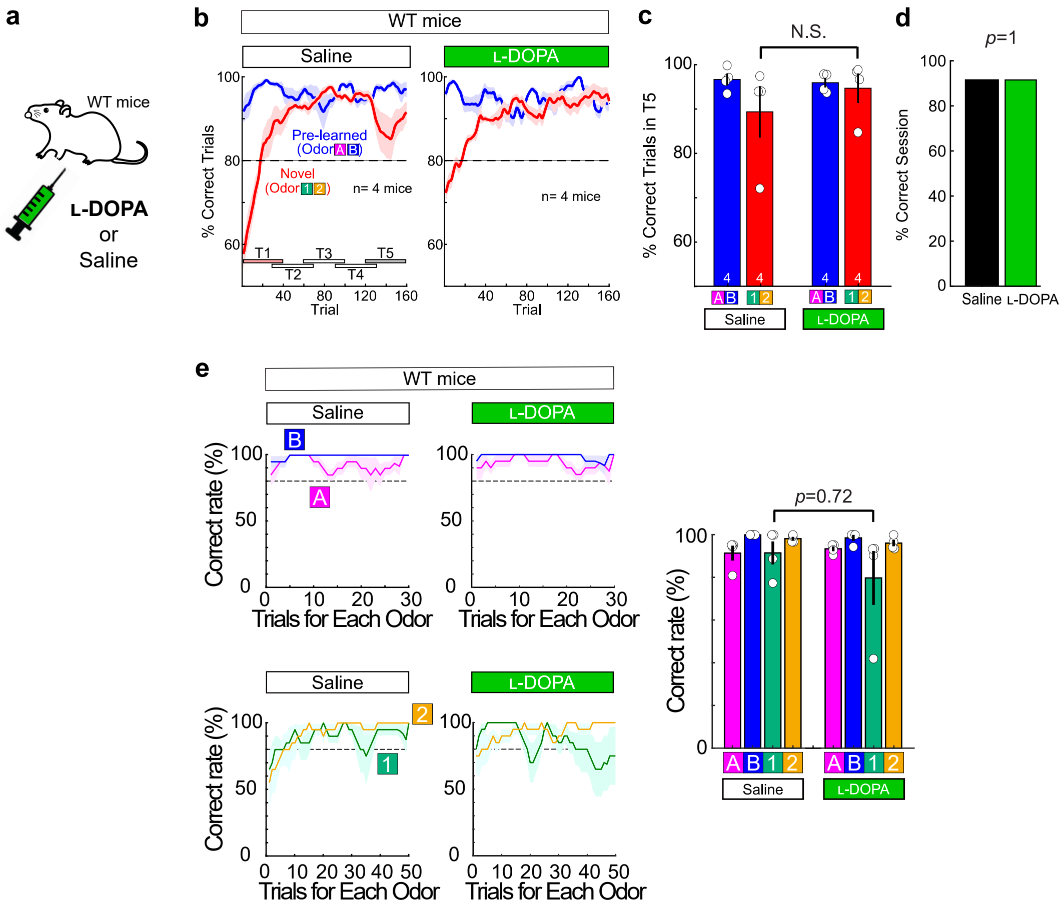

Supplementary Figure 11 | Control L-DOPA injection experiment in young WT mice

The L-DOPA experiment in Figure 6a-c was repeated using control WT mice. No significant difference was observed.

a. Schema of L-DOPA treatment in young WT mice.

b. odors (1/2, red) in saline- (Left) and L-DOPA-injected mice (Right), obtained from *n* = 4 saline-injected mice and *n* = 5 L-DOPA-injected mice.

c. jected mice ($F_{1,4}$ = 0.01, *p* = 0.91 odor x drug interaction, ANOVA).

d. Percentage of sessions where mice correctly learned new association (*p* = 1, binomial test).

e. Detailed behavioral performance of control and optogenetic stimulation sessions, plotted for each odor trial type. Learning curves during young control (Left) and young optogenetic stimulation (Middle). (Right) Performance of mice in T5 ($F_{3,8}$ = 0.012, *p* = 0.99, odor x drug interaction, ANOVA; *n* = 4 saline-injected mice and *n* = 5 L-DOPA-injected mice).

All data are presented as mean ± s.e.m.

Supplementary Table 1. Statistics used in this study

Experiment	Figure	Detail	Sample Size	Analysis	P Value	Post-hoc Analysis	Post-hoc P Value
**Fig. 1**							
Behavior-Young WT mice vs Young APP-KI mice	1d	% Correct Trials in T5:<br>WT mice vs APP-KI mice	Young WT:11 mice<br>Young APP-KI: 16 mice	2-way ANOVA<br>(Odor type [A/B vs 1/2] x Strain [WT vs APP-KI])	Odor: F(1,50)=20.18; p=4.1e-5<br>Strain: F(1,50)=8.58; p=5.1e-3<br>Odor x Strain: F(1,50)=20.2; p=4.1e-5	Tukey test	A/B WT vs 1/2 APP: p=1.8e-5<br>A/B APP vs 1/2 APP: p=3.1e-5<br>1/2 WT vs 1/2 APP: p=3.1e-8<br>NS for other pairs
	1e	% Correct Sessions in T5:<br>WT mice vs APP-KI mice	Young WT:11 mice<br>Young APP-KI: 16 mice	Unpaired t test	t(25) = 2.19, p = 0.037		
Behavior-Old WT mice vs Old APP-KI mice	1g	% Correct Trials in T5:<br>WT mice vs APP-KI mice	Old WT:11 mice<br>Old APP-KI: 14 mice	2-way ANOVA<br>(Odor type [A/B vs 1/2] x Strain [WT vs APP-KI])	Odor: F(1,46)=113.2; *p*=5.4e-14<br>Strain: F(1,46)=22; *p*=2.4e-5<br>Odor x Strain: F(1,46)=41.42; *p*=6.4e-8	Tukey test	A/B WT vs 1/2 WT: p=0.035<br>A/B WT vs 1/2 APP: p=1.7e-13<br>1/2 WT vs AB APP: p=6.6e-4<br>1/2 WT vs 1/2 APP: p=2.7e-9<br>A/B APP vs 1/2 APP: p=4.3e-16<br>NS for other pairs
	1h	% Correct Sessions in T5:<br>WT mice vs APP-KI mice	Old WT:11 mice<br>Old APP-KI: 14 mice	Unpaired t test	t(23) = 3.65, p = 1.3e-3		
**Fig. 2**							
LEC cells recording	2b, 2c	Significance of LEC cell firing in odor period	10 trials	Wilcoxon signed-rank test	p<0.05	For each LEC cell	
LEC cells:<br>Distribution of Response Types between young WT and young APP-KI mice	2d	T1	Young WT: 423 cells<br>Young APP-KI: 636 cells	Chi-Square test	X^2=5.88, p=7.1e-5	Benjamini-Hochberg post-hoc test	1 odor: *p*=1.1e-3<br>2 odor: *p*=0.029<br>3 odor: *p*=0.029<br>no responsive type: *p*=4.4e-6<br>NS for other pairs
		T3			X^2=6.85, p=1.5e-7		1 odor: p=5.4e-5<br>2 odor: p=3.2e-3<br>3 odor: p=0.030<br>no responsive type: p=9.7e-9<br>NS for other pairs
		T5			X^2=2.29, p=3.6e-9		1 odor: p=9.2e-8<br>2 odor: p=1.1e-3<br>no responsive type: p=2.5e-10<br>NS for other pairs
LEC cells recording (young WT mice, correct + error sessions)	S4a	AB-only	Young WT: 423 cells	1000 Shuffling<br>(trial shift)	95th percentile value = 1.56		
	2g, S4a	T1			95th percentile value = 1.57		
	S4a	T2			95th percentile value = 1.55		
	2g, S4a	T3			95th percentile value = 1.51		
	S4a	T4			95th percentile value = 1.56		
	2g, S4a	T5			95th percentile value = 1.59		
LEC cells recording (young APP-KI mice, correct + error sessions)	S4b	AB-only	Young APP-KI:636 cells	1000 Shuffling<br>(trial shift)	95th percentile value = 1.86		
	2g, S4b	T1			95th percentile value = 2.14		
	S4b	T2			95th percentile value = 2.15		
	2g, S4b	T3			95th percentile value = 2.04		
	S4b	T4			95th percentile value = 2.07		
	2g, S4b	T5			95th percentile value = 2.08		
Bootstrapping analyses for Similarity Index	2i, E8e	Similarity Index for A-1 in T1a - T5<br>young WT vs young APP-KI mice<br>(correct + error sessions)	1000 bootstraps compared between young WT mice vs young APP-KI mice	Distribution of the difference significantly different from zero	T1a: p=0.023		
					T1b: p=0.072		
					T1c: p=8.7e-4		
					T2: p=6.6e-7		
					T3: p=1.7e-5		
					T4: p=8.7e-6		
					T5: p=1.1e-3		
**Fig. 3**							
A beta density<br>VTA vsSNc	3b	% Amyloid bata density in the VTA and SNc:<br>APP-KI mice	Young APP-KI mice: 12 mice	Wilcoxon signed-rank test	p=0.0014, Z=-3.2		
TH+ fiber density-LEC	3b	% TH+ fiber density in the LEC:<br>WT mice vs APP-KI mice	young WT:8 mice<br>young APP-KI: 8 mice<br>old WT: 6mice<br>old APP-KI: 6 mice	2-way ANOVA<br>(Strain [WT vs APP-KI] x Age [young vs old])	Strain: F(1,24)=0.79; p=0.38<br>Age: F(1,24)=0.57; p=0.46<br>Strain x Age: F(1,24)=0.22; p=0.22	Tukey test	NS for all pairs
**Fig. 4**							
Dopamine Photometry<br>(correct + error sessions)	4d	Significance of Ca2+ singal in 2-4 s priod	young WT: 10 hemispheres<br>young APP-KI: 8 hemispheres	Wilcoxon signed-rank test	p<0.05	For each strain	
Dopamine Photometry young WT vs young APP-KI mice (correct + error sessions)	4d	GCaMP signals at Odor A	young WT: 10 hemispheres<br>young APP-KI: 8 hemispheres	2-way ANOVA<br>(Strain [young WT vs young APP-KI] x Timepoint [T1, …, T5])	Strain: F(1,80)=0.19; p=0.66<br>Time: F(4,80)=0.33; p=0.85<br>Strain x Time: F(4,80)=0.45; p=0.77	Tukey test	NS for all pairs
		GCaMP signals at Odor B			Strain: F(1,80)=0.41; p=0.52<br>Time: F(4,80)=0.46; p=0.75<br>Strain x Time: F(4,80)=0.69; p=0.59		NS for all pairs
		GCaMP signals at Odor 1			Strain: F(1,80)=9.3; p=3.1e-3<br>Time: F(4,80)=0.67; p=0.61<br>Strain x Time: F(4,80)=2.5; p=0.047		T1 WT vs T5 WT: p=0.048<br>T1 WT vs T1 APP: p=4.2e-3<br>T1 WT vs T2 APP: p=0.0343<br>T1 WT vs T5 APP: p=0.037<br>NS for other pairs
		GCaMP signals at Odor 2			Strain: F(1,80)=11.77; p=9.5e-4<br>Time: F(4,80)=1.26; p=0.29<br>Strain x Time: F(4,80)=1.14; p=0.34		NS for other pairs
Dopamine Photometry young WT correct vs young AD correct	4e, S6c	GCaMP signals at Odor A	young WT: 10 hemispheres<br>young APP-KI: 8 hemispheres	2-way ANOVA<br>(Strain [young WT vs young APP-KI] x Timepoint [T1, …, T5])	Strain: F(1,80)=0.070; p=0.79<br>Time: F(4,80)=0.13; p=0.96<br>Strain x Time: F(4,80)=0.66; p=0.62	Tukey test	NS for all pairs
		GCaMP signals at Odor B			Strain: F(1,80)=0.17; p=0.67<br>Time: F(4,80)=0.17; p=0.94<br>Strain x Time: F(4,80)=1.07; p=0.37		NS for all pairs
		GCaMP signals at Odor 1			Strain: F(1,80)=1.68; p=0.011<br>Time: F(4,80)=0.59; p=0.67<br>Strain x Time: F(4,80)=3.72; p=7.8e-3		T1 WT correct vs T5 WT correct: p=0.016<br>T1 WT correct vs T1 AD correct: p=4.8e-3<br>NS for other pairs
		GCaMP signals at Odor 2			Strain: F(1,80)=11.2; p=1.2e-3<br>Time: F(4,80)=0.98; p=0.42<br>Strain x Time: F(4,80)=2.06; p=0.093		T1 WT correct vs T1 AD correct: p=0.010<br>NS for other pairs
Dopamine Photometry young WT error vs young AD error	4f, S6c	GCaMP signals at Odor A	young WT: 10 hemispheres<br>young APP-KI: 8 hemispheres	2-way ANOVA<br>(Strain [young WT vs young APP-KI] x Timepoint [T1, …, T5])	Strain: F(1,80)=1.03; p=0.31<br>Time: F(4,80)=0.34; p=0.84<br>Strain x Time: F(4,80)=0.062; p=0.99	Tukey test	NS for all pairs
		GCaMP signals at Odor B			Strain: F(1,80)=0.17; p=0.67<br>Time: F(4,80)=0.12; p=0.97<br>Strain x Time: F(4,80)=0.25; p=0.91		NS for all pairs
		GCaMP signals at Odor 1			Strain: F(1,80)=4.05; p=0.047<br>Time: F(4,80)=1.06; p=0.38<br>Strain x Time: F(4,80)=0.55; p=0.69		NS for all pairs
		GCaMP signals at Odor 2			Strain: F(1,80)=7.91; p=0.0061<br>Time: F(4,80)=1.09; p=0.36<br>Strain x Time: F(4,80)=0.86; p=0.49		NS for all pairs
**Fig. 5**							
Behavior-DA fiber stimulation	5c	% Correct Trials in T5:<br>Control vs Stimulation	Young APP-KI: 11 mice	2-way ANOVA<br>(Odor type [A/B vs 1/2] x Stim [Control vs Stimulation])	Odor: F(1,40)=17.26; p=1.6e-4<br>Stim: F(1,40)=4.73; p=0.0356<br>Odor x Stim: F(1,40)=4.17; p=0.047	Tukey test	A/B Ctr vs 1/2 Ctr: p=4.6e-4<br>1/2 Ctr vs A/B Stim: p=3.4e-4<br>1/2 Ctr vs 1/2 Stim: p=0.024<br>NS for other pairs
	5d	% Correct Sessions in T5:<br>Control vs Stimulation	Young APP-KI: 11 mice	Unpaired t test	t(20) = -2.55, p = 0.019		

Supplementary Table 1. Statistics used in this study (continued)

**Fig. 6**							
Behavior-L-dopa treatment	6b	% Correct Trials in T5:<br>Saline vs L-dopa	Saline:8 mice<br>L-dopa: 9 mice	2-way ANOVA<br>(Odor type [A/B vs 1/2] x Drug type [Saline vs L-dopa])	Odor: F(1,30)=37.3; p=1.1e-6<br>Drug: F(1,30)=9.1e-4; p=9.1e-4<br>Odor x Drug: F(1,30)=3.6; p=0.029	Tukey test	A/B Saline vs 1/2 Saline: p=1.5e-5<br>A/B Saline vs A/B L-dopa: p=6.3e-7<br>1/2 Saline vs 1/2 L-dopa: p=1.1e-3<br>A/B L-dopa vs 1/2 L-dopa: p=1.5e-5<br><br>NS for other pairs
	6c	% Correct Strains in T5:<br>Saline vs L-dopa	Saline:8 mice<br>L-dopa: 9 mice	Unpaired t test	t(15) = -4.25, p = 7.0e-4		
LEC cells recording (Saline treatment, correct + error Session)	6e	T1	Saline: 132 cells	1000 Shuffling<br>(trial shift)	95th percentile value = 0.73		
		T3			95th percentile value = 0.71		
		T5			95th percentile value = 0.73		
LEC cells recording (L-dopa treatment, correct + error Session)	6f	T1	L-dopa: 132 cells	1000 Shuffling<br>(trial shift)	95th percentile value = 0.98		
		T3			95th percentile value = 0.99		
		T5			95th percentile value = 0.93		
Bootstrapping analyses for Similarity Index	6i, S10	Similarity Index for A-1 in T1a - T5<br>Saline vs L-dopa treatment<br>(correct + error Strains)	1000 bootstraps compared between Saline vs L-dopa treatment	Distribution of the difference significantly different from zero	T1a: p=0.21		
					T1b: p=0.18		
					T1c: p=9.1e-5		
					T2: p=4.8e-3		
					T3: p=0.011		
					T4: p=0.041		
					T5: p=0.12		
Behavior-L-dopa treatment in PS19 mice	6k, S10	% Correct Trials in T5:<br>Saline vs L-dopa	Saline: 5 mice<br>L-dopa: 5 mice	2-way ANOVA<br>(Odor type [A/B vs 1/2] x Drug [Saline vs L-dopa])	Odor: F(1,16)=24.96; p=1.3e-4<br>Drug: F(1,16)=5.8; p=0.028<br>Odor x Drug: F(1,16)=10.99; p=0.0044	Tukey test	A/B Sal vs 1/2 Sal: p=1.2e-4<br>1/2 Sal vs A/B dopa: p=1.2e-4<br>1/2 Sal vs 1/2 dopa: p=0.0047<br>NS for other pairs
	6l, S10	% Correct Sessions in T5:<br>Saline vs L-dopa		Unpaired t test	t(8) = -3.18, p = 8.9e-3		

**Extended Data Fig. 1**							
A beta density-LEC	E1b	% Amyloid bata density in the LEC:<br>WT mice vs APP-KI mice	12 mo WT:4 mice<br>1 mo APP-KI: 4 mice<br>2mo APP-KI: 4 mice<br>3 mo APP-KI: 4 mice<br>4 mo APP-KI: 4 mice<br>6 mo APP-KI: 4 mice<br>9 mo APP-KI: 4 mice<br>12 mo APP-KI: 4 mice	1-way ANOVA<br>(Group [12 mo WT vs 1 mo APP vs 2 mo APP vs 3 mo APP vs 4 mo APP vs 6 mo APP vs 9 mo APP vs12 mo APP])	Group: F(7,24)=15.34; p=1.8e-7	Tukey test	12 mo WT vs 12 mo APP: p=4.1e-7<br>1 mo APP vs 12 mo APP: p=4.1e-7<br>2 mo APP vs 12 mo APPI: p=6.2e-7<br>3 mo APP vs 12 mo APP: p=1.4e-6<br>4 mo APP vs 12 mo APP: p=1.6e-5<br>6 mo APP vs 12 mo APP: p=2.3e-4<br>9 mo APP vs 12 mo APP: p=2.6e-5<br>NS for other pairs
A beta density-VTA	E1c	% Amyloid bata density in the VTA:<br>WT mice vs APP-KI mice	12 mo WT:4 mice<br>1 mo APP-KI: 4 mice<br>2mo APP-KI: 4 mice<br>3 mo APP-KI: 4 mice<br>4 mo APP-KI: 4 mice<br>6 mo APP-KI: 4 mice<br>9 mo APP-KI: 4 mice<br>12 mo APP-KI: 4 mice	1-way ANOVA<br>(Group [12 mo WT vs 1 mo APP vs 2 mo APP vs 3 mo APP vs 4 mo APP vs 6 mo APP vs 9 mo APP vs12 mo APP])	Group: F(7,24)=13.18; p=7.4e-7	Tukey test	12 mo WT vs 12 mo APP: p=6.6e-4<br>1 mo APP vs 9 mo APP: p=0.038<br>1 mo APP vs 12 mo APPI: p=1.9e-4<br>2 mo APP vs 12 mo APP: p=5.3e-4<br>3 mo APP vs 12 mo APP: p=0.022<br>4 mo APP vs 12 mo APP: p=0.011<br>NS for other pairs
A beta density-SNc	E1c	% Amyloid bata density in the SNc:<br>WT mice vs APP-KI mice	12 mo WT:4 mice<br>1 mo APP-KI: 4 mice<br>2mo APP-KI: 4 mice<br>3 mo APP-KI: 4 mice<br>4 mo APP-KI: 4 mice<br>6 mo APP-KI: 4 mice<br>9 mo APP-KI: 4 mice<br>12 mo APP-KI: 4 mice	1-way ANOVA<br>(Group [12 mo WT vs 1 mo APP vs 2 mo APP vs 3 mo APP vs 4 mo APP vs 6 mo APP vs 9 mo APP vs12 mo APP])	Group: F(7,24)=13.18; p=7.4e-7	Tukey test	12 mo WT vs 6 mo APP: p=0.035<br>12 mo WT vs 9 mo APP: p=0.024<br>12 mo WT vs 12 mo APP: p=3.7e-6<br>1 mo APP vs 6 mo APP: p=0.029<br>1 mo APP vs 9 mo APP: p=0.020<br>1 mo APP vs 12 mo APP: p=3.1e-6<br>2 mo APPI vs 9 mo APP: p=0.035<br>2 mo APP vs 12 mo APP: p=5.5e-6<br>3 mo APP vs 12 mo APP: p=2.8e-5<br>4 mo APP vs 12 mo APP: p=1.3e-4<br>6 mo APP vs 12 mo APP: p=0.015<br>9 mo APP vs 12 mo APP: p=0.026<br>NS for other pairs

**Extended Data Fig. 3**							
LEC cells:<br>Distribution of Response Types between young WT and young APP-KI mice	E3c	T1	5 mice (young WT mice) vs<br>5 mice (young APP-KI mice)	2-way ANOVA<br>(responsive type [1 odor, 2 odor, 3 odor, 4 odor, non] x Strain [WT, AD])	Type: F(4,40)=359.1; p=9.2e-31<br>Strain: F(1,40)=0; p=1<br>Type x Strain: F(4,40)=2.73; p=0.043	Tukey test	1 odor WT vs 2 odor WT : p = 9.6e-5<br>1 odor WT vs 3 odor WT : p = 7.7e-6<br>1 odor WT vs 4 odor WT : p = 9.4e-7<br>1 odor WT vs non WT : p = 1.3e-18<br>1 odor WT vs 2 odor APP : p = 6.8e-4<br>1 odor WT vs 3 odor APP : p = 3.4e-5<br>1 odor WT vs 4 odor APP : p = 1.0e-6<br>1 odor WT vs non APP : p = 9.1e-16<br>2 odor WT vs non WT : p = 5.8e-25<br>2 odor WT vs 1 odor APP : p = 5.2e-7<br>2 odor WT vs non APP : p = 1.4e-21<br>3 odor WT vs non WT : p = 5.0e-26<br>3 odor WT vs 1 odor APP : p = 4.2e-8<br>3 odor WT vs non APP : p = 1.6e-22<br>4 odor WT vs non WT : p = 5.9e-27<br>4 odor WT vs 1 odor APP : p = 5.6e-9<br>4 odor WT vs non APP : p = 2.7e-23<br>non WT vs 1 odor APP : p = 6.3e-17<br>non WT vs 2 odor APP : p = 3.8e-24<br>non WT vs 3 odor APP : p = 2.1e-25<br>non WT vs 4 odor APP : p = 6.4e-27<br>1 odor APP vs 2 odor APP : p = 3.8e-6<br>1 odor APP vs 3 odor APP : p = 1.8e-7<br>1 odor APP vs 4 odor APP : p = 6.0e-9<br>1 odor APP vs non APP : p = 5.3e-14<br>2 odor APP vs non APP : p = 6.9e-21<br>3 odor APP vs non APP : p = 5.7e-22<br>4 odor APP vs non APP : p = 2.8e-23<br>NS for other pairs
		T3			Type: F(4,40)=255.02; p=6.9e-28<br>Strain: F(1,40)=0; p=1<br>Type x Strain: F(4,40)=2.51; p=0.057		1 odor WT vs 2 odor WT : p = 2.1e-2<br>1 odor WT vs 3 odor WT : p = 2.4e-3<br>1 odor WT vs 4 odor WT : p = 1.9e-3<br>1 odor WT vs non WT : p = 3.4e-17<br>1 odor WT vs 3 odor APP : p = 4.6e-3<br>1 odor WT vs 4 odor APP : p = 2.5e-3<br>1 odor WT vs non APP : p = 2.2e-14<br>2 odor WT vs non WT : p = 4.4e-21<br>2 odor WT vs 1 odor APP : p = 3.1e-4<br>2 odor WT vs non APP : p = 3.1e-18<br>3 odor WT vs non WT : p = 5.8e-22<br>3 odor WT vs 1 odor APP : p = 2.7e-5<br>3 odor WT vs non APP : p = 4.8e-19<br>4 odor WT vs non WT : p = 4.6e-22<br>4 odor WT vs 1 odor APP : p = 2.1e-5<br>4 odor WT vs non APP : p = 3.9e-19<br>non WT vs 1 odor APP : p = 1.1e-15<br>non WT vs 2 odor APP : p = 4.3e-20<br>non WT vs 3 odor APP : p = 1.0e-21<br>non WT vs 4 odor APP : p = 6.0e-22<br>1 odor APP vs 2 odor APP : p = 4.7e-3<br>1 odor APP vs 3 odor APP : p = 5.4e-5<br>1 odor APP vs 4 odor APP : p = 2.8e-5<br>1 odor APP vs non APP : p = 9.4e-13<br>2 odor APP vs non APP : p = 2.6e-17<br>3 odor APP vs non APP : p = 8.1e-19<br>4 odor APP vs non APP : p = 5.0e-19<br>NS for other pairs

Supplementary Table 1. Statistics used in this study (continued)

**Extended Data Fig. 3 (continuted)**							
LEC cells:<br>Distribution of Response Types between young WT and young APP-KI mice	E3c	T5	5 mice (young WT mice) vs<br>5 mice (young APP-KI mice)	2-way ANOVA<br>(responsive type [1 odor, 2 odor, 3 odor, 4 odor, non] x Strain [WT, AD])	Type: F(4,40)=351.51; p=1.4e-30<br>Strain: F(1,40)=0; p=1<br>Type x Strain: F(4,40)=8.01; p=7.7e-5	Tukey test	1 odor WT vs 2 odor WT : p = 2.0e-2<br>1 odor WT vs 3 odor WT : p = 1.0e-2<br>1 odor WT vs 4 odor WT : p = 2.4e-3<br>1 odor WT vs non WT : p = 1.5e-21<br>1 odor WT vs 1 odor APP : p = 2.1e-2<br>1 odor WT vs 3 odor APP : p = 7.5e-3<br>1 odor WT vs 4 odor APP : p = 3.0e-3<br>1 odor WT vs non APP : p = 5.0e-17<br>2 odor WT vs non WT : p = 3.3e-26<br>2 odor WT vs 1 odor APP : p = 2.3e-7<br>2 odor WT vs non APP : p = 6.1e-21<br>3 odor WT vs non WT : p = 1.5e-26<br>3 odor WT vs 1 odor APP : p = 1.0e-7<br>3 odor WT vs non APP : p = 3.2e-21<br>4 odor WT vs non WT : p = 3.0e-27<br>4 odor WT vs 1 odor APP : p = 2.3e-8<br>4 odor WT vs non APP : p = 8.9e-22<br>non WT vs 1 odor APP : p = 1.3e-17<br>non WT vs 2 odor APP : p = 2.2e-25<br>non WT vs 3 odor APP : p = 1.1e-26<br>non WT vs 4 odor APP : p = 3.7e-27<br>non WT vs non APP : p = 4.3e-3<br>1 odor APP vs 2 odor APP : p = 1.6e-6<br>1 odor APP vs 3 odor APP : p = 7.6e-8<br>1 odor APP vs 4 odor APP : p = 2.8e-8<br>1 odor APP vs non APP : p = 4.6e-13<br>2 odor APP vs non APP : p = 2.8e-20<br>3 odor APP vs non APP : p = 2.5e-21<br>4 odor APP vs non APP : p = 1.1e-21<br>NS for other pairs
LEC cells recording (young WT mice, correct + error sessions)	E3d	T1	5 mice (young WT mice) vs<br>5 mice (young APP-KI mice)			Unpaired t-test followed by FDR correction (Benjamini –Hochberg).	NS
		T3					NS
		T5					A-1 WT vs A-1 APP: p = 0.048
Similarity Index	E3e	Similarity Index for A-1 in T1a - T5<br>young WT vs young APP-KI mice<br>(correct + error sessions)	young WT mice: n = 5 mice young APP-KI mice: n = 5 mice	2-way ANOVA<br>(Strain [WT vs APP] x Timepoint [T1a, …, T5])	Strain: F(1,56)=14.77; p=3.1e-4<br>Time: F(6,56)=0.47; p=0.82<br>Strain x Time: F(6,56)=0.38; p=0.87	Tukey test	NS

**Extended Data Fig. 4**							
LEC cells:<br>Distribution of Spike Types	E4a	Distribution of FS, RS, WS cells<br>in young WT and young APP-KI mice	Young WT: 479 cells<br>Young APP-KI:846 cells	Chi-Square test	p=1.2e-23, x^2=105.51	Benjamini-Hochberg post-hoc test	NS: p=7.3e-9<br>RS: p=1.0e-4<br>WS: p<0.00001
LEC cells:Comparison of Spike width between young WT and young APP-KI mice	E4b	Spike width:Putative principal neurons	Young WT:423 cells<br>Young APP-KI: 636 cells	Wilcoxon rank sum test	p = 1.7e-30, Z = 11.47		
		Spike width: Putative interneurons	Young WT:56 cells<br>Young APP-KI: 210 cells		p = 0.76, Z = -0.29		
LEC cells:Comparison of Firing rate between young WT and young APP-KI mice	E4c	Mean firing rate: Putative principal neurons	Young WT:423 cells<br>Young APP-KI: 636 cells	Wilcoxon rank sum test	p = 3.9e-15, Z = -7.85		
				Kolmogorov–Smirnov test	p=3.3e-12		
		Peak firing rate: Putative principal neurons		Wilcoxon rank sum test	p = 4.7e-5, Z = -4.06		
				Kolmogorov–Smirnov test	p=4.4e-4		
LEC cells:Comparison of Firing rate between young WT and young APP-KI mice	E4d	Mean firing rate: Putative interneurons	Young WT:56 cells<br>Young APP-KI: 210 cells	Wilcoxon rank sum test	p = 0.0044, Z = -2.84		
				Kolmogorov–Smirnov test	p=0.031		
		Peak firing rate: Putative interneurons		Wilcoxon rank sum test	p = 0.75, Z = 0.31		
				Kolmogorov–Smirnov test	p=0.81		

**Extended Data Fig. 5**							
LEC cells:<br>Distribution of Response Types between young WT and young APP-KI mice	E5a	T1	Young WT:423 cells<br>Young APP-KI: 636 cells	Chi-Square test	p=5.5e-4, x^2=39.39	Benjamini-Hochberg post-hoc test	A: p=0.032<br>A-1: p=0.032<br>null responsive type: p=1.4e-5<br>NS for other pairs
		T3			p=4.5e-7, x^2=58.49		A: p=3.8e-3<br>1: p=0.014<br>A-1: p=0.026<br>B-1: p=0.026<br>null responsive type: p=3.1e-8<br>NS for other pairs
		T5			p=6.4e-6, x^2=51.63		A: p=1.8e-3<br>1: p=0.048<br>A-1: p=0.048<br>B-1: p=0.048<br>null responsive type: p=8.2e-10<br>NS for other pairs

**Extended Data Fig. 6**							
LEC cells recording	E6a, E6b, E6c, E6d, E6e, E6f, E6g, E6h	Significance of LEC cell firing in odor period	10 trials	Wilcoxon signed-rank test	p<0.05	For each LEC cell	

**Extended Data Fig. 7**							
LEC cells recording (young WT mice, correct session)	E7a	AB-only	Young WT: 352 cells	1000 Shuffling<br>(trial shift)	95th percentile value = 1.33		
		T1			95th percentile value = 1.42		
		T2			95th percentile value = 1.35		
		T3			95th percentile value = 1.36		
		T4			95th percentile value = 1.41		
		T5			95th percentile value = 1.43		
LEC cells recording (young WT mice, error session)		AB-only	Young WT: 71 cells	1000 Shuffling<br>(trial shift)	95th percentile value = 0.69		
		T1			95th percentile value = 0.69		
		T2			95th percentile value = 0.71		
		T3			95th percentile value = 0.71		
		T4			95th percentile value = 0.73		
		T5			95th percentile value = 0.76		

Supplementary Table 1. Statistics used in this study (continued)

**Extended Data Fig. 7 (continued)**							
Bootstrapping analyses for Decoding performance Young WT correct vs Young WT error	E7b	Decoding performance in T1a - T5 young WT correct vs young WT error	10 bootstraps compared between young WT correct vs young WT error	2-way ANOVA (Session [correct vs error] x Timepoint [T1a, …, T5])	Session: F(1,126)=372.1; p=2.0e-39<br>Time: F(6,126)=2.60; p=0.021<br>Session x Time: F(6,126)=37.83; p=5.7e-26	Tukey test	T1a correct WT vs T2 correct WT : p = 7.1e-5<br>T1a correct WT vs T2 error WT : p = 1.2e-2<br>T1a correct WT vs T3 correct WT : p = 7.8e-13<br>T1a correct WT vs T3 error WT : p = 1.7e-4<br>T1a correct WT vs T4 correct WT : p = 2.5e-20<br>T1a correct WT vs T4 error WT : p = 1.2e-6<br>T1a correct WT vs T5 correct WT : p = 2.2e-10<br>T1a correct WT vs T5 error WT : p = 8.7e-4<br>T1a error WT vs T2 correct WT : p = 8.4e-3<br>T1a error WT vs T2 error WT : p = 1.2e-4<br>T1a error WT vs T3 correct WT : p = 3.2e-9<br>T1a error WT vs T3 error WT : p = 5.2e-7<br>T1a error WT vs T4 correct WT : p = 2.7e-15<br>T1a error WT vs T4 error WT : p = 1.1e-9<br>T1a error WT vs T5 correct WT : p = 3.2e-7<br>T1a error WT vs T5 error WT : p = 3.9e-6<br>T1b correct WT vs T2 correct WT : p = 1.1e-4<br>T1b correct WT vs T2 error WT : p = 8.9e-3<br>T1b correct WT vs T3 correct WT : p = 1.6e-12<br>T1b correct WT vs T3 error WT : p = 1.1e-4<br>T1b correct WT vs T4 correct WT : p = 6.9e-20<br>T1b correct WT vs T4 error WT : p = 7.1e-7<br>T1b correct WT vs T5 correct WT : p = 4.2e-10<br>T1b correct WT vs T5 error WT : p = 5.9e-4<br>T1b error WT vs 1c correct WT : p = 1.1e-3<br>T1b error WT vs T2 correct WT : p = 1.9e-7<br>T1b error WT vs T3 correct WT : p = 3.5e-17<br>T1b error WT vs T3 error WT : p = 1.5e-2<br>T1b error WT vs T4 correct WT : p ≈ 0<br>T1b error WT vs T4 error WT : p = 3.2e-4<br>T1b error WT vs T5 correct WT : p = 3.4e-14<br>T1b error WT vs T5 error WT : p = 4.9e-2<br>T1c correct WT vs 1c error WT : p = 3.5e-2<br>T1c correct WT vs T2 error WT : p = 9.1e-11<br>T1c correct WT vs T3 correct WT : p = 1.2e-3<br>T1c correct WT vs T3 error WT : p = 2.0e-14<br>T1c correct WT vs T4 correct WT : p = 1.4e-7<br>T1c correct WT vs T4 error WT : p = 1.7e-18<br>T1c correct WT vs T5 correct WT : p = 1.9e-2<br>T1c correct WT vs T5 error WT : p = 4.5e-13<br>T1c error WT vs T2 correct WT : p = 3.3e-5<br>T1c error WT vs T2 error WT : p = 2.1e-2<br>T1c error WT vs T3 correct WT : p = 2.1e-13<br>T1c error WT vs T3 error WT : p = 3.6e-4<br>T1c error WT vs T4 correct WT : p = 3.4e-21<br>T1c error WT vs T4 error WT : p = 2.8e-6<br>T1c error WT vs T5 correct WT : p = 7.0e-11<br>T1c error WT vs T5 error WT : p = 1.7e-3<br>T2 correct WT vs T2 error WT : p = 4.1e-17<br>T2 correct WT vs T3 error WT : p ≈ 0<br>T2 correct WT vs T4 correct WT : p = 8.5e-4<br>T2 correct WT vs T4 error WT : p ≈ 0
LEC cells recording (young APP-KI mice, correct session)	E7c	AB-only	Young APP-KI: 411 cells	1000 Shuffling (trial shift)	95th percentile value = 1.57		
		T1			95th percentile value = 1.74		
		T2			95th percentile value = 1.65		
		T3			95th percentile value = 1.69		
		T4			95th percentile value = 1.72		
		T5			95th percentile value = 1.71		
LEC cells recording (young APP-KI mice, error session)		AB-only	Young APP-KI: 225 cells	1000 Shuffling (trial shift)	95th percentile value = 1.15		
		T1			95th percentile value = 1.31		
		T2			95th percentile value = 1.41		
		T3			95th percentile value = 1.36		
		T4			95th percentile value = 1.31		
		T5			95th percentile value = 1.38		
Bootstrapping analyses for Decoding performance Young AD correct vs Young AD error	E7d	Decoding performance in T1a - T5 young WT correct vs young WT error	10 bootstraps compared between young APP-KI correct vs young APP-KI error	2-way ANOVA (Session [correct vs error] x Timepoint [T1a, …, T5])	Session: F(1,126)=141.47; p=2.4e-22<br>Time: F(6,126)=4.29; p=5.7e-4<br>Session x Time: F(6,126)=21.59; p=2.4e-17	Tukey test	T1a correct APP vs T2 correct APP: p = 1.3e-3<br>T1a correct APP vs T3 correct APP: p = 1.4e-5<br>T1a correct APP vs T3 error APP: p = 2.7e-2<br>T1a correct APP vs T4 correct APP: p = 3.9e-8<br>T1a correct APP vs T5 correct APP: p = 3.4e-5<br>T1a correct APP vs T5 error APP: p = 3.1e-3<br>T1a error APP vs T2 correct APP: p = 8.2e-4<br>T1a error APP vs T3 correct APP: p = 7.7e-6<br>T1a error APP vs T3 error APP: p = 4.0e-2<br>T1a error APP vs T4 correct APP: p = 1.9e-8<br>T1a error APP vs T5 correct APP: p = 1.9e-5<br>T1a error APP vs T5 error APP: p = 4.9e-3<br>T1b correct APP vs T2 correct APP: p = 6.9e-4<br>T1b correct APP vs T3 correct APP: p = 6.2e-6<br>T1b correct APP vs T3 error APP: p = 4.5e-2<br>T1b correct APP vs T4 correct APP: p = 1.4e-8<br>T1b correct APP vs T5 correct APP: p = 1.6e-5<br>T1b correct APP vs T5 error APP: p = 5.7e-3<br>T1b error APP vs T2 correct APP: p = 4.9e-2<br>T1b error APP vs T3 correct APP: p = 1.5e-3<br>T1b error APP vs T3 error APP: p = 6.0e-4<br>T1b error APP vs T4 correct APP: p = 1.2e-5<br>T1b error APP vs T4 error APP: p = 4.3e-2<br>T1b error APP vs T5 correct APP: p = 3.0e-3<br>T1b error APP vs T5 error APP: p = 3.6e-5<br>T1c correct APP vs T2 correct APP: p = 2.4e-6<br>T1c correct APP vs T3 correct APP: p = 6.7e-9<br>T1c correct APP vs T4 correct APP: p = 3.7e-12<br>T1c correct APP vs T5 correct: APP p = 2.1e-8<br>T1c error APP vs T2 correct APP: p = 1.6e-6<br>T1c error APP vs T3 correct APP: p = 4.0e-9<br>T1c error APP vs T4 correct APP: p = 2.0e-12<br>T1c error APP vs T5 correct APP: p = 1.3e-8<br>T2 correct APP vs T2 error APP: p = 7.7e-7<br>T2 correct APP vs T3 error APP: p = 1.5e-13<br>T2 correct APP vs T4 error APP: p = 9.8e-10<br>T2 correct APP vs T5 error APP: p = 6.6e-16<br>T2 error APP vs T3 correct APP: p = 1.7e-9<br>T2 error APP vs T4 correct APP: p = 7.3e-13<br>T2 error APP vs T5 correct APP: p = 5.7e-9<br>T3 correct APP vs T3 error APP: p = 1.7e-17<br>T3 correct APP vs T4 error APP: p = 6.0e-13<br>T3 correct APP vs T5 error APP: p = 2.4e-20<br>T3 error APP vs T4 correct APP: p ≈ 0<br>T3 error APP vs T5 correct APP: p = 9.8e-17<br>T4 correct APP vs T4 error APP: p = 4.9e-17<br>T4 correct APP vs T5 error: APP: p ≈ 0<br>T4 error APP vs T5 correct APP: p = 2.5e-12<br>T5 correct APP vs T5 error APP: p = 1.9e-19<br>NS for other pairs"

Supplementary Table 1. Statistics used in this study (continued)

**Extended Data Fig. 8**							
LEC cells recording (young WT mice, correct + error sessions)	E8a	T1a	423 cells	1000 Shuffling<br>(trial shift)	95th percentile value = 1.16		
		T1b			95th percentile value = 1.14		
		T1c			95th percentile value = 1.11		
LEC cells recording (young APP-KI mice, correct + error sessions)	E8a	T1a	636 cells	1000 Shuffling<br>(trial shift)	95th percentile value = 1.67		
		T1b			95th percentile value = 1.61		
		T1c			95th percentile value = 1.64		
**Extended Data Fig. 9**							
LEC cells recording	E9a, E9b	Significance of LEC cell firing in odor period	10 trials	Wilcoxon signed-rank test	p<0.05	For each LEC cell	
LEC cells recording (old WT mice, correct + error sessions)	S2a	AB-only	671 cells	1000 Shuffling<br>(trial shift)	95th percentile value = 1.22		
	E9e, S2a	T1			95th percentile value = 2.41		
	S2a	T2			95th percentile value = 2.42		
	E9e, S2a	T3			95th percentile value = 2.41		
	S2a	T4			95th percentile value = 2.34		
	E9e, S2a	T5			95th percentile value = 2.33		
LEC cells recording (old APP-KI mice, correct + error sessions)	S2b	AB-only	618 cells	1000 Shuffling<br>(trial shift)	95th percentile value = 1.23		
	E9e, S2b	T1			95th percentile value = 2.09		
	S2b	T2			95th percentile value = 2.03		
	E9e, S2b	T3			95th percentile value = 1.93		
	S2b	T4			95th percentile value = 2.00		
	E9e, S2b	T5			95th percentile value = 1.91		
Bootstrapping analyses for Similarity Index	E9g	Similarity Index for A-1 in T1 - T5<br>young WT vs young APP-KI mice	1000 bootstraps compared between young WT mice vs young APP-KI mice	Distribution of the difference significantly different from zero	T1a: p=0.17		
					T1b: p=0.0059		
					T1c: p=7.1e-6		
					T2: p=0.015		
					T3: p=0.089		
					T4: p=0.017		
					T5: p=0.0069		
**Extended Data Fig. 10**							
TH+ fiber density-VTA	E10b	% TH+ fiber density in the VTA:<br>WT mice vs APP-KI mice	young WT:8 mice<br>young APP-KI: 8 mice<br>old WT: 6mice<br>old APP-KI: 6 mice	2-way ANOVA<br>(Strain [WT vs APP-KI] x Age [young vs old])	Strain: F(1,22)=0.01; p=0.91<br>Age: F(1,22)=10.0; p=0.0045<br>Strain x Age: F(1,22)=0.080; p=0.78	Tukey test	NS for all pairs
TH+ fiber density-SNc	E10b	% TH+ fiber density in the VTA:<br>WT mice vs APP-KI mice	young WT:8 mice<br>young APP-KI: 8 mice<br>old WT: 6mice<br>old APP-KI: 6 mice	2-way ANOVA<br>(Strain [WT vs APP-KI] x Age [young vs old])	Strain: F(1,22)=0.41; p=0.52<br>Age: F(1,22)=12.95; p=0.0016<br>Strain x Age: F(1,22)=0.20; p=0.65	Tukey test	young WT vs old AD: p=0.038
**Supplementary Fig. 1**							
Behavior-Young WT mice vs Young APP-KI mice	S1a	% Correct Trials in T5:<br>WT mice vs APP-KI mice<br>(Odor A vs B vs 1 vs 2)	Young WT:11 mice<br>Young APP-KI: 16 mice	2-way ANOVA<br>(Odor type [A vs B vs 1 vs 2] x Strain [WT vs APP-KI])	Odor: F(3,100)=35.46; p=1.1e-15<br>Strain: F(1,100)=5.5; p=0.021<br>Odor x Strain: F(3,100)=9.88; p=9.1e-6	Tukey test	A WT vs B WT: p=0.021<br>A WT vs B APP: p=0.019<br>A WT vs 1 APP: p=3.5e-7<br>B WT vs 1 WT: p=2.6e-3<br>B WT vs 1 APP: p=3.8e-15<br>1 WT vs B APP: p=1.9e-3<br>1 WT vs 1 APP: p=8.1e-6<br>2 WT vs 1 APP: p=7.1e-12<br>A APP vs 1 APP: p=2.4e-13<br>B APP vs 1 APP: p=6.9e-17<br>1 APP vs 2 APP: p=1.3e-11<br>NS for other pairs
Behavior-Old WT mice vs Old APP-KI mice	S1b	% Correct Trials in T5:<br>WT mice vs APP-KI mice	Old WT:11 mice<br>Old APP-KI: 14 mice	2-way ANOVA<br>(Odor type [A vs B vs 1 vs 2] x Strain [WT vs APP-KI])	Odor: F(3,92)=97.01; p=2.2e-28<br>Strain: F(1,92)=17.35; p=6.9e-5<br>Odor x Stain: F(3,92)=30.11; p=1.2e-13	Tukey test	A WT vs 1 WT: p=0.024<br>A WT vs 1 APP: p=5.3e-28<br>B WT vs 1 WT: p=6.1e-6<br>B WT vs 1 APP: p=7.3e-28<br>1 WT vs A APP: p=5.5e-3<br>1 WT vs B APP: p=1.2e-6<br>1 WT vs 1 APP: p=1.5e-15<br>1 WT vs 2 APP: p=2.3e-4<br>2 WT vs 1 APP: p=4.4e-22<br>A APP vs 1 APP: p=1.8e-25<br>B APP vs 1 APP: p=2.7e-30<br>1 APP vs 2 APP: p=2.2e-27<br>NS for other pairs
Behavior-Young WT mice vs Old WT mice	S1c	% Correct Trials in T5:<br>young WT mice vs old WT mice	Young WT:11 mice<br>Old WT: 11 mice	2-way ANOVA<br>(Odor type [A/B vs 1/2] x Age [young vs old])	Odor: F(1,40)=5.02; p=0.031<br>Age: F(1,40)=2.91; p=0.095<br>Odor x Age: F(1,40)=5.04; p=0.031	Tukey test	A/B young vs 1/2 young: p=0.038<br>1/2 young vs 1/2 old: p=0.038<br>A/B old vs 1/2 old: p=0.014<br>NS for other pairs
	S1d	% Correct Sessions in T5:<br>young WT mice vs old WT mice	Young WT: 49 sessions<br>Old WT: 100 sessions	Binomial test	p=0.53		
	S1e	% Correct Trials in T5:<br>young WT mice vs old WT mice	Young WT:11 mice<br>Old WT: 11 mice	2-way ANOVA<br>(Odor type [A vs B vs 1 vs 2] x Age [young vs old])	Odor: F(3,80)=22.53; p=1.1e-10<br>Strain: F(1,80)=0.10; p=0.74<br>Odor x Stain: F(3,80)=2.25; p=0.088	Tukey test	NS for all pairs
Days of pre-learing	S1f	Days for pre-learning:<br>WT vs APP-KI	Young WT:11 mice<br>Young APP-KI: 16 mice<br>Old WT:11 mice<br>Old APP-KI: 14 mice	2-way ANOVA<br>(Strain [WT vs APP-KI] x Age [young vs old])	Strain: F(1,48)=0.01; p=0.0141<br>Age: F(1,48)=3.0e-4; p=0.95<br>Strain x Age: F(1,48)=0.0022; p=0.96	Tukey test	NS for all pairs
**Supplemantary Fig. 2**							
LEC cells:<br>Distribution of Spike Types	S2a	Distribution of NS, RS, WS cells<br>in young WT and young APP-KI mice	5 mice (young WT mice)<br>5 mice (young APP-KI mice)	2-way ANOVA<br>(Strain [WT vs APP] x Spiketype [FS,RS,WS])	Strain: F(1,24)=0; p=1<br>Spiketype: F(2,24)=35.5; p=6.6e-8<br>Strain x Time: F(2,24)=8.59; p=1.5e-3		FS WT vs RS WT: p = 1.6e-3<br>FS WT vs FS APP: p = 0.032<br>FS WT vs RS APP:p = 6.3e-6<br>RS WT vs WS WT: p = 9.9e-5<br>WS WT vs RS APP: p = 1.2e-3<br>WS WT vs WS APP: p = 0.031<br>FS APP vs RS APP: p = 7.6e-5<br>RS APP vs WS APP: p= 4.8e-7<br>NS for other paris
LEC cells:Comparison of Spike width between young WT and young APP-KI mice	S2b	Spike width:Putative principal cells	5 mice (young WT mice)<br>5 mice (young APP-KI mice)	Unpaired t test	t(8) = 2.49, p = 0.037		
		Spike width: Putative interneurons			t(8) = -0.81, p = 0.44		
LEC cells:Comparison of Firing rate between young WT and young APP-KI mice	S2c	Mean firing rate: Putative principal cells	5 mice (young WT mice)<br>5 mice (young APP-KI mice)	Unpaired t test	t(8) = -4.1, p = 3.5e-3		
		Peak firing rate: Putative principal cells			t(8) = -2.6, p = 0.032		
LEC cells:Comparison of Peak firing rate between young WT and young APP-KI mice	S2d	Mean firing rate: Putative interneurons	5 mice (young WT mice)<br>5 mice (young APP-KI mice)	Unpaired t test	t(8) = -1.8, p = 0.099		
		Peak firing rate: Putative interneurons			t(8) = -4.2, p = 3.1e-3		
**Supplementary Fig. 3**							
LEC cells:<br>Distribution of Response Types between young WT and young APP-KI mice	S3a	T1	5 mice (young WT mice)<br>5 mice (young APP-KI mice)			Unpaired t-test followed by FDR correction (Benjamini –Hochberg).	NS
		T3					NS
		T5					Odor A WT vs Odor A APP: p =0.031
**Supplementary Fig. 6**							
Behavior - Photometry correlation	S6d	Trials for lick vs % Trials for photometry	young APP-KI: 21 sessions	Pearson correlation	r(19) = 0.78<br>p = 2.5e-5		

Supplementary Table 1. Statistics used in this study (continued)

**Supplementary Fig. 7**							
Dopamine photometry<br>Diffrence between young WT and young APP-KI mice<br>(correct + error sessions)	S7a	T1	8 mice (young WT mice)<br>8 mice (young APP-KI mice)	1-way ANOVA<br>(Odor [A, B, 1, 2])	Group: F(3,28)=3.98; p=0.017	Tukey test	Odor A vs Odor 1: p=0.042<br>Odor B vs Odor 1: p=0.041<br>NS for other pairs
		T2			Group: F(3,28)=2.4; p=0.089		NS
		T3			Group: F(3,28)=1.24; p=0.31		NS
		T4			Group: F(3,28)=0.06; p=0.97		NS
		T5			Group: F(3,28)=1.55; p=0.22		NS
Dopamine photometry<br>Diffrence between young WT and young APP-KI mice<br>(correct sessions)	S7b	T1	8 mice (young WT mice)<br>8 mice (young APP-KI mice)	1-way ANOVA<br>(Odor [A, B, 1, 2])	Group: F(3,28)=4.41; p=0.011	Tukey test	Odor A vs Odor 1: p=0.018<br>Odor B vs Odor 1: p=0.036<br>NS for other pairs
		T2			Group: F(3,28)=0.94; p=0.43		NS
		T3			Group: F(3,28)=0.17; p=0.91		NS
		T4			Group: F(3,28)=0.15; p=0.93		NS
		T5			Group: F(3,28)=1.28; p=0.30		NS
Dopamine photometry<br>Diffrence between young WT and young APP-KI mice<br>(error sessions)	S7c	T1	8 mice (young WT mice)<br>8 mice (young APP-KI mice)	1-way ANOVA<br>(Odor [A, B, 1, 2])	Group: F(3,28)=0.44; p=0.72	Tukey test	NS
		T2			Group: F(3,28)=1.4; p=0.26		NS
		T3			Group: F(3,28)=1.04; p=0.39		NS
		T4			Group: F(3,28)=0.51; p=0.67		NS
		T5			Group: F(3,28)=0.69; p=0.56		NS
**Supplementary Fig. 8**							
Behavior-DA fiber stimulation	S8a	% Correct Trials in T5:<br>Control vs Stimulation	Young APP-KI: 11 mice	2-way ANOVA<br>(Odor type [A/B vs 1/2] x Stim [Control vs Stimulation])	Odor: F(3,80)=21.33; p=2.9e-10<br>Stim: F(1,80)=2.81; p=0.097<br>Odor x Stim: F(3,80)=4.48; p=0.0058	Tukey test	A Ctr vs B Ctr: p=1.9e-7<br>B Ctr vs 1 Ctr : p=9.8e-9<br>B Ctr vs 1 Stim: p=0.044<br>1 Ctr vs 2 Ctr: p=3.8e-7<br>1 Ctr vs A Stim: p=1.9e-6<br>1 Ctr vs B Stim: p=1.2e-8<br>1 Ctr vs 1 Stim: p=0.0034<br>1 Ctr vs 2 Stim: p=4.9e-7<br>NS for other pairs
Behavior-L-dopa treatment	S8b	% Correct Trials in T5:<br>WT mice vs APP-KI mice	Saline:8 mice<br>L-dopa: 9 mice	2-way ANOVA<br>(Odor type [A/B vs 1/2] x Drug type [Saline vs L-dopa])	Odor: F(3,60)=27.3; p=10e-13<br>Drug: F(1,60)=10.6; p=0.0018<br>Odor x Drug: F(3,60)=11.2; p=5.9e-6	Tukey test	odor A, Sal vs odor 1, Sal : p = 2.6e-10<br>odor B, Sal vs odor 1, Sal : p = 8.5e-13<br>odor B, Sal vs odor 1, ldopa : p = 1.7e-02<br>odor 1, Sal vs odor 2, Sal : p = 4.5e-11<br>odor 1, Sal vs odor A,ldopa : p = 2.2e-11<br>odor 1, Sal vs odor B,ldopa : p = 3.5e-13<br>odor 1, Sal vs odor 1,ldopa : p = 2.9e-07<br>odor 1, Sal vs odor 2,ldopa : p = 9.3e-11<br>odor B,ldopa vs odor 1,ldopa : p = 1.4e-02<br>NS for other pairs
**Supplementary Fig. 9**							
Behavior-DA fiber stimulation during all odor presentations	S9a2	% Correct Trials in T5:<br>Control vs Stimulation	Young APP-KI: 4 mice	2-way ANOVA<br>(Odor type [A/B vs 1/2] x Stim [Control vs Stimlation])	Odor: F(1,16)=11.88; p=0.033<br>Stim: F(1,16)=0.18; p=0.67<br>Odor x Stim: F(1,16)=0.55; p=0.47	Tukey test	A/B Ctr vs 1/2 Ctr: p=0.041<br>NS for other pairs
		% Correct Sessions in T5:<br>Control vs Stimulation		Binomial test	p=0.38		
	S9a3	% Correct Trials in T5:<br>Control vs Stimulation<br>(Odor A vs B vs 1 vs 2)		2-way ANOVA<br>(Odor type [A/B vs 1/2] x Stim [Control vs Stimlation])	Odor: F(3,32)=5.02; p=0.0058<br>Stim: F(1,32)=0.14; p=0.71<br>Odor x Stim: F(3,32)=0.78; p=0.51	Tukey test	1 Ctr vs B Stim: p=0.044<br>NS for other pairs
Behavior-DA fiber stimulation during odor 2 presentations	S9b2	% Correct Trials in T5:<br>Control vs Stimulation	Young APP-KI: 4 mice	2-way ANOVA<br>(Odor type [A/B vs 1/2] x Stim [Control vs Stimlation])	Odor: F(1,16)=14.86; p=0.0023<br>Stim: F(1,16)=27.17; p=2.1e-4<br>Odor x Stim: F(1,16)=15.83; p=0.018	Tukey test	A/B Ctr vs 1/2 Stim: p=0.0017<br>1/2 Ctr vs 1/2 Stim: p=1.4e-4<br>A/B Stim vs 1/2 Stim: p=0.0063<br>NS for other pairs
		% Correct Sessions in T5:<br>Control vs Stimulation		Binomial test	p ≈ 0		
	S9b3	% Correct Trials in T5:<br>Control vs Stimlation<br>(Odor A vs B vs 1 vs 2)		2-way ANOVA<br>(Odor type [A/B vs 1/2] x Stim [Control vs Stimlation])	Odor: F(3,24)=0.54; p=0.65<br>Stim: F(1,24)=3.78; p=0.063<br>Odor x Stim: F(3,24)=0.39; p=0.76	Tukey test	NS
	S9b3	% Correct Trials in T5:<br>Control vs Stimlation<br>(Odor A vs B vs 1 vs 2)			Odor: F(3,24)=0.75; p=0.75<br>Stim: F(1,24)=3.24; p=0.084<br>Odor x Stim: F(3,24)=0.38; p=0.77	Tukey test	NS
	S9b3	% Correct Trials in T5:<br>Control vs Stimlation<br>(Odor A vs B vs 1 vs 2)			Odor: F(3,24)=0.57; p=0.64<br>Stim: F(1,24)=3.65; p=0.068<br>Odor x Stim: F(3,24)=0.31; p=0.81	Tukey test	NS
	S9b3	% Correct Trials in T5:<br>Control vs Stimlation<br>(Odor A vs B vs 1 vs 2)			Odor: F(3,24)=0.70; p=0.56<br>Stim: F(1,24)=5.76; p=0.024<br>Odor x Stim: F(3,24)=0.52; p=0.67	Tukey test	NS
	S9b3	% Correct Trials in T5:<br>Control vs Stimlation<br>(Odor A vs B vs 1 vs 2)			Odor: F(3,24)=2.62; p=0.073<br>Stim: F(1,24)=3.91; p=0.059<br>Odor x Stim: F(3,24)=1.98; p=0.14	Tukey test	NS
**Supplementary Fig. 10**							
Behavior-L-dopa treatment in PS19 mice	S10b	% Correct Trials in T5:<br>Saline vs L-dopa	Saline: 5 mice<br>L-dopa: 5 mice	2-way ANOVA<br>(Odor type [A/B vs 1/2] x Drug [Saline vs L-dopa])	Odor: F(1,16)=24.96; p=1.3e-4<br>Drug: F(1,16)=5.8; p=0.028<br>Odor x Drug: F(1,16)=10.99; p=0.0044	Tukey test	A/B Sal vs 1/2 Sal: p=1.2e-4<br>1/2 Sal vs A/B dopa: p=1.2e-4<br>1/2 Sal vs 1/2 dopa: p=0.0047<br>NS for other pairs
		% Correct Sessions in T5:<br>Saline vs L-dopa		Binomial test	p=1.1e-6		
	S10c	% Correct Trials in T5:<br>Saline vs L-dopa<br>(Odor A vs B vs 1 vs 2)		2-way ANOVA<br>(Odor type [A vs B vs 1 vs 2] x Drug [Saline vs L-dopa])	Odor: F(3,32)=5.32; p=0.0044<br>Drug: F(1,32)=6.63; p=0.0148<br>Odor x Drug: F(3,32)=5.98; p=0.023	Tukey test	odor A Sal vs odor 1 Sal: p = 2.0e-3<br>odor B Sal vs odor 1 Sal: p = 8.9e-4<br>odor 1 Sal vs odor A dopa: p = 7.2e-4<br>odor 1 Sal vs odor B dopa: p = 9.8e-4<br>odor 1 Sal vs odor 1 dopa: p = 5.6e-4<br>odor 1 Sal vs odor 2 dopa: p = 8.0e-4<br>NS for other pairs
**Supplementary Fig. 11**							
Behavior-L-dopa treatment in WT mice	S11b	% Correct Trials in T5:<br>Saline vs L-dopa	Saline: 4 mice<br>L-dopa: 4 mice	2-way ANOVA<br>(Odor type [A/B vs 1/2] x Drug [Saline vs L-dopa])	Odor: F(1,4)=0.61; p=0.047<br>Drug: F(1,4)=0.03; p=0.86<br>Odor x Drug: F(1,4)=0.01; p=0.91	Tukey test	NS for all pairs
		% Correct Sessions in T5:<br>Saline vs L-dopa		Binomial test	p=1		
	S11c	% Correct Trials in T5:<br>Saline vs L-dopa<br>(Odor A vs B vs 1 vs 2)		2-way ANOVA<br>(Odor type [A vs B vs 1 vs 2] x Drug [Saline vs L-dopa])	Odor: F(3,.8)=2.81; p=0.0044<br>Drug: F(1,8)=0.82; p=0.0148<br>Odor x Drug: F(3,8)=0.012; p=0.99	Tukey test	NS for all pairs

Supplementary Table 2. Animals used in this study

Figure #	Genotype	Experiment	Virus or Drug Injected	Animal ID	Sex	Implanted Device
Fig. 1, S1	Old WT	Behavior test	None	991	M	None
				1293	F	
				1417	M	
				1638	M	
				1639	M	
				1640	M	
				1641	F	
	Young APP-KI	Behavior test	None	815	M	None
				1191	M	
				1193	F	
				1425	F	
				1426	F	
	Old APP-KI	Behavior test	None	692	F	None
				926	F	
				934	F	
				989	M	
				1247	F	
				1294	M	
				1421	F	
				1452	F	
				1479	M	
Fig.1, 2, E4-7, S1,S4	Young WT	LEC L2/3 cells recording (right hemisphere)	None	364	M	Tetrode drive (64 channels)
				375	F	
				390	F	
				1398	F	
				1431	M	
	Young APP-KI	LEC L2/3 cells recording (right hemisphere)	None	247	M	Tetrode drive (64 channels)
				690	F	
				703	M	
				925	M	
				941	M	
Fig. 1, 4, S1, S6, S7	DAT-Cre	Dopamine photometry (left hemisphere* or bilateral)	AAV-flex-GCaMP6 @ VTA/SNc (0.75-1μL)	163*	M	Optic fibers (400μm)
				164*	M	
				263	F	
				264	F	
				265	M	
				266	M	
	APP-KI x DAT-Cre	Dopamine photometry (right hemisphere* or bilateral)	AAV-flex-GCaMP6 @ VTA/SNc (0.75-1μL)	1028	F	Optic fibers (400μm)
				1029	F	
				1416	F	
				1451*	M	
				1477*	M	

Supplementary Table 2. Animals used in this study (continued)

Fig.5, S8	APP-KI x DAT-Cre	Dopaminergic fiber stimulation (bilateral)	AAV-DIO-ChR2-mCherry @ VTA/SNc or AAV-DIO-ChR2-eYFP @ VTA/SNc (1µL)	1167	M	Optic fibers (400µm)
				1170	M	
				1183	M	
				1194	F	
				1195	F	
				1380	F	
				1414	F	
				1583	M	
				1585	F	
				1588	F	
				1601	M	
Fig.6, S8	APP-KI	L-dopa treatment (control)	Saline injection (i.p.)	2017	M	None
				2018	M	
				2019	M	
				2020	F	
				2021	F	
				2022	F	
				2023	F	
				2024	F	
	APP-KI	L-dopa treatment (L-dopa)	L-dopa injection (50mg/kg, i.p.)	2178	M	None
				2179	M	
				2180	F	
				2182	F	
				2243	M	
				2244	M	
				2246	M	
				2248	F	
				2250	F	
Fig.6	APP-KI	L-dopa treatment (control)	Saline injection (i.p.)	2490	M	Tetrode drive (64 channels)
				2491	M	
				2492	F	
				2493	F	
		L-dopa treatment (L-dopa)	L-dopa injection (50mg/kg, i.p.)	2427	M	Tetrode drive (64 channels)
				2474	M	
				2475	F	
				2477	F	
Fig. 1, E9,S5	Old WT	LEC L2/3 cells recording (right hemisphere)	None	210	M	Tetrode drive (64 channels)
				310	F	
				346	M	
				387	M	
	Old APP-KI	LEC L2/3 cells recording (right hemisphere)	None	789	F	Tetrode drive (64 channels)
				1199	M	
				1253	M	
				1254	M	
				1517	M	

Supplementary Table 2. Animals used in this study (continued)

Fig. S9	APP-KI x DAT-Cre	Dopaminergic fiber stimulation (bilateral)	AAV-DIO-ChR2-eYFP @ VTA/SNc (1μL)	2442	M	Optic fibers (400μm)
				2454	M	
				2455	M	
				2483	F	
				2484	F	
Fig. S9	APP-KI x DAT-Cre	Dopaminergic fiber stimulation (bilateral)	AAV-DIO-ChR2-eYFP @ VTA/SNc (1μL)	2514	M	Optic fibers (400μm)
				2515	M	
				2512	F	
				2516	F	
Fig. 6, S10	PS19	L-dopa treatment (control)	Saline injection (i.p.)	2425	F	None
				2426	F	
				2456	M	
				2499	M	
				2500	M	
		L-dopa treatment (L-dopa)	L-dopa injection (50mg/kg, i.p.)	2010	M	
				2011	M	
				2012	F	
				2013	F	
				2424	F	
Fig. S11	Young WT	L-dopa treatment (control)	Saline injection (i.p.)	2459	M	None
				2460	M	
				2510	F	
				2520	F	
		L-dopa treatment (L-dopa)	L-dopa injection (50mg/kg, i.p.)	2432	M	
				2433	M	
				2503	F	
				2521	F	

Supplementary Table 3. Odors used in this study

Pair #	Odor ID	Chemical Nomenclature	CAS	Company	Solvent	Concentration Used
1	1	isoamyl acetate	123-92-2	Acros Organics	mineral oil	5%
	2	alpha-pinene	7785-26-4	Acros Organics	mineral oil	15%
2	3	geraniol	106-24-1	Sigma	mineral oil	10.00%
	4	trans-Anethole	4180-23-8	Sigma	mineral oil	5.00%
3	5	Ethyl Butyrate	105-54-4	Acros Organics	propylene glycol	5.00%
	6	1-hexanol	111-27-3	Acros Organics	propylene glycol	5.00%
4	7	vanillin	121-33-5	Chem-Impex	ethylene glycol	40.00%
	8	d-Limonene	5989-27-5	MP Biomedicals	mineral oil	5.00%
5	9	alpha-ionone	127-41-3	Alfa Aesar	mineral oil	20.00%
	10	beta-phenylethyl alcohol	60-12-8	TCI	mineral oil	1.00%
6	11	3-octanone	106-68-3	Sigma	mineral oil	5.00%
	12	octanol	111-87-5	Fisher	mineral oil	1.00%
7	13	g-Undecalactone	104-67-6	Sigma	-	100.00%
	14	10CHO (decanal)	112-31-2	TCI	mineral oil	1.00%
8	15	lilial	80-54-6	MP Biomedicals	mineral oil	2.00%
	16	linalool	78-70-6	TCI	mineral oil	10.00%
9	17	l-carvone	6485-40-1	TCI	mineral oil	10.00%
	18	citral	5392-40-5	TCI	mineral oil	10.00%
10	19	cumen aldehyde	122-03-2	TCI / Sigma	mineral oil	10.00%
	20	cinnamic alcohol	104-54-1	TCI	propylene glycol	10.00%
11	21	eugenol	97-53-0	TCI	mineral oil	10.00%
	22	Propyl propionate	106-36-5	TCI	mineral oil	10.00%
12	23	Menthol	2216-51-5	TCI	mineral oil	50.00%
	24	3-octanone	106-68-3	TCI	propylene glycol	2.50%
13	25	coumarin	91-64-5	TCI	propylene glycol	25.00%
	26	phenyl ethyl acetate	103-45-7	TCI	propylene glycol	5.00%
14	27	2-Pentanone	107-87-9	Alfa Aesar	mineral oil	5.00%
	28	(+)-Camphor	464-49-3	TCI	mineral oil	25.00%
15	29	Methyl 3-hexenoate	2396-78-3	Acros Organics	mineral oil	0.33%
	30	trans-Cinnamaldehyde	104-55-2	Chem-Impex	mineral oil	5.00%
16	31	cedrol	77-53-2	TCI	propylene glycol	15.00%
	32	methyl hexanoate	106-70-7	TCI	mineral oil	5.00%
17	33	terpinyl acetate	80-26-2	TCI	mineral oil	20.00%
	34	trans-2-hexenal	6728-26-3	Sigma	mineral oil	1.00%
18	35	musk T	105-95-3	TCI	mineral oil	10.00%
	36	octyl acetate	112-14-1	TCI	mineral oil	10.00%
19	37	nonaic acid	112-05-0	TCI	mineral oil	5.00%
	38	methyl heptanone	541-85-5	TCI	mineral oil	5.00%
20	39	bornyl acetate	5655-61-8	TCI	mineral oil	10.00%
	40	octanal	124-13-0	TCI	mineral oil	1.00%
21	41	cis-jasmone	488-10-8	TCI	mineral oil	20.00%
	42	benzyl alcohol	100-51-6	TCI	propylene glycol	40.00%
22	43	nonyl alcohol	143-08-8	TCI	mineral oil	10.00%
	44	celestolide	13171-00-1	TCI	mineral oil	10.00%
23	45	farnesol	4602-84-0	Sigma	mineral oil	33.00%
	46	mentyl acetate	2623-23-6	Sigma	mineral oil	33.00%
24	47	ethyl methanoate	109-94-4	TCI	mineral oil	10.00%
	48	citral dimethyl acetal	7549-37-3	TCI	mineral oil	10.00%
25	49	cis-6-nonen-1-ol	35854-86-5	Alfa Aesar	mineral oil	10.00%
	50	mehyl salicylate	119-36-8	TCI	mineral oil	5.00%
26	51	gamma-valerolactone	108-29-2	TCI	mineral oil	10.00%
	52	ethyl hexanoate	123-66-0	TCI	mineral oil	10.00%
27	53	cineol	470-82-6	Sigma	mineral oil	10.00%
	54	cinnamic acid	140-10-3	TCI	mineral oil	10.00%
28	55	cis-3-Hexanol	928-96-1	Frontier Scientific	mineral oil	10.00%
	56	pipertone	89-81-6	TCI	mineral oil	10.00%
29	57	catechol	120-80-9	TCI	mineral oil	10.00%
	58	geranyl acetate	105-87-3	TCI	mineral oil	10.00%
30	59	Exaltolide	106-02-5	TCI	mineral oil	10.00%
	60	anisyl alcohol	105-13-5	Frontier Scientific	mineral oil	10.00%
31	61	acetophenone	98-86-2	Frontier Scientific	mineral oil	10.00%
	62	citronellol	106-22-9	Frontier Scientific	mineral oil	10.00%
32	63	geranyl nitrile	5146-66-7	TCI	mineral oil	10.00%
	64	benzylidene acetone	122-57-6	Frontier Scientific	mineral oil	10.00%
33	65	thymol	89-83-8	Frontier Scientific	mineral oil	10.00%
	66	musk ambrette	83-66-9	TCI	mineral oil	10.00%
34	67	hexenyl acetate	3681-71-8	Frontier Scientific	mineral oil	10.00%
	68	anisaldehyde	123-11-5	Frontier Scientific	propylene glycol	10.00%

Supplementary Table 4. Other resources used in this study

Type	Mouse Line / Product	Source	Identifier
Mouse	APP Knock-in	RIKEN Bio Resource Center	RBRC06344
	DAT-Cre	Jackson Laboratory	006660
	C57BL/6J	Jackson Laboratory	000664
Virus	AAV5-DIO-ChR2-eYFP	UNC Vector Core	N/A
	AAV5-DIO-ChR2-mCherry	UNC Vector Core	N/A
	AAV5-flex-GCAMP6m	Addgene	100839
Antibody	Anti Tyrosine Hydoxylase (rabbit)	Millipore	MB152
	Alexa Fluor 488 Anti-rabbit	Abcam	ab150077
	Anti GFP (chicken)	Aveslab	GFP-1010
	Alexa Fluor 488 Anti-chicken	Invitrogen	A-11039
	Anti-α-Aβ (mouse)	MediMabs	MM-0015-1P
	Alexa Fluor 488 Anti-mouse	Thermo Fisher	A-11001
	DAPI-Fluoromont-G mounting medium	SouthernBiotech	0100-20
Compound	L-3,4-dihydroxyphenylalanine (L-DOPA)	TCI	D0600
