# Supplementary material for: Early dopamine disruption in the entorhinal cortex of a knock-in model of Alzheimer’s disease
Source: Nat Neurosci. 2026 Apr 23;29(6):1386–96. doi: 10.1038/s41593-026-02260-w (PMC13246448; doi:10.1038/s41593-026-02260-w)
Supplement: Supplementary file 2 — Reporting Summary [file 41593_2026_2260_MOESM2_ESM.pdf]

nature portfolio

Corresponding author(s): Kei M Igaarshi

Last updated by author(s): Feb 4, 2026

Reporting Summary

Nature Portfolio wishes to improve the reproducibility of the work that we publish. This form provides structure for consistency and transparency in reporting. For further information on Nature Portfolio policies, see our Editorial Policies and the Editorial Policy Checklist.

Statistics

For all statistical analyses, confirm that the following items are present in the figure legend, table legend, main text, or Methods section.

n/a	Confirmed	
☐	☒	The exact sample size (*n*) for each experimental group/condition, given as a discrete number and unit of measurement
☐	☒	A statement on whether measurements were taken from distinct samples or whether the same sample was measured repeatedly
☐	☒	The statistical test(s) used AND whether they are one- or two-sided<br>*Only common tests should be described solely by name; describe more complex techniques in the Methods section.*
☐	☒	A description of all covariates tested
☐	☒	A description of any assumptions or corrections, such as tests of normality and adjustment for multiple comparisons
☐	☒	A full description of the statistical parameters including central tendency (e.g. means) or other basic estimates (e.g. regression coefficient) AND variation (e.g. standard deviation) or associated estimates of uncertainty (e.g. confidence intervals)
☐	☒	For null hypothesis testing, the test statistic (e.g. *F*, *t*, *r*) with confidence intervals, effect sizes, degrees of freedom and *P* value noted<br>*Give P values as exact values whenever suitable.*
☒	☐	For Bayesian analysis, information on the choice of priors and Markov chain Monte Carlo settings
☒	☐	For hierarchical and complex designs, identification of the appropriate level for tests and full reporting of outcomes
☒	☐	Estimates of effect sizes (e.g. Cohen's *d*, Pearson's *r*), indicating how they were calculated

Our web collection on statistics for biologists contains articles on many of the points above.

Software and code

Policy information about availability of computer code

Data collection	Cheetah v 5.7.1 and v 6.1 (Neuralynx Inc), LabView v2018 (National Instruments)
Data analysis	Matlab R2022a (Mathworks), Image J (NIH)

For manuscripts utilizing custom algorithms or software that are central to the research but not yet described in published literature, software must be made available to editors and reviewers. We strongly encourage code deposition in a community repository (e.g. GitHub). See the Nature Portfolio guidelines for submitting code & software for further information.

Data

Policy information about availability of data

All manuscripts must include a data availability statement. This statement should provide the following information, where applicable:

- Accession codes, unique identifiers, or web links for publicly available datasets
- A description of any restrictions on data availability
- For clinical datasets or third party data, please ensure that the statement adheres to our policy

All data can be obtained from the repository at www.igarashilab.org/

Research involving human participants, their data, or biological material

Policy information about studies with human participants or human data. See also policy information about sex, gender (identity/presentation), and sexual orientation and race, ethnicity and racism.

Reporting on sex and gender	No human data was obtained in the study.
Reporting on race, ethnicity, or other socially relevant groupings	*Please specify the socially constructed or socially relevant categorization variable(s) used in your manuscript and explain why they were used. Please note that such variables should not be used as proxies for other socially constructed/relevant variables (for example, race or ethnicity should not be used as a proxy for socioeconomic status).*<br>*Provide clear definitions of the relevant terms used, how they were provided (by the participants/respondents, the researchers, or third parties), and the method(s) used to classify people into the different categories (e.g. self-report, census or administrative data, social media data, etc.)*<br>*Please provide details about how you controlled for confounding variables in your analyses.*
Population characteristics	*Describe the covariate-relevant population characteristics of the human research participants (e.g. age, genotypic information, past and current diagnosis and treatment categories). If you filled out the behavioural & social sciences study design questions and have nothing to add here, write "See above."*
Recruitment	*Describe how participants were recruited. Outline any potential self-selection bias or other biases that may be present and how these are likely to impact results.*
Ethics oversight	*Identify the organization(s) that approved the study protocol.*

Note that full information on the approval of the study protocol must also be provided in the manuscript.

Field-specific reporting

Please select the one below that is the best fit for your research. If you are not sure, read the appropriate sections before making your selection.

☒ Life sciences ☐ Behavioural & social sciences ☐ Ecological, evolutionary & environmental sciences

For a reference copy of the document with all sections, see nature.com/documents/nr-reporting-summary-flat.pdf

Life sciences study design

All studies must disclose on these points even when the disclosure is negative.

Sample size	No statistical method was used to pre-determine sample sizes, but we chose sample sizes that exceeded or matched those reported in previous studies with similar methodologies. Both males and females were included in the samples. We did not find any difference between sexes, thus data from males and females were combined and reported in the study.
Data exclusions	Criteria for data exclusion based on lack of viral expression or miss-targeting of injection/implantation were established prior to the experiments. Otherwise, no systematic data exclusion was performed.
Replication	The reported findings were reproduced across animals. The total number of animals and neurons is reported for all experiments in the statistical data summary sheet.
Randomization	For all experiments mice were randomly assigned as to which implantation they received, the virus injection location and protocol used.
Blinding	Experimenters were blind to genotype, region targeted and optogenetic protocol in all experiments and analyses.

Reporting for specific materials, systems and methods

We require information from authors about some types of materials, experimental systems and methods used in many studies. Here, indicate whether each material, system or method listed is relevant to your study. If you are not sure if a list item applies to your research, read the appropriate section before selecting a response.

Materials & experimental systems

n/a	Involved in the study
☐	☒ Antibodies
☒	☐ Eukaryotic cell lines
☒	☐ Palaeontology and archaeology
☐	☒ Animals and other organisms
☒	☐ Clinical data
☒	☐ Dual use research of concern
☒	☐ Plants

Methods

n/a	Involved in the study
☒	☐ ChIP-seq
☒	☐ Flow cytometry
☒	☐ MRI-based neuroimaging

Antibodies

Antibodies used

Tyrosine hydroxylase antibody (MB152, Millipore, LOT:3510772)
Amyloid Beta Mouse Monoclonal Antibody (McSA1, MEDIMABS, LOT: 1541300110-P)
Green Fluorescent Protein Antibody (GFP-1010 aveslabs, LOT: GFP879484)
Alexa Fluor 488 anti-rabbit (ab150077, abcam, LOT: 3377607-1)
Alexa Fluor 488 anti-chicken (A-11039, Invitrogen, LOT: 1899514)
Alexa Fluor 488 anti-mouse (A-11001, Invitrogen, LOT: 2247988)

Validation

The antibodies used in this study are established antibodies used frequently in previous studies. We refer to the websites of resource companies for their validations:

anti-tyrosine hydroxylase
https://www.emdmillipore.com/US/en/product/Anti-Tyrosine-Hydroxylase-Antibody,MM_NF-AB152?ReferrerURL=https%3A%2F%2Fwww.google.com%2F

anti-Abeta
https://medimabs.com/product/amyloid-beta-mouse-monoclonal-antibody-mcsa1/

anti-GFP
https://www.antibodiesinc.com/products/anti-green-fluorescent-protein-antibody-gfp?variant=13128185544763

Alexa Fluor 488 anti-rabbit
https://www.abcam.com/en-us/products/secondary-antibodies/goat-rabbit-igg-h-l-alexa-fluor-488-ab150077

Alexa Fluor 488 anti-chicken
https://www.thermofisher.com/antibody/product/Goat-anti-Chicken-IgY-H-L-Secondary-Antibody-Polyclonal/A-11039

Alexa Fluor 488 anti-mouse
https://www.thermofisher.com/antibody/product/Goat-anti-Mouse-IgG-H-L-Cross-Adsorbed-Secondary-Antibody-Polyclonal/A-11001

Animals and other research organisms

Policy information about studies involving animals; ARRIVE guidelines recommended for reporting animal research, and Sex and Gender in Research

Laboratory animals

APP knock-in mice (NL-G-F, RIKEN BioResouce Research Center, RBRC06344), DAT-Cre mice (Jackson Lab, 006660) C57BL/6J mouse (Jackson Lab, 000664). Animals were used in 3-13 months of age. Both females and males were included (see animal summary sheet). PS19 mice are from Jackson Lab (008169).

Wild animals

N/A

Reporting on sex

Both males and females were included in the study. See summary table for sex of individual subjects. No difference between males and females was observed in preliminary analyses.

Field-collected samples

N/A

Ethics oversight

Institutional Animal Care and Use Committee at the University of California, Irvine.

Note that full information on the approval of the study protocol must also be provided in the manuscript.

Plants

Seed stocks	No plants were used.
Novel plant genotypes	*Describe the methods by which all novel plant genotypes were produced. This includes those generated by transgenic approaches, gene editing, chemical/radiation-based mutagenesis and hybridization. For transgenic lines, describe the transformation method, the number of independent lines analyzed and the generation upon which experiments were performed. For gene-edited lines, describe the editor used, the endogenous sequence targeted for editing, the targeting guide RNA sequence (if applicable) and how the editor was applied.*
Authentication	*Describe any authentication procedures for each seed stock used or novel genotype generated. Describe any experiments used to assess the effect of a mutation and, where applicable, how potential secondary effects (e.g. second site T-DNA insertions, mosiacism, off-target gene editing) were examined.*
